# AIG1 protects against doxorubicin-induced cardiomyocyte ferroptosis and cardiotoxicity by promoting ubiquitination-mediated p53 degradation

**DOI:** 10.7150/thno.108410

**Published:** 2025-03-31

**Authors:** Yuekai Shi, Jieru Cai, Lu Chen, Hao Cheng, Xiaoyue Song, Junqiang Xue, Rende Xu, Jianying Ma, Junbo Ge

**Affiliations:** 1 Department of Cardiology, Zhongshan Hospital, Fudan University, Shanghai Institute of Cardiovascular Diseases, Shanghai 200032, China.; 2 State Key Laboratory of Cardiovascular Diseases, Zhongshan Hospital, Fudan University, China.; 3 National Clinical Research Center for Interventional Medicine, Shanghai 200032, China.; 4 Institutes of Biomedical Sciences, Fudan University, Shanghai, China.; 5 Department of Nephrology, Zhongshan Hospital, Fudan University, Shanghai 200032, China.

**Keywords:** Doxorubicin-induced cardiotoxicity, Ferroptosis, AIG1, Ubiquitination, p53

## Abstract

**Background**: Doxorubicin (DOX) is a widely employed chemotherapeutic drug, while its clinical use is limited by the lethal cardiotoxicity. Previous studies highlighted the critical role of cardiomyocyte ferroptosis in the pathogenesis of DOX-induced cardiotoxicity (DIC). Androgen-induced gene 1 (AIG1) is perceived as a key regulator of oxidative stress-mediated cell death. Nonetheless, it remains elusive whether AIG1 is involved in the progression of DOX-induced cardiomyocyte ferroptosis and cardiotoxicity.

**Methods**: C57BL/6 male mice were repeatedly administrated with DOX at an accumulative dosage of 20 mg/kg to establish a chronic DIC model. Global AIG1 knockout mice and AAV9-mediated cardiac-specific AIG1 knockdown or overexpression mice were utilized to evaluate the precise role of AIG1 in DIC. Additionally, the effects of AIG1 on cardiomyocyte ferroptosis were further investigated following DOX stimulation.

**Results**: Ferroptosis played a pivotal role in DIC in both *in vivo* and *in vitro* settings. DOX exposure significantly reduced AIG1 expression levels in cardiomyocytes. Global AIG1 knockout or cardiac-specific AIG1 knockdown mice exhibited deteriorated cardiac function, adverse cardiac remodeling following DOX insult. Moreover, AIG1 deficiency aggravated DOX-evoked ferroptosis and oxidative stress in cardiomyocytes, whereas cardiac-specific overexpression of AIG1 conferred the protective effects manifested by the inhibition of cardiomyocyte ferroptosis and improvements in cardiac performance and remodeling under DOX challenge. Mechanistically, AIG1 directly interacted with the Pirh2 E3 ubiquitin ligase to promote the ubiquitination of p53, a key protein governing ferroptosis during DIC, thereby accelerating its degradation. Cardiac-specific Pirh2 knockdown markedly exacerbated DOX-induced ferroptosis by enhancing p53 activity in cardiomyocytes. Furthermore, the pharmacological administration of a highly selective p53 inhibitor PFT-α effectively ameliorated DIC in mice by inhibiting cardiomyocyte ferroptosis and substantially abrogated the deleterious cardiac effects associated with AIG knockout under DOX challenge.

**Conclusion**: Our findings defined the critical cardioprotective role of AIG1 in DIC by alleviating cardiomyocyte ferroptosis in a Pirh2/p53 axis-dependent manner. Targeting the novelly identified AIG1-Pirh2-p53 signaling axis presents a promising approach to prevent DIC.

## Introduction

Doxorubicin is an anthracycline antitumor drug for widely treating multiple hematopoietic malignancies and solid tumor 1, 2. Unfortunately, the clinical application of DOX is largely impeded due to cumulative and irreversible cardiotoxicity 2, characterized by ventricular dilation and systolic dysfunction, and eventually progressing into congestive heart failure 2-4. Significantly, the onset of DOX-induced cardiotoxicity (DIC) is strongly associated with a poor prognosis and an elevated risk of mortality in cancer survivors 5, 6. To date, although researchers have proposed several potential mechanisms responsible for the onset and development of DIC, including DNA damage, oxidative stress, mitochondrial toxicity and different forms of cardiomyocyte death 2, 7, 8, there remain few clinically effective therapeutic strategies employed for managing DIC 1, 9. Hence, it is pertinent to elucidate the complicated molecular mechanisms underlying DIC and identify novel treatment targets.

Ferroptosis is an iron-dependent form of programmed cell death manifested as intracellular iron accumulation, glutathione depletion, a burst of reactive oxygen species (ROS) and lipid peroxidation 10-12. A growing body of evidence has recently demonstrated that cardiomyocyte ferroptosis plays a critical role in the development of various cardiovascular diseases 11, such as myocardial infarction, cardiac ischemia/reperfusion injury, and DIC 13-18. DOX can directly trigger myocardial iron overload by disrupting intracellular normal iron transport system 13, 17, and also can downregulate glutathione peroxidase 4 (GPX4), a key enzyme detoxifying peroxidized lipids 19, leading to excessive lipid peroxidation and ferroptosis in cardiomyocytes, ultimately resulting in cardiac damage and dysfunction 15. Importantly, emerging preclinical studies 13, 15-17 have indicated that several small molecule compounds capable of inhibiting ferroptosis, such as ferrostatin-1 (Fer-1; a lipid peroxide scavenger), deferoxamine (DFO; an iron chelator), or dexrazoxane (DXZ; the only drug approved by the US Food and Drug Administration to DIC treatment partially via iron chelating) 20, exhibited promising therapeutic efficacy in animal models of DIC. These findings suggest an essential involvement of cardiomyocyte ferroptosis in the pathogenesis of DIC. Nevertheless, the precise mechanism by which DOX induced iron overload, oxidative damage and subsequent ferroptosis in the heart remains largely undefined. Therefore, identifying of specific molecules and pathways involved in DOX-induced ferroptosis in cardiomyocyte is crucial for developing therapeutic targets to prevent DIC.

It is well established that DOX-induced p53 activation plays a significant role in the development of DIC 21, 22. p53 not only potentially regulates cardiomyocyte apoptosis and autophagy by upregulating cell death receptors in DIC, but also acts as a crucial governor of cardiomyocyte ferroptosis during DIC 22. Specifically, p53 directly inhibits the expression of solute carrier family 7 member 11 (SLC7A11), a subunit of system Xc^ -^ responsible for cystine transport, thereby impairing glutathione (GSH) synthesis and ultimately reducing GPX4 activity, which compromises cellular antioxidant defenses and promotes ferroptosis in DOX-stressed cardiomyocytes 22-24. In addition, p53 has been suggested to play an instrumental role in directly exacerbating DOX-mediated iron homeostasis dysregulation in cardiomyocytes during DIC via a post-translational mechanism that involves the regulation of iron-sulfur (Fe-S) clusters/iron regulatory protein-iron response element (IRP-IRE) pathway 17. Therefore, targeting p53 signaling may represent a crucial strategy to mitigate DOX-induced cardiomyocyte ferroptosis and cardiotoxicity.

Androgen-induced gene 1, called AIG1, is a member of androgen-inducible genes family, which can be regulated by androgen level 25. AIG1 is mostly known to function as a specific endogenous hydrolase 26, 27 of anti-inflammatory and anti-diabetic fatty acid esters of hydroxy fatty acid (FAHFA) 28-30 and is therefore closely involved in the regulation of obesity and other metabolic disorders 31. Prior studies have demonstrated that AIG1 can activate the nuclear factor of activated T cells signaling pathway 32, exerting proliferative and survival-promoting effects 33, 34. Besides, AIG1 can modulate endoplasmic reticulum (ER) and intracellular Ca2+ homeostasis, as well as cell death susceptibility against oxidative stress 35, which is recognized as a critical regulator of cell death signaling 25, 32, 35. Notably, ferroptosis is exactly an oxidative form of regulated cell death, of which the pathological mechanisms are closely aligned with cellular oxidative damage 10-12. Moreover, AIG1 has been reported to increase p53 reporter gene activity, possibly through its interaction with ring finger and CHY zinc finger domain containing 1 (RCHY1, also known as Pirh2) E3 ubiquitin ligase in cancer cells 32. Interestingly, Pirh2 functions as a key regulator of the p53 signaling pathway, directly ubiquitinating p53 and inhibiting p53 activity 36. Collectively, it is conceivable that AIG1 is likely to participate in the regulation of DOX-induced ferroptosis during DIC. However, the precise roles and interaction dynamics of AIG1 and Pirh2/p53 axis in cardiovascular diseases, including the clinical scenario of DIC, where disrupted cellular redox hemodynamics and iron homeostasis induced-cardiomyocyte ferroptosis is a core pathological process, remain elusive.

In the present study, we aimed to explore the specific role of AIG1 in DOX-induced cardiomyocyte ferroptosis and cardiotoxicity, and the underlying mechanism involved with a particular focus on the modulation of Pirh2/p53 signaling axis. Here, we provided evidence for the first time that the expression of AIG1 is significantly decreased in DOX-stressed cardiomyocytes. Our results revealed that the downregulated AIG1 impairs Pirh2 E3 ligase-mediated ubiquitination and degradation of p53 by disrupting AIG1-Pirh2 interaction, resulting in p53 activation, which triggers cardiomyocyte ferroptosis and oxidative stress. In contrast, cardiac-specific overexpression of AIG1 effectively prevents DOX-induced ferroptosis and cardiotoxicity. Further scrutiny highlighted that Pirh2/p53 axis is responsible for the anti-ferroptosis effects of AIG1 in DIC. We demonstrated that Pirh2 deficiency can exacerbate DOX-induced ferroptosis and dampen the cardioprotective effects of AIG1 overexpression in DOX stressed-cardiomyocytes by enhancing p53 activity. In addition, pharmacological inhibition of p53 employing a selective p53 inhibitor notably alleviates DIC and reverses AIG knockout-induced aggravated myocardial damage upon DOX challenge. Together, these findings identified the significance of AIG1-Pirh2-p53 signaling axis in DOX-induced cardiomyocyte ferroptosis and its implications for preventing DIC.

## Methods

All data, study methods and materials that support the findings of this study are available from the corresponding authors on reasonable request. Detailed methods are provided in the Supplemental Material.

### Animal studies

Global AIG1 knockout mice and their littermate wild-type control mice on C57BL/6 background, aged 8-10 weeks, were purchased by GemPharmatech Co., Ltd (Jiangsu, China). Heart-restricted overexpression or knockdown of AIG1 or Pirh2 mice were generated by injecting C57BL/6 mice with adeno-associated virus serotype 9 (AAV9) driven by the cardiac troponin T promoter (Hanbio Biotechnology Co., Ltd. Shanghai, China) via the tail vein. All animal studies complied with the National Institutes of Health Guidelines for the Care and Use of Laboratory Animals, and permitted by the Animal Care and Use Committee of Zhongshan Hospital, Fudan University.

### Statistical analysis

All data were shown as mean ± SEM. Statistical analysis was performed using the GraphPad Prism 9.4.1 software (GraphPad Software, La Jolla, CA, USA). Comparison between the two groups was conducted using unpaired student's t-test (normal distribution and equal variances). Multiple group comparisons were performed using one-way or two-way ANOVA (normal distribution) followed by Tukey's multiple comparison test. Differences between groups were classified as not statistically significant (ns), statistically significant (*P < 0.05), high significant (**P < 0.01), very significant (***P < 0.001) and the most significant (****P < 0.0001). In certain cases, raw values were normalized to the control group values. All experimental n numbers are provided in the figure legends.

## Results

### Ferroptosis is activated in DOX-stressed cardiomyocytes

**Figure 1:**
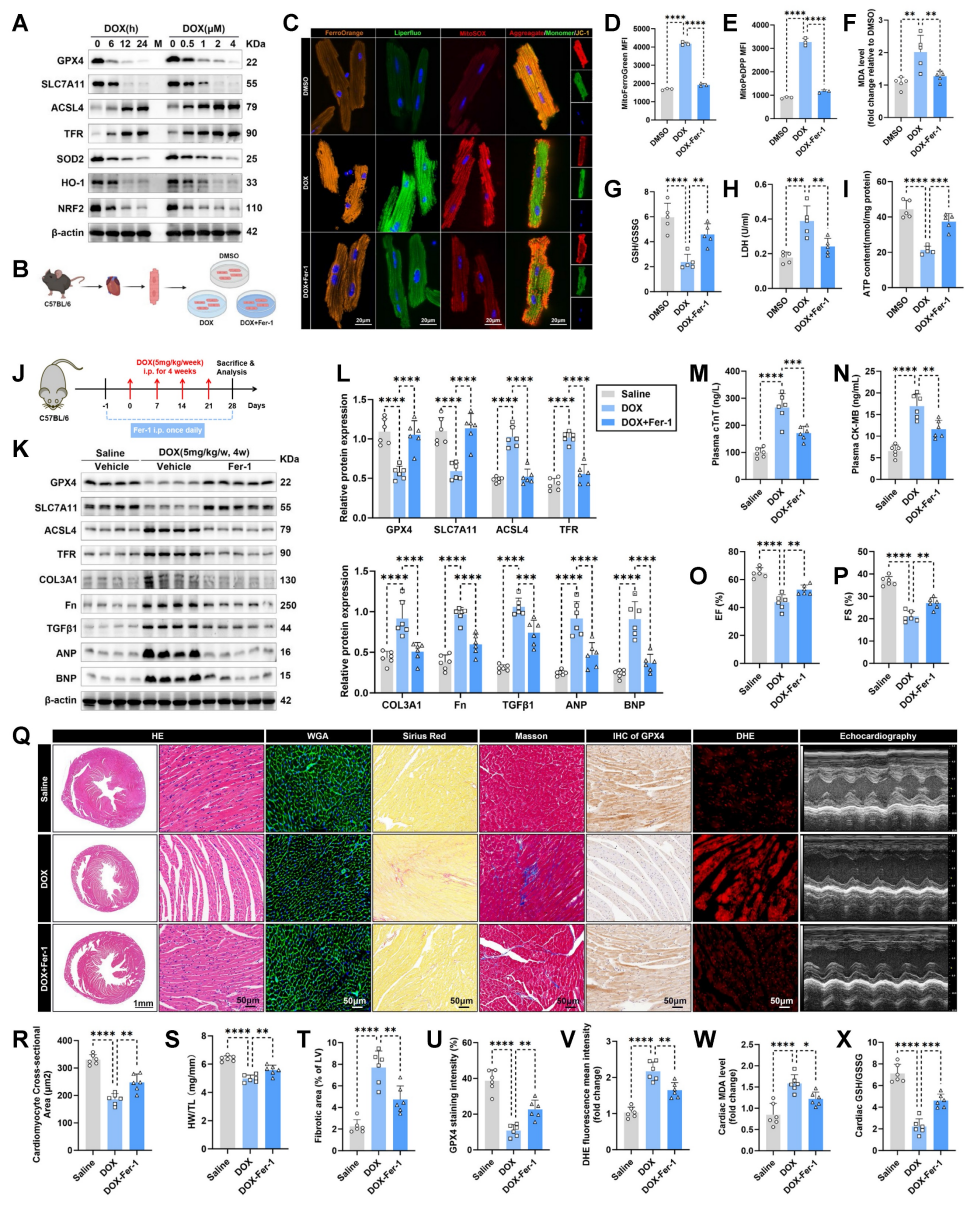
** Ferroptosis is activated in DOX-stressed cardiomyocytes. (A)** Representative immunoblotting images of time sequence of ferroptosis and oxidative stress development in HL-1 cardiomyocytes treated with Doxorubicin (DOX, 1 μM) for different times or with various concentrations of DOX for 24 hours. **(B)** Experimental design for DOX-induced cardiotoxicity (DIC) in adult mouse cardiomyocytes (AMCMs), and Fer-1 was used for anti-ferroptosis treatment prior to DOX challenge. **(C)** Representative fluorescence images of intracellular iron (FerroOrange staining), lipid peroxide (Liperfluo staining), mitochondrial superoxide (MitoSOX staining), and mitochondrial membrane potential (JC-1 staining) in AMCMs with indicated treatment. Nuclei were counterstained with Hoechst 33342 (blue). **(D)** The mean fluorescence intensities (MFI) of MitoFerroGreen staining indicated Mitochondrial iron level using flow cytometry in HL-1 cardiomyocytes with indicated treatment. (n = 3 per group). **(E)** The MFI of MitoPeDPP staining indicated mitochondrial lipid peroxide in HL-1 cardiomyocytes with indicated treatment and quantitative analysis of the MFI using flow cytometry. (n = 3 per group). **(F)** and **(G)** Malondialdehyde (MDA) levels and GSH/GSSG ratio in HL-1 cardiomyocytes with indicated treatment. (n = 5 per group). **(H)** and **(I)** LDH release and ATP content in HL-1 cardiomyocytes with indicated treatment (n = 5 per group). **(J)** Experimental design for the chronic DIC mouse model in C57BL/6 mice, and Fer-1 (1 mg/kg/day) was administered via intraperitoneal (i.p.) injection as an anti-ferroptosis treatment during the DIC model. **(K)** and **(L)** The heart tissues were examined by Western blots and statistical analysis. (n = 6 per group).** (M)** and **(N)** The levels of plasma cardiac troponin T (cTnT) and creatine kinase-myocardial band (CK-MB) in mice. (n = 6 per group).** (O)** through** (Q)** Quantification of left ventricular ejection fraction (LVEF, EF) **(O)** and left ventricular fraction shortening (LVFS, FS) **(P)** with representative M-mode images **(Q)** from transthoracic echocardiography. (n = 6 per group).** (Q)** and **(R)** Representative images of hematoxylin and eosin (HE) staining and wheat germ agglutinin (WGA) staining in hearts, along with quantitative analysis of cardiomyocyte areas. (n = 6 per group).** (S)** Heart weight (HW)-to-tibial length (TL) ratio in mice. (n = 6 per group).** (Q)** and** (T)** Representative images of Masson trichrome staining and Picrosirius Red staining in hearts and quantitative analysis of cardiac interstitial fibrosis as indicated by Sirus Picrosirius Red staining. (n = 6 per group).** (Q)** and** (U)** Representative immunohistochemical staining of GPX4 in hearts and quantitative analysis of GPX4 staining intensity. (n = 6 per group).** (Q)** and** (V)** Representative fluorescence images and quantification of dihydroethidium (DHE) staining in hearts. (n = 6 per group).** (W)** MDA levels in heart tissues. (n = 6 per group).** (X)** GSH/GSSG ratio in heart tissues. (n = 6 per group). Data are presented as Mean ± SEM. *p < 0.05, **p < 0.01, ***p < 0.001, ****p < 0.0001. For statistical analysis, one-way ANOVA with Tukey's test for multiple comparisons was used for **D-I**, **M-P**, and **R-X**; two-way ANOVA with Tukey's test for multiple comparisons was used for **L**. IHC, immunohistochemistry.

To investigate the role of ferroptosis in DIC, we utilized the DOX-treated HL-1 cardiomyocytes or adult mouse cardiomyocytes (AMCMs) model *in vitro* and the well-established chronic DIC mouse model *in vivo* 14, 17, 37. We conducted Western blot analysis to assess the protein levels of ferroptosis-related markers in DOX-stressed HL-1 cardiomyocytes and found that two molecules that inhibit ferroptosis, including glutathione peroxidase 4 (GPX4) and solute carrier family 7 member 11 (SLC7A11) were gradually decreased with increasing DOX exposure time or concentration (**Figure 1A**). In contrast, the levels of molecules that promote ferroptosis, such as acyl-CoA synthetase long-chain family member 4 (ACSL4) and transferrin receptor (TFR), were significantly elevated in a time- and concentration- dependent with DOX treatment (**Figure 1A**). Additionally, we examined the protein levels of antioxidative stress markers including superoxide dismutase 2 (SOD2), heme oxygenase 1 (HO-1), and nuclear factor erythroid 2-related factor 2 (NRF2), and observed a gradual decrease in these antioxidant molecules as increasing DOX exposure time and concentration (**Figure 1A**).

Next, we assessed iron and lipid peroxide levels, along with downstream markers of ferroptosis, in DOX-treated cardiomyocytes with or without co-treatment with the ferroptosis inhibitor, Fer-1 (a lipid peroxide scavenger) (**Figure 1B**). In DOX-stressed AMCMs, increases in intracellular iron and lipid peroxide levels were observed compared to the DMSO control group, as shown by FerroOrange staining and Liperfluo staining, respectively (**Figure 1C**). DOX-challenged AMCMs exhibited a decrease in mitochondrial membrane potential (MMP) and an increase in mitochondrial superoxide production, as demonstrated by JC-1 staining and MitoSOX staining, separately (**Figure 1C**), indicative of mitochondrial dysfunction. However, these deleterious effects induced by DOX stress were notably abrogated by Fer-1 treatment (**Figure 1C**). In addition, DOX insult led to mitochondrial iron overload (**Figure 1D**), increased mitochondrial lipid peroxide (**Figure 1E**), elevated levels of malondialdehyde (MDA) (**Figure 1F**), another end-product of lipid peroxidation, and a significantly reduced GSH/GSSG ratio (**Figure 1G**) in HL-1 cardiomyocytes. Nevertheless, treatment with Fer-1 markedly attenuated these ferroptotic phenotypes (**Figure 1D-G**). Unsurprisingly, DOX treatment significantly increased lactate dehydrogenase (LDH) release (**Figure 1H**), while lowering ATP production levels (**Figure 1I**) in cardiomyocytes. In contrast, these detrimental effects were significantly alleviated or abolished by Fer-1 treatment (**Figure 1H-I**).

To further evaluate the therapeutic relevance of ferroptosis inhibition in the chronic DIC mouse model, we intraperitoneally injected Fer-1 into mice one day before DOX administration and subsequently every day for 28 days (**Figure 1J**). Western blot analysis revealed that Fer-1 treatment effectively blocked the activation of ferroptosis pathway (**Figure 1K-L**) and reduced the protein levels of fibrotic markers and heart failure indicators, such as collagen type III alpha 1 chain (COL3A1), fibronectin (Fn), transforming growth factor beta 1 (TGFβ1), atrial natriuretic peptide (ANP), and b-type natriuretic peptide (BNP) (**Figure 1K-L**) in DOX-treated mouse hearts. Elevated levels of cardiac injury markers, including plasma cardiac troponin T (cTnT) (**Figure 1M**) and creatine kinase-myocardial band (CK-MB) (**Figure 1N**), along with significant decline in left ventricular systolic function (LVSF) (**Figure 1O-Q**) were observed in mice 1 week after the final dose of DOX. In contrast, Fer-1-treated mice exhibited preserved LVSF and significantly lowered levels of plasma cTnT and CK-MB (**Figure 1M-Q**). Next, cardiac atrophy and fibrosis, other phenotypes of cardiotoxicity, were histologically measured (**Figure 1Q**). DOX-treated mice displayed notable reductions in heart weight, heart size, and cardiomyocyte size (**Figure 1Q-S**), along with a marked increase in fibrotic area (**Figure 1Q and T**), whereas all these adverse effects were significantly prevented in Fer-1-treated mice (**Figure 1Q-T**). Furthermore, Fer-1 treatment inhibited DOX-induced GPX4 protein expression decreasing (**Figure 1Q and U**) and mitigated cardiac ROS production increasing (**Figure 1Q and V**), as evidenced by immunohistochemistry staining and immunofluorescence staining analyses, respectively. Additionally, Fer-1 treatment also prevented MDA levels elevation (**Figure 1W**) and GSH/GSSG ratio dropping (**Figure 1X**) in DOX-challenged mouse heart. Altogether, these data support the hypothesis that ferroptosis is significantly activated in DIC, and inhibiting ferroptosis effectively confers cardioprotection during DIC.

### DOX decreases AIG1 expression in cardiomyocytes and AIG1 knockout aggravates DOX-induced ferroptosis to deteriorate cardiotoxicity *in vivo*

**Figure 2:**
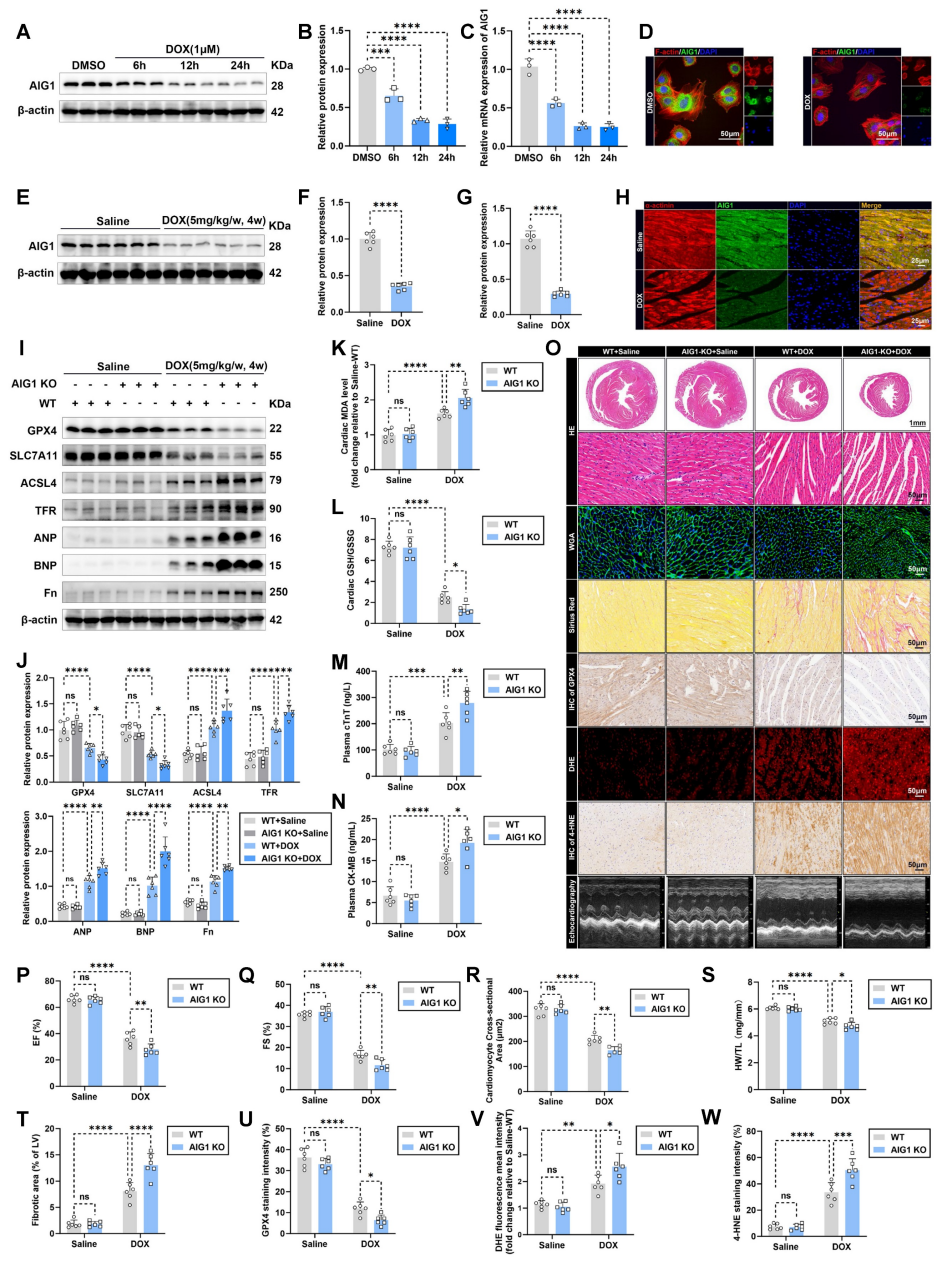
** DOX decreases AIG1 expression in cardiomyocytes and AIG1 knockout aggravates DOX-induced ferroptosis to deteriorate cardiotoxicity *in vivo*. (A)** and** (B)** Representative immunoblotting images and statistical analysis of AIG1 expression in DOX-challenged HL-1 cardiomyocytes. (n = 3 per group). **(C)** Relative mRNA levels of AIG1 in DOX-treated HL-1 cardiomyocytes quantified using RT-qPCR. (n = 3 per group). **(D)** Representative fluorescence images of AIG1 in HL-1 cardiomyocytes after DOX (1 μM, 24 h) treatment, detected by immunofluorescence staining. **(E)** and** (F)** Representative immunoblotting images and quantification of AIG1 expression in mouse heart lysates with or without DOX challenge. (n = 6 per group). **(G)** Relative mRNA levels of AIG1 in mouse heart lysates with or without DOX challenge quantified using RT-qPCR. (n = 6 per group). ** (H)** Representative fluorescence images of AIG1 in heart tissues from the chronic DIC mouse model, detected by immunofluorescence staining. **(I)** and** (J)** Representative immunoblot and statistical analysis of protein expression of ferroptosis markers, ANP, BNP, and fibronectin (Fn) in mouse heart lysates from wild type (WT) and AIG1 knockout (AIG KO) mice with or without DOX challenge. (n = 6 per group).** (K)** MDA levels in heart tissues. (n = 6 per group). **(L)** GSH/GSSG ratio in heart tissues. (n = 6 per group). **(M)** and** (N)** The levels of plasma cTnT and CK-MB in mice. (n = 6 per group). **(O)** through **(Q)** Representative M-mode echocardiographic image **(O)** and analysis of LVEF **(P)** and LVFS **(Q)** in WT and AIG1 KO mice with or without DOX challenge. (n = 6 per group). **(O)** and **(R)** Representative images of HE staining and WGA staining in hearts, along with quantitative analysis of cardiomyocyte areas. (n = 6 per group). **(S)** Heart weight (HW)-to-tibial length (TL) ratio in mice. (n = 6 per group). **(O)** and **(T)** Representative images of Picrosirius Red staining in hearts, along with quantitative analysis of cardiac interstitial fibrosis. (n = 6 per group). **(O)** and **(U)** Representative immunohistochemical staining of GPX4 in hearts, along with quantitative analysis of GPX4 staining intensity. (n = 6 per group).** (O)** and **(V)** Representative fluorescence images and quantification of DHE staining in hearts. (n = 6 per group). **(O)** and** (W)** Representative immunohistochemical staining of 4-hydroxynonenal (4-HNE) in hearts, along with quantitative analysis of 4-HNE staining intensity. (n = 6 per group). Data are presented as Mean ± SEM. *p < 0.05, **p < 0.01, ***p < 0.001, ****p < 0.0001. ns, no significance. For statistical analysis, one-way ANOVA with Tukey's test for multiple comparisons was used for **B-C**; unpaired student's t-test was used for **F-G**; two-way ANOVA with Tukey's test for multiple comparisons was used for **J-N** and **P-W**. DAPI, 4'6-diamidino-2-phenylindole; EF, ejection fraction; FS, fractional shortening.

To elucidate the potential involvement of AIG1 in DIC, we first detected the expression levels of AIG1 in DOX-stressed HL-1 cardiomyocytes. Both AIG1 protein (**Figure 2A-B**) and mRNA (**Figure 2C**) levels significantly decreased after 6 hours of DOX treatment, reaching their lowest levels after 24 hours of DOX exposure in HL-1 cardiomyocytes. We also analyzed the subcellular localization of AIG1 using immunofluorescence staining, which revealed that AIG1 showed a predominantly cytoplasmic distribution surrounding the nucleus (**Figure 2D**), likely associated with the ER, consistent with previous studies 32, 35, and this cytoplasmic localization was significantly reduced in HL-1 cardiomyocytes under DOX stress (**Figure 2D**). Considering the potential involvement of other cardiac cell types, such as cardiac fibroblasts (CFs) 38, 39 or endothelial cells (ECs) 40-42, in the pathological process of DIC, we searched for the Human Protein Atlas database (https://www.proteinatlas.org/) and found that cardiomyocytes (CMs) are the primary source of AIG1 in the hearts (**Figure S1A**). We isolated various types of cardiac cells, including CMs, CFs and ECs from adult male mice using our previously described methods 43. Our results demonstrated that AIG1 protein and mRNA levels were markedly higher in CMs compared to CFs or ECs (**Figure S1B-C**). Importantly, DOX treatment led to a significant downregulation of AIG1 protein and mRNA expressions in CMs (**Figure S1D-E**), while no noticeable changes were observed in AIG1 expression in CFs (**Figure S1F-G**) or ECs (**Figure S1H-I**). Consistent with our *in vitro* findings, Western blot analysis (**Figure 2E-F**), RT-qPCR (**Figure 2G**), and immunofluorescence staining (**Figure 2H**) confirmed a significant reduction in AIG1 expression in the hearts of DOX-treated male mice compared to saline-treated controls. Additionally, AIG1 expression was similarly suppressed in the hearts of DOX-treated female mice (**Figure S1J-K**). Interestingly, no significant differences in AIG1 protein or mRNA levels were observed between male and female mice, regardless of DOX challenge (**Figure S1J-K**), suggesting that the role of AIG1 in DIC is likely independent of sex, although its regulation may be influenced by androgen levels 25.

For ethical considerations regarding animal welfare, and based on the well-established chronic DIC mouse model 14, wild type (WT) and global AIG1 knockout (AIG1 KO) male mice (**Figure S2A**) therefore were used to further investigate the role of AIG1 in cardiac damage and cardiomyocyte ferroptosis under DOX challenge. The efficacy of AIG1 knockout in mouse hearts was validated by Western blot analysis (**Figure S2B**). As a result, global AIG1 deficiency significantly aggravated DOX-elicited cardiac ferroptosis activation and myocardial injury, as evidenced by decreased GPX4 and SLC7A11 protein levels, and increased ACSL4, TFR, ANP, BNP, and Fn protein levels (**Figure 2I-J**). DOX administration led to elevated cardiac MDA levels and a lowered GSH/GSSG ratio in heart tissues from WT mice, which was significantly exacerbated in AIG1 KO mice (**Figure 2K-L**). The increases in plasma cTNT and CK-MB levels following DOX treatment were also further worsened by AIG1 knockout (**Figure 2M-N**). Echocardiographic assessments (**Figure 2O**) indicated that DOX-stressed left ventricular systolic dysfunction was greatly deteriorated by AIG1 ablation, as evidenced by the decreased left ventricular ejection fraction (EF) and fractional shortening (FS) (**Figure 2P-Q**). Histological analysis (**Figure 2O**) showed that AIG1 KO mice displayed a drastic reduction in cardiomyocyte size (**Figure 2R**) and heart weight-to-tibial length (HW/TL) ratio (**Figure 2S**), along with a significant increase in myocardial fibrotic area (**Figure 2T**) compared to WT mice in the face of DOX insult. Additionally, cardiac GPX4 abundance was significantly reduced (**Figure 2O and U**), while the levels of ROS (**Figure 2O and V**) and 4-hydroxynonenal (4-HNE, a key biomarker of lipid peroxidation) (**Figure 2O and W**) were distinctly elevated in AIG1 KO mice subjected to DOX treatment compared to DOX-stressed WT mice.

To further confirm the precise role of AIG1 in the pathology of cardiomyocytes during DIC, we created a male murine model of cardiac-specific knockdown of AIG1 through intravenously injecting adeno-associated virus serotype 9 (AAV9) carrying shRNA for AIG1 (AAV9-shAIG1) or a negative control virus (AAV9-shNC) with the cardiac troponin T promoter, 3 weeks prior to DOX administration (**Figure S2C**). The efficiency of AIG1 knockdown in cardiac CMs of AAV9-shAIG1 mice was verified by Western blot analysis (**Figure S2D-J**). Predictably, AIG1 knockdown significantly exacerbated DOX-induced cardiomyocyte ferroptosis and myocardial damage (**Figure S3A-F**), consistent with observations in global AIG1 knockout models. AAV9-shAIG1 group displayed worsened cardiac systolic function and more severe ventricular pathological injury in comparison to AAV9-shNC group after DOX treatment (**Figure S3G-O**). Taken together, these findings strongly demonstrate that deletion of AIG1 aggravates DOX-induced cardiac ferroptosis and cardiotoxicity *in vivo*.

### AIG1 deficiency exacerbates DOX-induced cardiomyocyte ferroptosis *in vitro*

**Figure 3:**
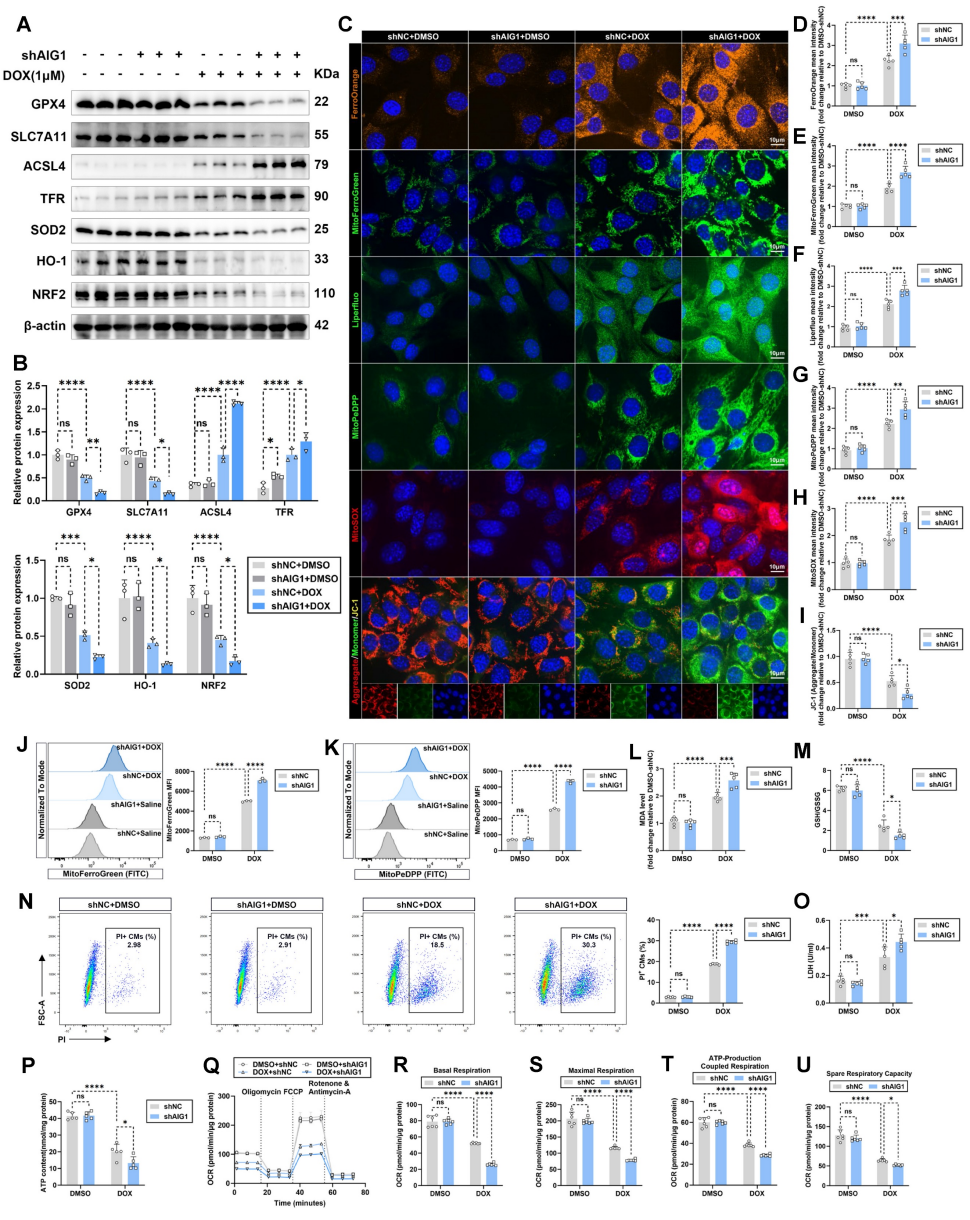
** AIG1 deficiency exacerbates DOX-induced cardiomyocyte ferroptosis *in vitro*. (A)** and** (B)** Representative immunoblot and statistical analysis of the protein expression of ferroptosis and oxidative stress markers in HL-1 cardiomyocytes with or without DOX (1 μM, 24 h) challenge after adenovirus transfection. (n = 3 per group). **(C)** Representative fluorescence images of FerroOrange staining, MitoFerroGreen staining, Liperfluo staining, MitoPeDPP staining, MitoSOX staining, and JC-1 staining in HL-1 cardiomyocytes with or without DOX (1 μM, 24 h) challenge after adenovirus transfection. Nuclei were counterstained with Hoechst 33342 (blue).** (D)** Quantification of intracellular iron level (FerroOrange staining) in HL-1 cardiomyocytes with indicated treatment. (n = 5 per group). **(E)** Quantification of intracellular lipid peroxide (Liperfluo staining) in HL-1 cardiomyocytes with indicated treatment. (n = 5 per group).** (F)** Quantification of mitochondrial iron level (MitoFerroGreen staining) in HL-1 cardiomyocytes with indicated treatment. (n = 5 per group). **(G)** Quantification of mitochondrial lipid peroxide (MitoPeDPP staining) in HL-1 cardiomyocytes with indicated treatment. (n = 5 per group). **(H)** Quantification of mitochondrial superoxide (MitoSOX staining) in HL-1 cardiomyocytes with indicated treatment. (n = 5 per group). **(I)** Quantification of mitochondrial membrane potential (JC-1 staining) in HL-1 cardiomyocytes with indicated treatment. (n = 5 per group).** (J)** Representative histogram showing mitochondrial iron level (MitoFerroGreen staining) in HL-1 cardiomyocytes with indicated treatment and quantitative analysis of MFI using flow cytometry. (n = 3 per group). **(K)** Representative histogram showing mitochondrial lipid peroxide (MitoPeDPP staining) in HL-1 cardiomyocytes with indicated treatment and quantitative analysis of the mean fluorescence intensities (MFI) using flow cytometry. (n = 3 per group). **(L)** MDA levels in HL-1 cardiomyocytes with or without DOX (1 μM, 24 h) challenge after adenovirus transfection. (n = 5 per group). **(M)** GSH/GSSG ratio in HL-1 cardiomyocytes with or without DOX (1 μM, 24 h) challenge after adenovirus transfection. (n = 5 per group). **(N)** The percentage of PI^+^ HL-1 cardiomyocytes was calculated using flow cytometry. (n = 5 per group). **(O)** LDH release in HL-1 cardiomyocytes with or without DOX (1 μM, 24 h) challenge after adenovirus transfection. (n = 5 per group). **(P)** ATP content in HL-1 cardiomyocytes with or without DOX (1 μM, 24 h) challenge after adenovirus transfection. (n = 5 per group). **(Q)** through **(U)** Real-time oxygen consumption rates (OCR) curves **(Q)** and quantification of basal respiration **(R)**, maximal respiration** (S)**, ATP-coupled respiration **(T)**, and spare respiration capacity** (U)** in HL-1 cardiomyocytes with or without DOX (1 μM, 24 h) challenge after adenovirus transfection. (n = 6 per group). Data are presented as Mean ± SEM. *p < 0.05, **p < 0.01, ***p < 0.001, ****p < 0.0001. ns, no significance. For statistical analysis, two-way ANOVA with Tukey's test for multiple comparisons was used for **B**, **D-P**, and **R-U**. CMs, cardiomyocytes; PI, propidium iodide.

To investigate the role of loss-of-function for AIG1 in cardiomyocyte ferroptosis upon DOX challenge *in vitro*, we knocked down AIG1 expression in HL-1 cardiomyocytes by transfecting adenoviruses carrying vectors encoding shRNA targeting AIG1 prior to DOX treatment (**Figure S4A-C**). The ferroptosis pathway and antioxidant stress markers were examined by Western blot analysis (**Figure 3A-B**). In keeping with our mouse data, AIG1 silencing significantly aggravated cardiomyocyte ferroptosis and oxidative damage, as indicated by a reduction in protein levels of GPX4, SLC7A11, SOD2, HO-1, and NRF2, accompanied by increases in protein abundance of ACSL4 and TFR (**Figure 3A-B**). Moreover, intracellular iron (**Figure 3C-D**) and lipid peroxide levels (**Figure 3C and E**) were further elevated due to AIG1 knockdown in DOX-treated HL-1 cardiomyocytes, as shown by FerroOrange and Liperfluo staining, respectively (**Figure 3C**). The accumulation of iron, lipid peroxide, superoxide in mitochondria, and MMP were also assessed.

We found that AIG1 inhibition evoked higher levels of mitochondrial iron (**Figure 3C and F**) and lipid peroxide (**Figure 3C and G**), mitochondrial superoxide overproduction (**Figure 3C and H**), and a more severe loss of MMP (**Figure 3C and I**), as demonstrated by MitoFerroGreen, MitoPeDPP, MitoSOX, and JC-1 staining, respectively (**Figure 3C**). Cytometry analysis similarly revealed that mitochondrial iron and lipid peroxide overload following AIG1 knockdown in DOX-stressed HL-1 cardiomyocytes (**Figure 3J-K**). DOX treatment resulted in increased MDA levels (**Figure 3L**) and dropped GSH/GSSG ratio (**Figure 3M**) in HL-1 cardiomyocytes, both of which were notably exacerbated by AIG1 knockdown. Additionally, AIG1 knockdown led to a higher proportion of PI-positive cardiomyocytes (**Figure 3N**), elevated LDH release (**Figure 3O**), and reduced ATP production (**Figure 3P**), indicating worsened cardiomyocyte injury in response to DOX stress. Mitochondrial respiratory function was assessed by examining mitochondrial oxygen consumption rate (OCR) (**Figure 3Q**). A significant reduction in basal (**Figure 3R**), maximal (**Figure 3S**), ATP-production coupled respiration (**Figure 3T**), and spare respiratory capacity (**Figure 3U**) was observed in HL-1 cardiomyocytes upon DOX challenge. This reduction was further exacerbated by AIG1 deficiency (**Figure 3R-U**).

To further confirm the effects of AIG1 knockout on ferroptosis and mitochondrial function in primary cardiomyocytes, AMCMs were isolated from WT and AIG1 KO male mice and subjected to DOX treatment (**Figure S4D**). We consistently observed that AIG1 deletion dramatically exacerbated DOX-induced ferroptosis and mitochondria dysfunction, as shown by increased intracellular iron deposition (**Figure S4E-F**), overwhelmed lipid peroxidation (**Figure S4E and G**), elevated mitochondrial superoxide production (**Figure S4E and H**), collapsed MMP (**Figure S4E and I**), impaired cell viability (**Figure S4J**), and reduced ATP synthesis (**Figure S4K**) in AMCMs. Furthermore, the mechanical properties of AMCMs were evaluated after DOX treatment through contractile function measurements in single AMCM. DOX-induced cardiomyocyte mechanical defects were markedly deteriorated in AMCMs with AIG1 knockout, as evidenced by reduced resting cell length (**Figure S4L**), peak shortening (**Figure S4M**), and maximal velocity of shortening/relengthening (**Figure S4N-O**).

Given that other forms of cardiomyocyte death may also critically contribute to the progression of DIC 24, we next investigated whether AIG1 was primarily involved in regulating ferroptosis, rather than other cell death modalities. We found that AIG1 knockdown reduced cell viability and enhanced cell susceptibility to DOX stress and ferroptosis inducers (erastin and RSL3), accompanied by elevated LDH release and lowered ATP production (**Figure S5A-C**). Notably, these anomalies evoked by AIG1 inhibition were significantly reversed by the ferroptosis inhibitors Fer-1 or DFO, but not by the apoptosis inhibitor Z-VAD, necroptosis inhibitor Nec-1, autophagy inhibitor 3-MA, or pyroptosis inhibitor MCC950 (**Figure S5A-C**) in DOX-stressed HL-1 cardiomyocytes. Moreover, we also examined various markers expressions of other cell death pathways in DOX-treated HL-1 cardiomyocytes using Western blot and found AIG1 knockdown had no effect on apoptosis (**Figure S5D-E**), necroptosis (**Figure S5F-G**), autophagy (**Figure S5H-I**), and pyroptosis (**Figure S5J-K**). Collectively, these results underscore an obligatory role for AIG1 in DOX-evoked ferroptosis and cardiomyocyte injury.

### AIG1 overexpression protects against DOX-induced cardiotoxicity and attenuates cardiomyocyte ferroptosis *in vitro* and *in vivo*

**Figure 4:**
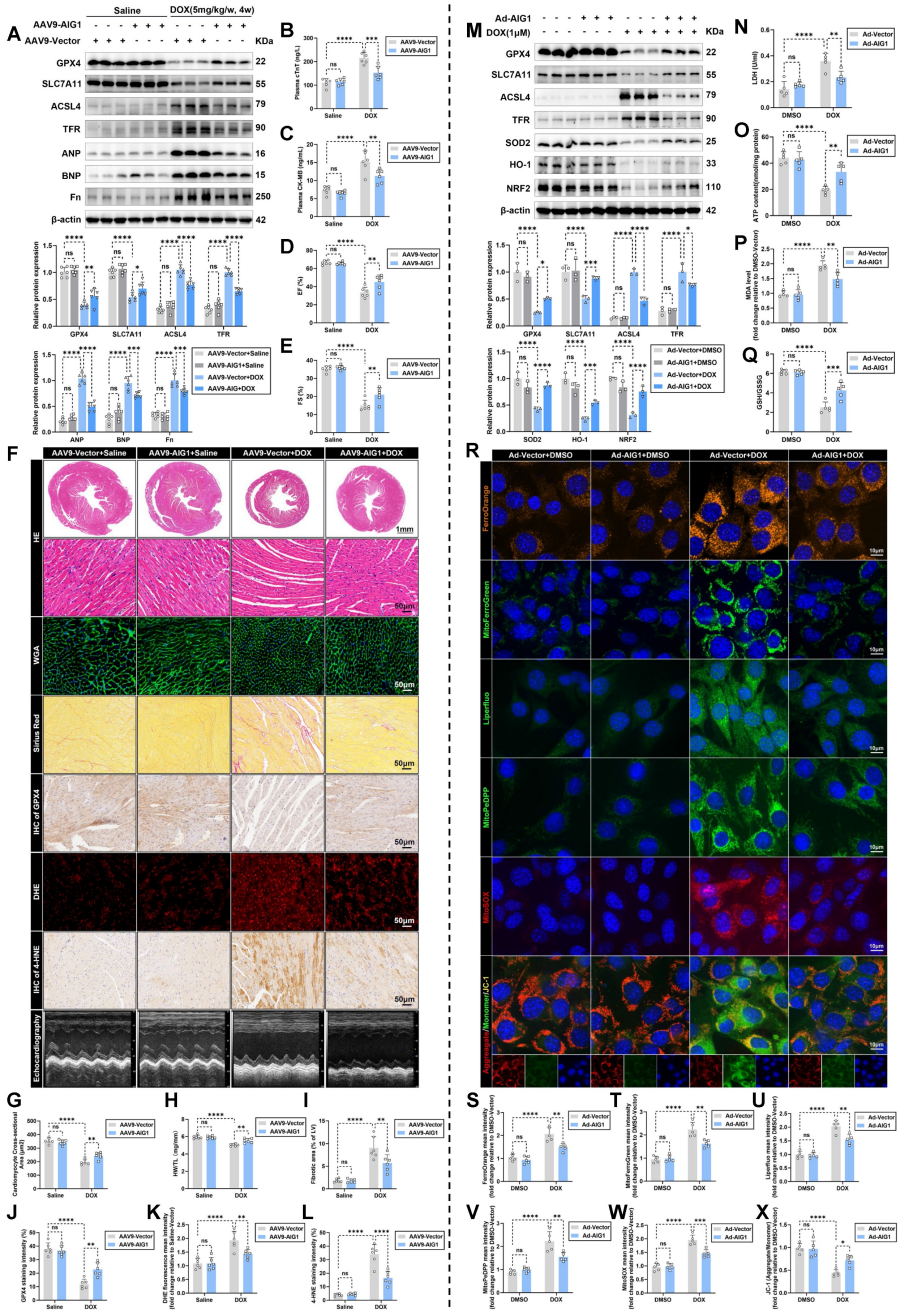
** AIG1 overexpression protects against DOX-induced cardiotoxicity and attenuates cardiomyocyte ferroptosis *in vivo* and *in vitro*. (A)** Representative immunoblot and statistical analysis of protein expressions of ferroptosis markers, ANP, BNP, and fibronectin (Fn) in mouse heart lysates from AAV9-Vector and AAV9-AIG1 mice with or without DOX challenge. (n = 6 per group).** (B)** and **(C)** The levels of plasma cTnT and CK-MB in mice. (n = 6 per group). **(D)** through** (F)** Quantification of LVEF **(D)** and LVFS **(E)** along with representative M-mode echocardiographic images** (F)** in AAV9-Vector and AAV9-AIG1 mice with or without DOX challenge. (n = 6 per group). **(F)** and **(G)** Representative images of HE staining and WGA staining in hearts and quantitative analysis of cardiomyocyte areas. (n = 6 per group). **(H)** Heart weight (HW)-to-tibial length (TL) ratio in mice. (n = 6 per group). **(F)** and **(I)** Representative images of Picrosirius Red staining in hearts and quantitative analysis of cardiac interstitial fibrosis. (n = 6 per group). **(F)** and **(J)** Representative immunohistochemical staining of GPX4 in hearts and quantitative analysis of GPX4 staining intensity. (n = 6 per group). **(F)** and **(K)** Representative fluorescence images and quantification of DHE staining in hearts. (n = 6 per group). **(F)** and** (L)** Representative immunohistochemical staining of 4-HNE in hearts and quantitative analysis of 4-HNE staining intensity. (n = 6 per group). **(M)** Representative immunoblot and statistical analysis of protein expression of ferroptosis and oxidative stress markers in HL-1 cardiomyocytes with or without DOX (1 μM, 24 h) challenge after adenovirus transfection. (n = 3 per group). **(N)** LDH release in HL-1 cardiomyocytes with or without DOX (1 μM, 24 h) challenge after adenovirus transfection. (n = 5 per group).** (O)** ATP content in HL-1 cardiomyocytes with or without DOX (1 μM, 24 h) challenge after adenovirus transfection. (n = 5 per group). **(P)** MDA levels in HL-1 cardiomyocytes with or without DOX (1 μM, 24 h) challenge after adenovirus transfection. (n = 5 per group). **(Q)** GSH/GSSG ratio in HL-1 cardiomyocytes with or without DOX (1 μM, 24 h) challenge after adenovirus transfection. (n = 5 per group). **(R)** Representative fluorescence images of FerroOrange staining, MitoFerroGreen staining, Liperfluo staining, MitoPeDPP staining, MitoSOX staining, and JC-1 staining in HL-1 cardiomyocytes with or without DOX (1 μM, 24 h) challenge after adenovirus transfection. Nuclei were counterstained with Hoechst 33342 (blue).** (S)** Quantification of intracellular iron level (FerroOrange staining) in HL-1 cardiomyocytes with indicated treatment. (n = 5 per group).** (T)** Quantification of mitochondrial iron level (MitoFerroGreen staining) in HL-1 cardiomyocytes with indicated treatment. (n = 5 per group).** (U)** Quantification of intracellular lipid peroxide (Liperfluo staining) in HL-1 cardiomyocytes with indicated treatment. (n = 5 per group). **(V)** Quantification of mitochondrial lipid peroxide (MitoPeDPP staining) in HL-1 cardiomyocytes with indicated treatment. (n = 5 per group). **(W)** Quantification of mitochondrial superoxide (MitoSOX staining) in HL-1 cardiomyocytes with indicated treatment. (n = 5 per group). **(X)** Quantification of mitochondrial membrane potential (JC-1 staining) in HL-1 cardiomyocytes with indicated treatment. (n = 5 per group). Data are presented as Mean ± SEM. *p < 0.05, **p < 0.01, ***p < 0.001, ****p < 0.0001. ns, no significance. For statistical analysis, two-way ANOVA with Tukey's test for multiple comparisons was used for **A-E**, **G-Q**, and **S-X**. AAV9, adeno-associated virus serotype 9.

Meanwhile, heart-restricted AIG1 overexpressed male mice were similarly generated to further validate the specific role of AIG1 in DOX-induced cardiomyocyte ferroptosis and cardiotoxicity by injecting C57BL/6 male mice with AAV9 via the tail vein (**Figure S6A**). The AAV encoding mouse AIG1 cDNA (AAV9-AIG1) or an empty control vector (AAV9-Vector) was driven by the cardiac troponin T promoter. 3 weeks following the AAV9 injection, the mice were exposed to DOX challenge and subsequently monitored as illustrated (**Figure S6A**). Significant myocardial-specific overexpression of AIG1 was confirmed in cardiac CMs of AAV9-AIG1 mice (**Figure S6B-H**). Our data revealed that the cardiac ferroptosis pathway was significantly suppressed by AIG1 overexpression in the myocardium (**Figure 4A**), accompanied by lowered levels of myocardial injury markers, including cTnT and CK-MB, in mouse plasma after DOX stress (**Figure 4B-C**). Additionally, echocardiographic measurements noted that myocardial AIG1 overexpressed mice exhibited improved LVSF, manifested by preserved EF (**Figure 4D and F**) and FS (**Figure 4E-F**). Histological evaluations also indicated that DOX-mediated cardiac atrophy and fibrosis were significantly mitigated by AIG1 overexpression (**Figure 4F**), as evidenced by an increased cardiomyocyte size (**Figure 4G**) and a higher heart weight-to-tibial length (HW/TL) ratio (**Figure 4H**), along with decreased myocardial interstitial fibrosis (**Figure 4I**). Moreover, reductions in cardiac GPX4 and 4-HNE abundance, along with ROS overproduction in DOX-treated hearts, were dramatically prevented by AIG1 overexpression (**Figure 4J-L**). Thus, these results support the cardioprotective properties of cardiac AIG1 overexpression via AAV9 delivery during DIC.

Likewise, we also investigated whether AIG1 overexpression, achieved through adenoviruses delivery (**Figure S7A-C**), would alleviate ferroptosis in HL-1 cardiomyocytes under DOX challenge. Predictably, AIG1 overexpression remarkably ameliorated DOX-induced cardiomyocyte ferroptosis and oxidative damage, as evidenced by Western blot analysis (**Figure 4M**). AIG1 overexpression improved cell viability, enhanced cell survival, reduced LDH release and mitigated the decline in ATP production triggered by DOX (**Figure 4N-O and S7D-E**), or the ferroptosis inducers erastin and RSL3 (**Figure S7F-H**). The increased MDA levels and reduced GSH/GSSG ratio in response to DOX insult were essentially nullified in AIG1 overexpressed HL-1 cardiomyocytes (**Figure 4P-Q**). Additionally, DOX-provoked increases in both intracellular and mitochondrial iron accumulation and lipid peroxidation, mitochondrial superoxide overproduction, and decreased MMP were effectively countered by AIG1 overexpression (**Figure 4R-X**). Furthermore, Western blot analysis also indicated that AIG1 overexpression did not affect other cardiomyocyte death modalities after DOX stress (**Figure S7I-P**), further emphasizing the uniqueness of AIG1 in regulating cardiomyocyte ferroptosis in DIC.

Overall, the above findings highlight the essential role of AIG1 in alleviating DOX-induced cardiomyocyte ferroptosis and cardiotoxicity.

### AIG1 promotes ubiquitination-mediated p53 degradation in cardiomyocytes against DOX-induced ferroptosis by interacting with Pirh2

**Figure 5:**
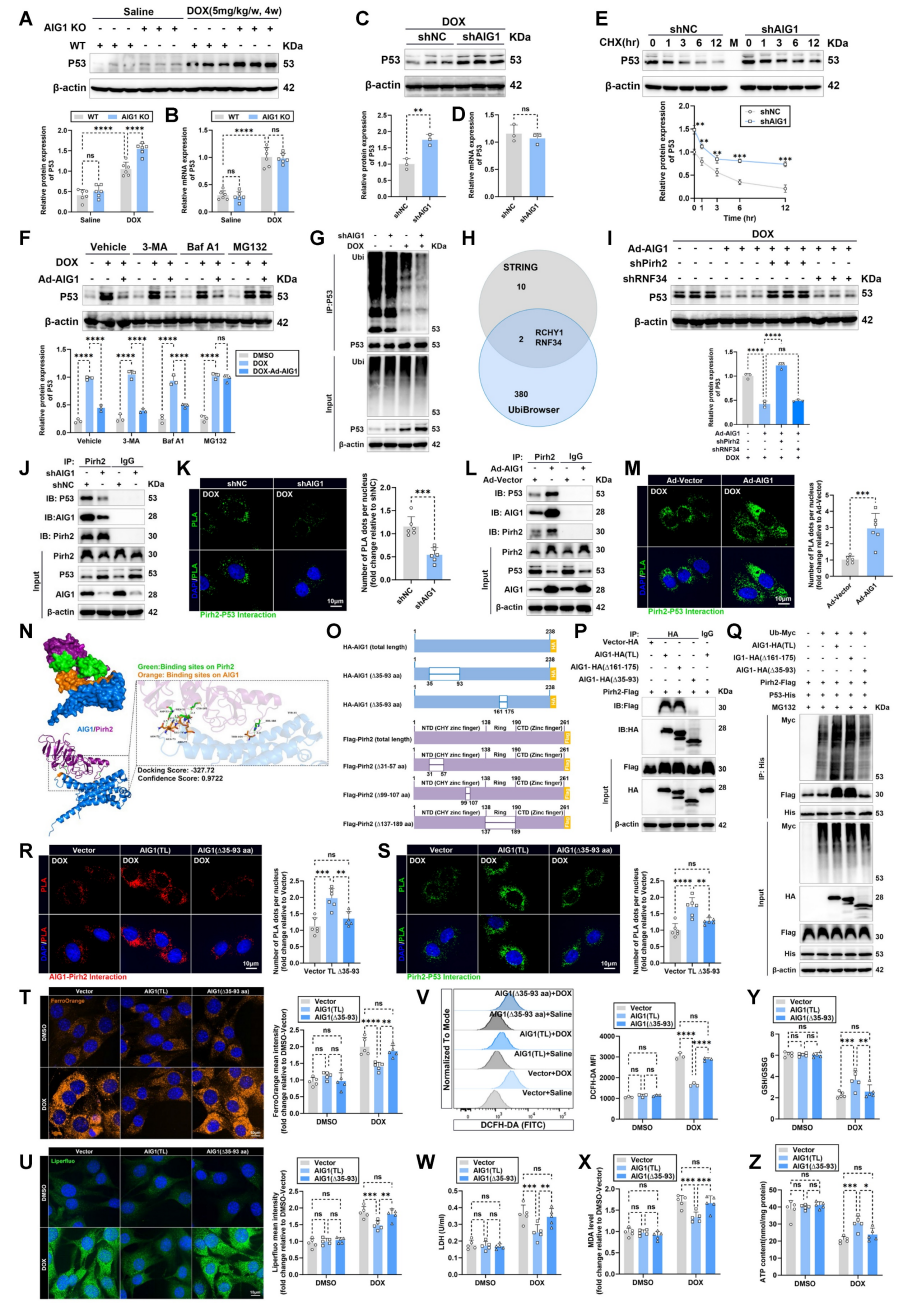
** AIG1 promotes ubiquitination-mediated p53 degradation in cardiomyocytes against DOX-induced ferroptosis by interacting with Pirh2. (A)** Representative immunoblot and statistical analysis of p53 protein expression in mouse heart lysates from WT and AIG1 KO mice with or without DOX challenge. (n = 6 per group). **(B)** Relative mRNA levels of p53 in mouse heart lysates from WT and AIG1 KO mice with or without DOX challenge quantified using RT-qPCR. (n = 6 per group). **(C)** Representative immunoblot images and statistical analysis of p53 protein expression in DOX-treated HL-1 cardiomyocytes with or without AIG1 knockdown. (n = 3 per group). **(D)** Relative mRNA levels of p53 in DOX-treated HL-1 cardiomyocytes with or without AIG1 knockdown quantified using RT-qPCR. (n = 3 per group). **(E)** Representative immunoblot images and quantification of p53 protein level in cycloheximide (CHX) chase assay in HL-1 cardiomyocytes with or without AIG1 knockdown under DOX stress. (n = 3 per group). Cells were treated with DOX (1 μM) for 24 h before the addition of CHX (10 μM) for time-series analysis.** (F)** Representative immunoblot images and statistical analysis of p53 protein expression in HL-1 cardiomyocytes with indicated treatment. Cardiomyocytes with or without AIG1 overexpression were treated with MG132 (10 μM) for 18 h, 3-MA (5 mM) for 18 h or Baf A1 (50 nM) for 4 h, prior to DOX challenge for 24 h. (n = 3 per group). **(G)** Ubiquitination assays were performed to determine the ubiquitination of endogenous p53 in HL-1 cardiomyocytes with indicated treatment.** (H)** Venn diagram showing the overlap of potential AIG1-interacting partners from STRING database and predicted ubiquitin ligases of p53 based on the UbiBrowser 2.0 database. The candidate ubiquitin ligases of p53 recruited by AIG1 are Pirh2 and RNF34. **(I)** Representative immunoblot images and statistical analysis of p53 protein expression in DOX-stressed HL-1 cardiomyocytes with indicated treatment. (n = 3 per group). **(J)** Representative immunoblotting images following Co-IP using anti-Pirh2 in HL-1 cardiomyocytes with or without AIG1 knockdown under DOX (1 μM, 24 h) stress. **(K)** Representative images and quantification of PLA analysis showing the interaction of Pirh2 with p53 in DOX-stressed HL-1 cardiomyocytes with AIG1 knockdown. (n = 6 per group). **(L)** Representative immunoblotting images following Co-IP using anti-Pirh2 in HL-1 cardiomyocytes with or without AIG1 overexpression under DOX (1 μM, 24 h) stress. **(M)** Representative images and quantification of PLA analysis showing the interaction of Pirh2 with p53 in DOX-stressed HL-1 cardiomyocytes with AIG1 overexpression. (n = 6 per group). **(N)** Structure-based protein interaction interface analysis between AIG1 (purple) and Pirh2 (blue) proteins with interactive residues predicted. **(O)** and **(P)** Graphs illustrating construction of total-length (TL) and truncated mutants of AIG1 and Pirh2 vectors, and representative immunoblotting images from Co-IP assays using an anti-HA tag in co-transfected HEK293T cells. **(Q)** Representative immunoblotting images demonstrating the effects of the indicated AIG1 mutants (Δ35-93, Δ161-175) on Pirh2-mediated exogenous p53 ubiquitination and its protein level in MG132-treated HEK293T cells co-transfected with the indicated constructs. **(R)** and **(S)** Representative images and quantification of PLA analysis showing the interaction of AIG1 with Pirh2 and Pirh2 with p53 in DOX-treated HL-1 cardiomyocytes transfected with Vector, AIG1 (TL)-, or AIG1 (Δ35-93)-overexpressed adenovirus. (n = 6 per group). **(T)** and **(U)** Representative fluorescence images and quantification of FerroOrange staining and Liperfluo staining in HL-1 cardiomyocytes transfected with Vector, AIG1 (TL)-, or AIG1 (Δ35-93)-overexpressed adenovirus, with or without DOX (1 μM, 24 h) challenge. Nuclei were counterstained with Hoechst 33342 (blue). (n = 5 per group).** (V)** Representative histogram showing intracellular ROS level (DCFH-DA staining) in HL-1 cardiomyocytes transfected with Vector, AIG1 (TL)-, or AIG1 (Δ35-93)-overexpressed adenovirus, with or without DOX (1 μM, 24 h) challenge, along with quantitative analysis of the mean fluorescence intensities (MFI) using flow cytometry. (n = 3 per group).** (W)** through **(Z)** LDH release, MDA levels, GSH/GSSG ratio, and ATP content in HL-1 cardiomyocytes transfected with Vector, AIG1 (TL)-, or AIG1 (Δ35-93)-overexpressed adenovirus, with or without DOX (1 μM, 24 h) challenge. (n = 5 per group). Data are presented as Mean ± SEM. *p < 0.05, **p < 0.01, ***p < 0.001, ****p < 0.0001. ns, no significance. For statistical analysis, two-way ANOVA with Tukey's test for multiple comparisons was used for **A-B**, **F** and **T-Z**; unpaired student's t-test was used for **C-E**, **K** and **M**; one-way ANOVA with Tukey's test for multiple comparisons was used for** I** and **R-S**. PLA, proximity ligation assay.

Next, we asked how AIG1 participated in modulating cardiomyocyte ferroptosis during DIC. Accumulating evidence suggests that p53 serves as a central regulator of the ferroptosis network 22, 44. p53 activation triggers cardiomyocyte ferroptosis both through the transcriptional and post-translational mechanism during DIC 17, 23, 45, 46. Interestingly, previous study demonstrated that AIG1 expression reduced p53 reporter gene activity 32. Thus, we speculated that AIG1 may critically regulate DOX-induced cardiomyocyte ferroptosis via p53 signaling. To verify this hypothesis, we first examined p53 expression in hearts following DOX challenge and observed a significant upregulation of both p53 protein and mRNA levels after DOX stimulation compared to the saline control (**Figure 5A-B and S8A**). Notably, AIG1 knockout further increased p53 protein level without altering its mRNA level (**Figure 5A-B and S8A**). Similarly, AIG1 knockdown markedly upregulated p53 protein expression in HL-1 cardiomyocytes under DOX challenge, whereas p53 mRNA level remained unaffected (**Figure 5C-D and S8B**). Conversely, AIG1 overexpression dramatically suppressed p53 protein expression (**Figure S8C-D and F**). However, no significant differences were observed for the mRNA level of p53 in mouse hearts or HL-1 cardiomyocytes with AIG1 overexpression (**Figure S8E and G**). These findings suggest that a potential role of AIG1 in regulating p53 at the post-translational level, possibly through protein stabilization or degradation pathway. In fact, a cycloheximide (CHX) chase assay conducted in HL-1 cardiomyocytes revealed that AIG1 silencing significantly decelerated p53 protein degradation (**Figure 5E**), whereas AIG1 overexpression evidently accelerated its degradation (**Figure S8H**).

There are two major quality control systems contributing to protein degradation in eukaryotic cells, namely the ubiquitin-proteasome and autolysosome pathways 47. To further determine degradation pathway of p53 mediated by AIG1, different inhibitors of protein degradation, including the autophagy inhibitor 3-methyladenine (3-MA), the autolysosome inhibitor bafilomycin A1 (Baf A1), and the proteasome inhibitor MG132 were therefore employed. Our results showed that protein level of p53 was reduced in response to AIG1 overexpression, an effect reversed by MG132 treatment but unaffected by 3-MA or Baf A1 under DOX stress (**Figure 5F**).

Additionally, Co-immunoprecipitation (Co-IP) results confirmed that p53 ubiquitination was inhibited in AIG1 knockdown HL-1 cardiomyocytes (**Figure 5G**) or in AIG1 KO mouse hearts (**Figure S8I**) upon DOX stress. These results suggest that AIG1-mediate p53 degradation is likely regulated through a ubiquitin-proteasome mechanism.

Next, we explore underlying mechanisms of p53 ubiquitination mediated by AIG1 in DOX-stressed cardiomyocytes. Prior studies demonstrated that multiple E3 ubiquitin ligases play a significant role in regulating the ubiquitination and subsequent proteasomal degradation of p53 in cardiomyocytes during DIC 23, 45, 48, 49. To investigate possible E3 ligase specifically recruited by AIG1, an integrated analysis of 10 potential AIG1-interacting partners based on the STRING database and 380 predicted ubiquitin ligases of p53 from the UbiBrowser 2.0 database revealed that Pirh2 or RNF34 is the most likely candidate to regulate the ubiquitination and degradation of p53 among many other AIG1 binding partners (**Figure 5H**). We assessed the expression of Pirh2 and RNF34 in cardiomyocytes following DOX treatment and observed that DOX stress significantly downregulated Pirh2 expressions *in vitro* and *in vivo,* whereas RNF34 expression remained unchanged after DOX challenge (**Figure S8J-K**). Importantly, we found that knockdown of Pirh2, but not RNF34, reversed the AIG1 overexpression-mediated downregulation (**Figure 5I**) and ubiquitination (**Figure S8L**) of p53 protein in DOX-stressed cardiomyocytes, suggesting that Pirh2 may serve as a critical downstream mediator responsible for AIG1-induced degradation of p53 in cardiomyocytes during DOX stress. Additionally, immunofluorescence staining in HL-1 cardiomyocytes revealed a strong interaction between AIG1 and Pirh2, with a Pearson's correlation coefficient of 0.83 (**Figure S8M**). Co-IP results similarly indicated that endogenous AIG1 strongly interacted with Pirh2 in cardiomyocytes under steady-state conditions, which was found to be reduced following DOX treatment (**Figure S8N**), whereas DOX treatment had no effects on the interaction between AIG1 and RNF34 (**Figure S8N**). Consistent with the Co-IP assay findings, the levels of AIG1-Pirh2 complexes were significantly reduced in response to DOX stress, as confirmed by the proximity ligation assay (PLA) (**Figure S8O-P**). Moreover, the levels of Pirh2-p53 complexes were also markedly reduced in HL-1 cardiomyocytes upon DOX challenge compared with the DMSO-treated control (**Figure S8Q**). Interestingly, AIG1 knockdown significantly downregulated Pirh2 protein expression and decreased Pirh2-p53 interaction in DOX-stressed HL-1 cardiomyocytes (**Figure 5J-K**), whereas AIG1 overexpression indeed upregulated Pirh2 protein expression and markedly enhanced the interaction of Pirh2 and p53 (**Figure 5L-M**), as demonstrated by Co-IP and PLA assays. Taken together, these results provide strong evidence that AIG1 can actively recruit E3 ubiquitin ligase Pirh2 to facilitate ubiquitination-dependent degradation of p53 protein in DOX-stressed cardiomyocytes.

To further identify the molecular domains responsible for AIG1-Pirh2 interaction and their effects on p53 ubiquitination, molecular docking was performed to visualize the optimal binding conformation of AIG1-Pirh2 and predict potential interacting sites (**Figure 5N**). Accordingly, truncated mutants of AIG1 and Pirh2 were constructed for Co-IP assays in the HEK293T cell line (**Figure 5O**). The results showed that Pirh2 interacted with full-length AIG1 and the AIG1 Δ161-175 aa mutant, but not with the AIG1 Δ35-93 aa mutant when co-expressed in HEK293T cells, indicating the essential role of the 35-93 aa domain of AIG1 in its interaction with Pirh2 (**Figure 5P**). Importantly, the deletion of the 35-93 aa domain of AIG1 resulted in significant reductions in Pirh2-p53 interaction and p53 ubiquitination (**Figure 5Q**). On the other hand, AIG1 interacted with full-length Pirh2, the Pirh2 Δ99-107 aa mutant, and the Pirh2 Δ137-189 aa mutant but not with the Pirh2 Δ31-57 aa mutant, suggesting an obligatory role for the 31-57 aa domain of Pirh2 in its interaction with AIG1 (**Figure S8R**). Indeed, the deletion of the 31-57 aa domain of Pirh2 also notably inhibited p53 ubiquitination by disrupting the Pirh2-p53 interaction (**Figure S8S**).

Furthermore, to evaluate the effects of the identified AIG1-Pirh2 interacting sites on DOX-induced ferroptosis in cardiomyocytes, adenoviruses containing AIG1 (total length, TL), AIG1 (Δ35-93), Pirh2 (total length, TL), or Pirh2 (Δ31-57) were constructed and delivered into HL-1 cardiomyocytes, which were then challenged with or without DOX. Similarly, PLA results confirmed that the critical 35-93 aa domain of AIG1 and the 31-57 aa domain of Pirh2 were responsible for the AIG1-Pirh2 interaction (**Figure 5R and S8T**) and affected the Pirh2-p53 binding (**Figure 5S and S8U**) in DOX-treated HL-1 cardiomyocytes. In addition, overexpression of AIG1 or Pirh2 significantly attenuated ferroptosis in DOX-stressed cardiomyocytes, as evidenced by the reduction in intracellular iron accumulation (**Figure 5T and S8V**), lipid peroxide (**Figure 5U and S8W**), ROS production (**Figure 5V and S8X**), LDH release (**Figure 5W**), and MDA levels (**Figure 5X and S8Y**), along with increases in the GSH/GSSG ratio (**Figure 5Y and S8Z**) and ATP synthesis (**Figure 5Z**). In contrast, deletion of AIG1 (aa 35-93) or Pirh2 (aa 31-57) failed to duplicate the protective effects of AIG1 (TL) or Pirh2 (TL) (**Figure 5T-Z and S8V-Z**). In summary, these results suggest that the specific AIG1-Pirh2 interaction plays a critical role in alleviating DOX-induced ferroptosis in cardiomyocytes by hindering ubiquitination-mediated p53 degradation.

### Pharmacological activation of p53 or cardiac-specific knockdown of Pirh2 aggravates DOX-induced ferroptosis and cardiotoxicity, and dampens AIG1 overexpression-mediated cardioprotection

**Figure 6:**
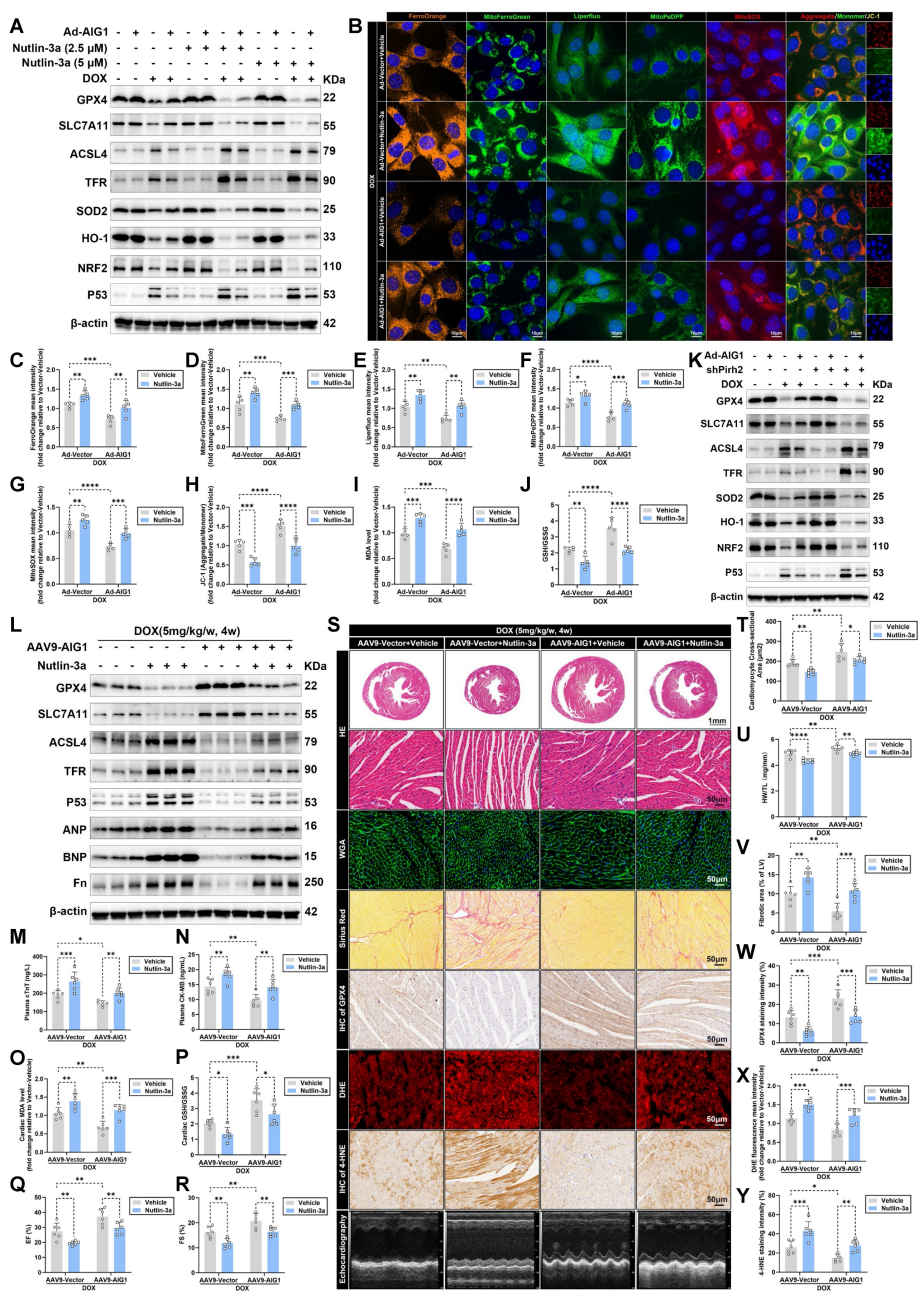
** Pharmacological activation of p53 aggravates DOX-induced ferroptosis and cardiotoxicity, and dampens AIG1 overexpression-mediated cardioprotection. (A)** through** (J)** HL-1 cardiomyocytes were exposed to DOX (1 μM, 24 h) in the absence or presence of AIG1 overexpression or Nutlin-3a (2.5 μM or 5 μM, 24 h). **(A)** Representative immunoblotting images of protein expression of ferroptosis and oxidative stress markers in HL-1 cardiomyocytes under various treatment settings. (n = 3 per group). **(B)** Representative fluorescence images of FerroOrange staining, MitoFerroGreen staining, Liperfluo staining, MitoPeDPP staining, MitoSOX staining, and JC-1 staining in DOX-treated HL-1 cardiomyocytes in the absence or presence of AIG1 overexpression or Nutlin-3a (5 μM, 24 h). Nuclei were counterstained with Hoechst 33342 (blue). **(C)** Quantification of intracellular iron level (FerroOrange staining) in HL-1 cardiomyocytes with indicated treatment. (n = 5 per group).** (D)** Quantification of mitochondrial iron level (MitoFerroGreen staining) in HL-1 cardiomyocytes with indicated treatment. (n = 5 per group). **(E)** Quantification of intracellular lipid peroxide (Liperfluo staining) in HL-1 cardiomyocytes with indicated treatment. (n = 5 per group). **(F)** Quantification of mitochondrial lipid peroxide (MitoPeDPP staining) in HL-1 cardiomyocytes with indicated treatment. (n = 5 per group). **(G)** Quantification of mitochondrial superoxide (MitoSOX staining) in HL-1 cardiomyocytes with indicated treatment. (n = 5 per group). **(H)** Quantification of mitochondrial membrane potential (JC-1 staining) in HL-1 cardiomyocytes with indicated treatment. (n = 5 per group). **(I)** and **(J)** MDA levels and GSH/GSSG ratio in HL-1 cardiomyocytes with indicated treatment. (n = 5 per group). **(K)** Representative immunoblotting images of protein expression of ferroptosis and oxidative stress markers in HL-1 cardiomyocytes treated with or without DOX (1 μM, 24 h) in the absence or presence of AIG1 overexpression or Pirh2 knockdown. (n = 3 per group). **(L)** through** (Y)** AAV9-Vector and AAV9-AIG1 mice were intraperitoneally (i.p.) injected with Nutlin-3a (20 mg/kg/day) throughout the DOX treatment period. (n = 6 per group). **(L)** Heart tissues samples were analyzed by Western blots analysis. **(M)** and** (N)** Plasma levels of cTnT and CK-MB in mice.** (O)** and** (P)** MDA levels and GSH/GSSG ratio in heart tissues.** (Q)** through** (S)** Quantification of LVEF **(Q)** and LVFS **(R)** shown with representative M-mode images **(S)** from transthoracic echocardiography. **(S)** and** (T)** Representative HE staining and WGA staining of hearts and quantitative analysis of cardiomyocyte areas. **(U)** Heart weight (HW)-to-tibial length (TL) ratio in mice.** (S)** and** (V)** Representative Picrosirius Red staining images of hearts and quantitative analysis of cardiac interstitial fibrosis.** (S)** and** (W)** Representative immunohistochemical staining of GPX4 in hearts and quantitative analysis of GPX4 staining intensity. **(S)** and** (X)** Representative fluorescence images and quantification of DHE staining in hearts. **(S)** and** (Y)** Representative immunohistochemical staining of 4-HNE in hearts and quantitative analysis of 4-HNE staining intensity. Data are presented as Mean ± SEM. *p < 0.05, **p < 0.01, ***p < 0.001, ****p < 0.0001. For statistical analysis, two-way ANOVA with Tukey's test for multiple comparisons was used for **C-J**, **M-R**, and **T-Y**. Ad, adenovirus.

To determine whether downstream p53 is responsible for AIG1-Pirh2 signaling in driving cardiomyocyte ferroptosis during DIC, p53 activation was induced by Nutlin-3a (a p53 activator) treatment in HL-1 cardiomyocytes. Western blot analysis revealed that p53 activation significantly exacerbated cardiomyocyte ferroptosis and oxidative stress damage, and diminished the anti-ferroptosis effects of AIG1 overexpression in DOX-treated cardiomyocytes (**Figure 6A**). Additionally, Nutlin-3a treatment recapitulated the dysregulation of iron and lipid peroxide in AIG overexpression cardiomyocytes following DOX stress (**Figure 6B**), as evidenced by aggravated intracellular and mitochondrial iron overload and lipid peroxide accumulation (**Figure 6B-F**). Likewise, Nutlin-3a abrogated protective roles of AIG1 overexpression, including decreased mitochondrial superoxide levels (**Figure 6B and G**), restored MMP (**Figure 6B and H**), reduced MDA levels (**Figure 6I**), and elevated GSH/GSSG ratio (**Figure 6J**) in DOX-stressed HL-1 cardiomyocytes.

Furthermore, we investigated whether Pirh2 inhibition also dampened the anti-ferroptosis effects of AIG1 overexpression in cardiomyocytes. Pirh2 was knocked down in HL-1 cardiomyocytes (**Figure S10A-C**) with or without AIG1 overexpression by transfecting adenoviruses carrying vectors encoding shRNA targeting Pirh2, and the cells were subjected to DOX treatment. Pirh2 knockdown resulted in increased p53 protein abundance and significantly activated the ferroptosis pathway, eliminating the protective effects of AIG1 overexpression against ferroptosis and oxidative stress, as demonstrated by Western blot analysis (**Figure 6K**). In addition, Pirh2 knockdown ablated AIG1 overexpression-induced dropped proportion of PI-positive cardiomyocytes (**Figure S9A**), decreases in iron accumulation (**Figure S9B**), lipid peroxide (**Figure S9C**), and mitochondrial superoxide production (**Figure S9D**), preserved MMP (**Figure S9E**), increased cell viability (**Figure S9F**) and ATP synthesis (**Figure S9G**), reduced LDH release (**Figure S9H**) and MDA levels (**Figure S9I**), and elevated GSH/GSSG ratio (**Figure S9J**) in cardiomyocytes. These findings suggest that AIG1 overexpression alleviates DOX-induced ferroptosis through the governance of Pirh2/p53 axis in cardiomyocytes.

To further assess causality *in vivo*, C57BL/6J male mice with or without cardiac AIG1 overexpression were subjected to Nutlin-3a administration to activate p53 under DOX challenge. Additionally, an AAV9-cTNT-delivery system was utilized to knock down Pirh2 expression in the hearts of C57BL/6J male mice (**Figure S10D-J**) with or without cardiac-specific AIG1 overexpression, followed by DOX administration.

In keeping with our HL-1 cardiomyocytes results, either p53 activation (**Figure 6L**) or Pirh2 knockdown (**Figure S11A-B**) significantly exacerbated cardiac ferroptosis and adverse remodeling in DIC, diminishing the anti-ferroptosis effects of AIG1 overexpression, as evidenced by Western blot analysis. Moreover, AIG1 overexpression mice exhibited reduced plasma levels of myocardial injury markers (**Figure 6M-N and S11C-D**), lowered cardiac MDA levels (**Figure 6O and S11E**), and a higher cardiac GSH/GSSG ratio (**Figure 6P and S11F**). However, these beneficial effects were nullified by p53 activation (**Figure 6M-P**) or cardiac-specific Pirh2 knockdown (**Figure S11C-F**). Functionally, Nutlin-3a treatment (**Figure 6Q-S**) or Pirh2 inhibition (**Figure S11G-I**) remarkably deteriorated DOX-evoked cardiac systolic dysfunction and diminished the improvements in LVEF and LVFS conferred by AIG1 overexpression upon DOX challenge. Histologically, p53 activation aggravated DOX-induced cardiac atrophy and fibrosis (**Figure 6S-V**), further reduced cardiac GPX4 abundance (**Figure 6S and W**), and promoted ROS and 4-HNE overproduction (**Figure 6S and X-Y**), thereby abolishing the favorable effects of AIG1 overexpression (**Figure 6S-Y**). Similar histopathology findings were observed in cardiac-specific Pirh2 knockdown mice (**Fig S11I-O**). Collectively, our findings suggest that the anti-ferroptosis effects and cardioprotection conferred by AIG1 overexpression are Pirh2/p53-dependent during DIC.

### Pharmacological inhibition of p53 or cardiac-specific overexpression of Pirh2 alleviates DOX-evoked cardiomyocyte ferroptosis and reverses AIG1 deletion-induced worsened cardiotoxicity

**Figure 7:**
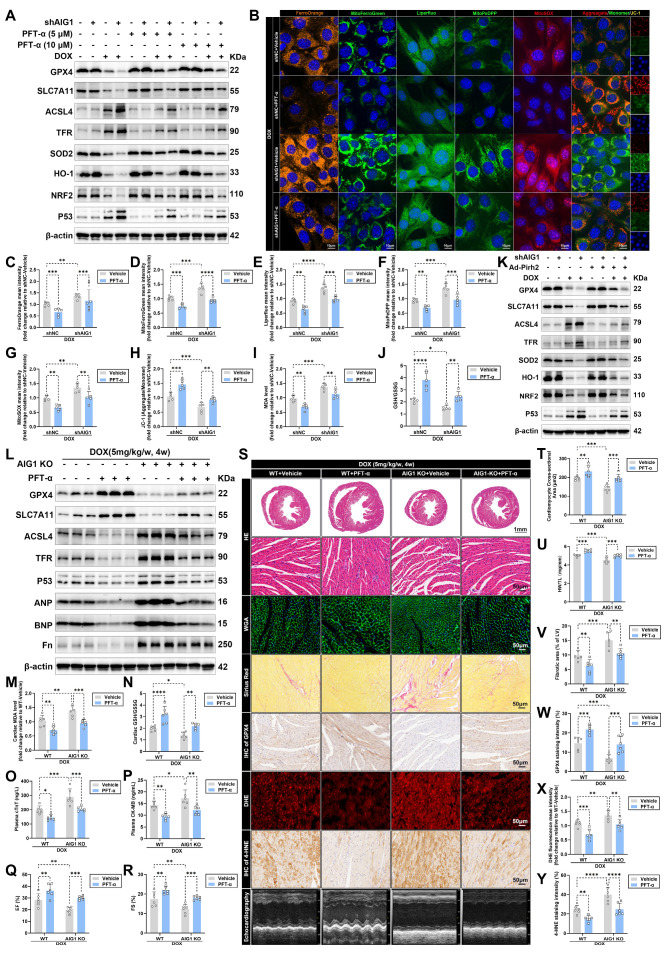
** Pharmacological inhibition of p53 alleviates DOX-evoked cardiomyocyte ferroptosis and reverses AIG1 deletion-induced worsened cardiotoxicity. (A)** through** (J)** HL-1 cardiomyocytes were exposed to DOX (1 μM, 24 h) in the absence or presence of AIG1 knockdown or PFT-α (5 μM or 10 μM, 24 h). **(A)** Representative immunoblotting images of protein expression of ferroptosis and oxidative stress markers in HL-1 cardiomyocytes under various treatment settings. (n = 3 per group). **(B)** Representative fluorescence images of FerroOrange staining, MitoFerroGreen staining, Liperfluo staining, MitoPeDPP staining, MitoSOX staining, and JC-1 staining in DOX-treated HL-1 cardiomyocytes in the absence or presence of AIG1 knockdown or PFT-α (10 μM, 24 h). Nuclei were counterstained with Hoechst 33342 (blue).** (C)** Quantification of intracellular iron level (FerroOrange staining) in HL-1 cardiomyocytes with indicated treatment. (n = 5 per group). **(D)** Quantification of mitochondrial iron level (MitoFerroGreen staining) in HL-1 cardiomyocytes with indicated treatment. (n = 5 per group).** (E)** Quantification of intracellular lipid peroxide (Liperfluo staining) in HL-1 cardiomyocytes with indicated treatment. (n = 5 per group). **(F)** Quantification of mitochondrial lipid peroxide (MitoPeDPP staining) in HL-1 cardiomyocytes with indicated treatment. (n = 5 per group). **(G)** Quantification of mitochondrial superoxide (MitoSOX staining) in HL-1 cardiomyocytes with indicated treatment. (n = 5 per group).** (H)** Quantification of mitochondrial membrane potential (JC-1 staining) in HL-1 cardiomyocytes with indicated treatment. (n = 5 per group).** (I)** and** (J)** MDA levels and GSH/GSSG ratio in HL-1 cardiomyocytes with indicated treatment. (n = 5 per group).** (K)** Representative immunoblotting images of protein expression of ferroptosis and oxidative stress markers in HL-1 cardiomyocytes treated with or without DOX (1 μM, 24 h) in the absence or presence of AIG1 knockdown or Pirh2 overexpression. (n = 3 per group).** (L)** through **(Y)** WT and AIG1 KO mice were intraperitoneally (i.p.) injected with PFT-α (10 mg/kg/day) throughout the DOX treatment period. (n = 6 per group). **(L)** Heart tissues samples were analyzed by Western blots analysis. **(M)** and** (N)** MDA levels and GSH/GSSG ratio in heart tissues.** (O)** and **(P)** Plasma levels of cTnT and CK-MB in mice. **(Q)** through** (S)** Quantification of LVEF** (Q)** and LVFS **(R)** shown with representative M-mode images **(S)** from transthoracic echocardiography.** (S)** and** (T)** Representative HE staining and WGA staining of hearts and quantitative analysis of cardiomyocyte areas.** (U)** Heart weight (HW)-to-tibial length (TL) ratio in mice. **(S)** and **(V)** Representative Picrosirius Red staining images of hearts and quantitative analysis of cardiac interstitial fibrosis.** (S)** and **(W)** Representative immunohistochemical staining of GPX4 in hearts and quantitative analysis of GPX4 staining intensity. **(S)** and** (X)** Representative fluorescence images and quantification of DHE staining in hearts. **(S)** and** (Y)** Representative immunohistochemical staining of 4-HNE in hearts and quantitative analysis of 4-HNE staining intensity. Data are presented as Mean ± SEM. *p < 0.05, **p < 0.01, ***p < 0.001, ****p < 0.0001. For statistical analysis, two-way ANOVA with Tukey's test for multiple comparisons was used for **C-J**, **M-R**, and **T-Y**.

Given that p53 has been identified as an essential target for regulating DOX-induced ferroptosis in both our findings and previous studies 17, 21, 23, 46, 50, 51, we evaluated whether p53 inhibition exerts a vital protective role in DIC. p53 inactivation in HL-1 cardiomyocytes was accomplished by PFT-α (a p53 inhibitor) treatment. Compared to the control group, p53 inhibition significantly mitigated ferroptosis and oxidative injury in cardiomyocytes subjected to DOX stress, and also negated the detrimental effects caused by AIG1 knockdown (**Figure 7A**). Immunofluorescence staining (**Figure 7B**) revealed that the worsened iron deposits, lipid peroxide, mitochondrial superoxide overproduction, and severe loss of MMP trigged by AIG1 knockdown were markedly cancelled off by PFT-α treatment in DOX-stressed cardiomyocytes (**Figure 7B-H**). Furthermore, AIG1 knockdown led to increased levels of MDA and a dropped GSH/GSSG ratio in cardiomyocytes upon DOX challenge, both of which were mitigated by PFT-α treatment (**Figure 7I-J**).

As expected, adenoviruses-mediated Pirh2 overexpression (**Figure S13A-C**) also alleviated ferroptosis and oxidative stress in DOX-treated cardiomyocytes* in vitro* (**Figure 7K**). Moreover, the destructive effects of AIG1 knockdown in DOX-stressed cardiomyocytes, including an elevated proportion of PI-positive cardiomyocytes (**Figure S12A**), iron overload (**Figure S12B**), heightened lipid peroxide (**Figure S12C**), mitochondrial superoxide overproduction (**Figure S12D**), reduced MMP (**Figure S12E**), decreases in cell viability (**Figure S12F**) and ATP synthesis (**Figure S12G**), increased LDH release (**Figure S12H**), elevated MDA levels (**Figure S12I**), and declined GSH/GSSG ratio (**Figure S12J**), were significantly ameliorated by Pirh2 overexpression (**Figure S12A-J**). Similarly, an AAV9-cTNT-delivery system was employed to overexpress Pirh2 in hearts (**Figure S13D-J**) of WT or AIG1 KO mice to further confirm these *in vitro* findings, followed by DOX treatment. Western blot analysis revealed that cardiac-specific overexpression of Pirh2 notably inhibited p53 activation, ameliorated cardiac ferroptosis and myocardial injury (**Figure S14A-F**), enhanced cardiac systolic function (**Figure S14G-I**), and mitigated pathological cardiotoxicity (**Figure S14I-O**), therefore counteracting the detrimental effects of AIG1 knockout following DOX challenge (**Figure S14A-O**). These findings further highlight Pirh2 as a critical mediator in AIG1-dependent protection against DIC through the inhibition of p53 signaling.

Having established the essence of AIG1-Pirh2-p53 signaling axis in DIC, we finally investigated the therapeutic potential of pharmacological p53 inhibition using PFT-α in chronic DIC mouse model. Both WT and AIG1 KO male mice were treated with PFT-α under DOX administration. Our results demonstrated that PFT-α significantly alleviated the activation of DOX-evoked cardiac ferroptosis pathway (**Figure 7L**), as well as unfavorable changes in MDA levels (**Figure 7M**), GSH/GSSG ratio (**Figure 7N**), and plasma levels of cTNT and CK-MB (**Figure 7O-P**) in both WT and AIG1 KO mice. Additionally, PFT-α improved cardiac systolic function, as measured by LVEF and LVFS (**Figure 7Q-S**), alleviated cardiac atrophy and fibrosis (**Figure 7S-V**), elevated cardiac GPX4 abundance (**Figure 7S and W**), and reduced ROS and 4-HNE levels (**Figure 7S and X-Y**) in DOX-challenged WT and AIG1 KO mice. Taken together, our findings clearly indicate the protective role of PFT-α against DOX-induced cardiomyocyte ferroptosis and cardiotoxicity.

## Discussion

**Figure 8:**
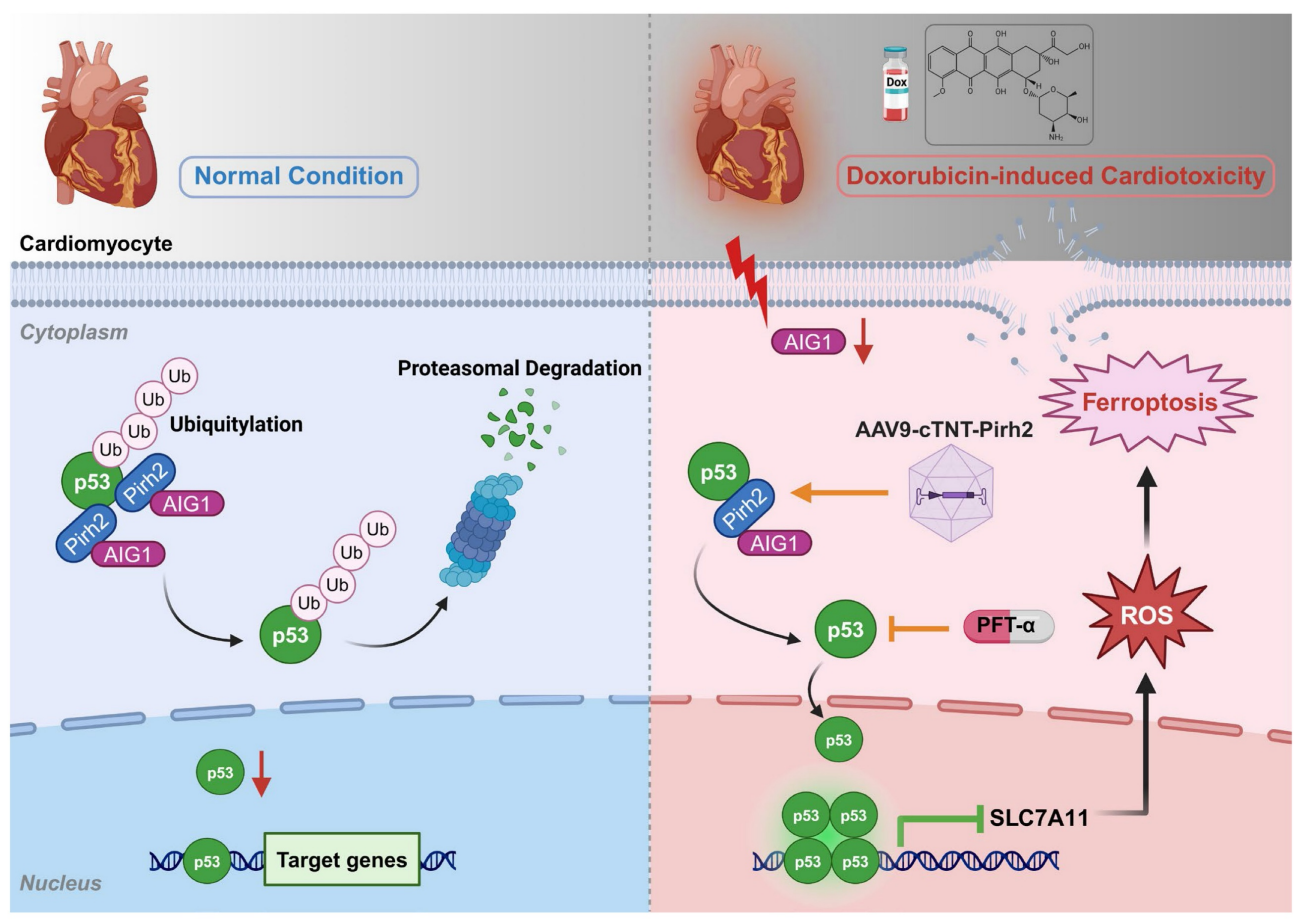
** A working model for AIG1-regulated ferroptosis in DOX-induced cardiotoxicity (DIC).** AIG1 was downregulated in cardiomyocyte following DOX stress, which bound less Pirh2 and thus inhibited Pirh2 interaction with p53. The ubiquitination of p53 was therefore decreased resulting in its enhanced deubiquitination and accumulation. The activation of p53 accelerated cardiomyocyte oxidative stress, ferroptosis and ultimately promoted DIC. AAV9, adeno-associated virus serotype 9; Ub, ubiquitin. The diagram was created using BioRender (https://www.biorender.com/).

The salient findings from our current study revealed a protective role of AIG1 against DIC. Our results for the first time discovered and demonstrated that DOX challenge significantly downregulated AIG1 expression in the heart and prompted myocardial ferroptosis, cardiac adverse remodeling, as well as contractile dysfunction in mice, the effects of which were accentuated and ameliorated, respectively, by cardiac-specific deletion and overexpression of AIG1. Furthermore, knockdown of AIG1 aggravated while overexpression of AIG1 attenuated DOX-induced ferroptosis, oxidative stress, and mitochondrial dysfunction in cultured cardiomyocytes. Mechanistically, AIG1 was found to directly interact with Pirh2, an E3 ubiquitin ligase of p53, thereby contributing to the ubiquitination and subsequent proteasomal degradation of p53 and ultimately mitigating cardiomyocyte ferroptosis. Importantly, treatment with a selective p53 inhibitor PFT-α provided protection against DOX-induced ferroptosis and cardiotoxicity. Collectively, our work represents the first study to delineate the vital role of AIG1 in the pathogenesis of DIC through mechanisms involving cardiomyocyte ferroptosis and oxidative stress in a Pirh2/p53-dependent fashion (**Figure 8**). These findings display the promises of the AIG1-Pirh2-p53 signaling axis as a novel therapeutic target for DIC.

DOX is a commonly used chemotherapeutic agent that induces progressive, chronic, and life-threatening cardiotoxicity (DIC) 1, 2, characterized by detrimental structural and functional alterations in the heart, including cardiac atrophy, compromised cardiac contractility, ventricular dilation in concert with cell death, oxidative stress, and mitochondrial dysfunction, all of which greatly limit the efficacy of DOX in treating various malignancies 2-8. The prevalence of DIC has increased in recent years due to rising cancer survival rates. Thus, identifying effective therapeutic targets to prevent DIC is imperative. Among the plethora of mechanisms postulated to be involved in DIC, cardiomyocyte ferroptosis has emerged as an essential component 13-18. In fact, a pivotal study published in 2019 was the first to demonstrate that ferroptosis, rather than other forms of cell death, plays a key role in the progression of DIC 13. Notably, these findings were further consolidated by subsequent another independent study indicating that GPX4 overexpression significantly improved cardiac performance following DOX exposure in mice by effectively suppressing ferroptosis 15. Interestingly, this conclusion was also corroborated by our prominent findings of significantly improved myocardial systolic function, diminished cardiac adverse remodeling and increased cardiomyocyte survival with ferroptosis inhibitor Fer-1 treatment after DOX challenge. Therefore, inhibition of ferroptosis might represent a critical therapeutic strategy against DIC.

AIG1, belonging to the androgen-inducible genes family characterized by the presence of the AIG/FAR-17a domain in its protein sequence 25, is predicted to a protein of approximately 28 kDa with five or six proposed transmembrane domains and was originally discovered as an androgen-induced gene product from human dermal papilla cells 52. In humans and rodents, the androgen-inducible genes family comprises only two members including AIG1 and ADTRP, of which the cell or tissue distributions are quite distinct 25. ADTRP is primarily expressed in endothelial cells and is recognized as a susceptibility gene for coronary artery disease in the Han Chinese population 53, which is closely associated with increased risk of coronary heart disease and atherosclerosis by regulating multiple downstream targets involved in coagulation, inflammation, endothelial function, and vascular integrity 54-58. However, AIG1, as a member of the same family, was predominately expressed in cardiomyocytes within heart tissues supported by our results and single-cell transcriptomics in human heart. Its functions remain largely underexplored in the context of cardiovascular diseases. Herein, our findings suggested that DOX stress dramatically suppressed AIG1 expression in cardiomyocytes, while AIG1 overexpression significantly alleviated DIC by blocking cardiomyocyte ferroptosis and oxidative stress. In contrast, knockout of AIG1 had detrimental effects on the progression of DIC via aggravating ferroptosis and oxidative damage. These findings highlighted the potential of AIG1 as a therapeutic target in cardiovascular diseases, particularly in DIC. Notably, we also found that the expression levels of AIG1 in the heart were not significantly different between male and female mice under normal condition or DIC, consistent with previous study 35. This suggests that, to a large extent, the role of AIG1 in DIC is independent of sex, although only male mice were utilized in our study to investigate the precise role of AIG1 in DIC.

The tumor suppressor protein p53 plays a critical role in cellular response to various stresses, including DNA damage, hypoxia, nutrition starvation and oncogene activation 59. During DIC, p53 is activated through oxidative DNA damage-ataxia telangiectasia mutated-p53 pathway induced by DOX stress 8, 60 and has been implicated in regulating cardiomyocyte ferroptosis through the transcriptional regulatory mechanisms during DIC 44, 59, 61-63. For instance, p53 can enhance ferroptosis by directly inhibiting the expression of SLC7A11 64, which is responsible for the uptake of extracellular cystine to supplement cellular cysteine, an essential biosynthesis precursor to GSH, that inhibits lipid peroxidation and ferroptosis. Additionally, p53 can promote ferroptosis via upregulating the expression of spermidine/spermine N1-acetyltransferase 1 65 or glutaminase 2 65. Interestingly, a recent study demonstrated that DOX-induced p53 interacted with parkinsonism associated deglycase (Park7) and accelerated its degradation likely through ubiquitination 17, resulting in iron homeostasis dysregulation, ROS overproduction, and ultimately cardiomyocyte ferroptosis by disrupting Park7-dependent Fe-S cluster/IRP-IRE regulatory pathway, underscoring that p53 activation plays a critical role in modulating DOX-induced ferroptosis, particularly through the transcription-independent mechanisms involving iron dyshomeostasis, an essential upstream trigger of ferroptosis. Moreover, accumulating evidence revealed that inhibition of p53 effectively reversed DOX-induced cardiotoxicity 17, 23, 66, 67, highlighting p53 signaling as a feasible therapeutical target for DIC. In line with these findings, our results hinted that AIG1 exerts anti-ferroptosis effects possibly by facilitating p53 protein degradation to inhibit its activation during DIC, and pharmacological inhibition of p53 effectively alleviated iron overload, lipid peroxidation, and ROS overproduction, synergistically suppressed cardiomyocyte ferroptosis, and ultimately protected against DIC in mice, markedly abrogating the detrimental effects of AIG1 inhibition.

The degradation and activity of p53 protein are regulated by various post-translational modifications, including ubiquitination, which significantly influences its efficacy in regulating ferroptosis by altering its stabilization, nuclear localization, and transcriptional activation 23, 46, 50, 68, 69. Pirh2, a critical E3 ubiquitin ligase whose role in cardiovascular diseases especially DIC remains poorly understood as far as our knowledge extends, is closely involved in such cellular processes as proliferation, cell cycle regulation, as well as cellular migration and plays a crucial role in different diseases and pathologies including but not limited to various cancers by directing ubiquitinating p53 and promoting its degradation 36. Importantly, AIG1 has been identified as an interaction partner and co-regulator of the Pirh2 E3 ligase through yeast two-hybrid screening 32, strongly prompting us to speculate that AIG1 regulates cardiomyocyte ferroptosis potentially by interacting with Pirh2 and modulating p53 signaling in the context of DIC. As expected, our study revealed that the anti-ferroptosis and oxidative stress effects of AIG1 were accomplished in a Pirh2/p53 axis-dependent manner through a mechanism of action involving directly binding with upstream Pirh2 E3 ubiquitin ligase of p53 and accelerating the ubiquitination and degradation of p53. Importantly, we observed that cardiac-specific Pirh2 overexpression significantly mitigated cardiomyocyte ferroptosis following DOX exposure and countered AIG1 knockdown-induced aggravated cardiomyocyte ferroptosis and oxidative stress by inhibiting p53 signaling.

It is important to point out that p53 signaling regulates multiple conventional forms of programmed cardiomyocyte death, in addition to ferroptosis, such as apoptosis and autophagy by functioning as a critical transcriptome regulator 70, which may play a significant role in DIC as previously reported 71, 72. However, our findings demonstrated that AIG1 specifically participates in regulating DOX-induced cardiomyocyte ferroptosis, instead of other p53-mediated cell death pathways such as apoptosis, necroptosis, autophagy, or pyroptosis, likely through its interaction with Pirh2 to modulate p53 signaling during DIC. We speculated that there are several possible mechanistic and contextual factors accounting for this discrepancy. As previously discussed, p53 regulatory activity is tightly modulated by ubiquitination, which dictates its stability, localization, and transcriptional selectivity in the context of DIC 23, 49, 69. For example, E3 ubiquitin ligase Mitsugumin-53 (MG53) has been reported to inhibit DOX-induced cardiomyocyte ferroptosis by targeting p53-mediated SLC7A11/GPX4 pathway 23, whereas mouse double minute 2 (MDM2)-mediated p53 ubiquitination can regulate the mitochondrial autophagy 48 and apoptosis 48, 49, 73 in cardiomyocytes under DOX stress, suggesting that the specific interacting dynamics between different E3 ubiquitin ligases and p53, may result in distinct ubiquitination patterns, and had significant effects on determining p53-dependent downstream signaling transduction under the context of DIC. Our present study revealed that the Pirh2-dependent p53 ubiquitination mediated by AIG1 specifically mitigates DOX-induced ferroptosis rather than other cardiomyocyte death modalities, underscoring the uniqueness of AIG1/Pirh2 axis in regulating cardiomyocyte ferroptosis in the context of DIC. However, the detailed molecular mechanisms regarding the specific ubiquitination sites on p53 mediated through the AIG1/Pirh2 interaction in DOX-stressed cardiomyocyte remain to be further elucidated. Additionally, an increasing number of studies have demonstrated that ferroptosis represents a more prominent form of cardiomyocyte death compared to p53-dependent apoptosis, autophagy or necroptosis, especially under continues DOX exposure of chronic DIC model, which is closely related to sustained oxidative stress-induced typical lipid peroxidation and iron overload 13, 15, 17. Our findings consistently revealed that cardiomyocyte ferroptosis gradually worsens with cumulative DOX burden (**Figure 1A**), and DIC progression is effectively reversed through the inhibition of ferroptosis (**Figure 1K-X**) in the chronic DIC model. However, caspase-dependent apoptosis triggered by p53 activation may occur at an earlier stage of DIC treatment and requires a substantially high dose of DOX exposure 24, 74. More importantly, it has been demonstrated that p53 may also play diverse or even opposite roles in regulating cell death depending on different cell types and pathological stimuli 24, 44. Recent evidence suggests that p53 involvement in DOX-induced apoptosis is more complex than originally thought, as it depends on the duration of DOX treatment as well as the administered dose 24. In the acute setting, p53 blockade attenuates apoptotic death; however, chronic p53 deletion augments cardiomyocyte apoptosis 75, 76. It is therefore understandable that p53 may play a more critical role in regulating cardiomyocyte ferroptosis likely through transcriptional and post-translational mechanisms that directly modulate lipid peroxidation and iron homeostasis under sustained DOX exposure in the chronic DIC model 17, 23. Collectively, our study further emphasizes that the specific AIG1-Pirh2-p53 interacting molecular network plays a crucial role in selectively regulating p53-mediated downstream ferroptosis signaling in the DIC-specific context.

Notably, although p53 is a key tumor suppressor that can enhance ferroptosis in cancer 44, 59, its function is generally nullified or rendered inactive due to p53 mutations in the majority of cancers treated with DOX 77. Recent evidence showed that Pirh2 upregulation promoted proliferation of cancer cells and conferred survival advantages by facilitating p53 degradation 36. Based on our findings regarding the Pirh2/p53 axis, it is reasonable to predict that healthy organs may experience more ferroptotic damage than cancer cells during DOX treatment. Interestingly, a prior study demonstrated a positive correlation between AIG1 expression levels and the survival of hepatocellular carcinoma patients 32, seeming to indicate a potential contradiction between AIG1 upregulation and the Pirh2/p53 axis in cancer contexts. Thus, further studies are urgently required to clarify the precise roles of AIG1 in cancers and to determine whether circumventing the AIG1-Pirh2-p53 signaling pathway in future cancer chemotherapeutics could improve safety profiles.

There are several experimental limitations in our current study. First, although intravenous injection of AAV9 maneuver was employed to achieve AIG1 overexpression or knockdown in CMs of mouse hearts using cardiac-specific promoter cTNT 31, 37, 78, we utilized a global knockout of AIG1 to investigate its loss-of-function effects on DOX-induced cardiomyocyte ferroptosis and cardiotoxicity in mice, which may introduce confounding factors due to compensatory events from AIG1 knockout in other organs, potentially masking the real effects of AIG1 knockout in DIC 26. Therefore, it is important that cardiomyocyte-specific AIG1-deficient or overexpression transgenic mice would be necessary to examine the specific role of AIG1 in the pathology of cardiomyocytes during DIC. Similarly, although our results shown that Pirh2 is a critical downstream factor responsible for AIG1-mediated regulation of cardiomyocyte ferroptosis through inhibition of p53 activation *in vitro* and* in vivo*, a cardiac-specific Pirh2 gene-editing transgenic mouse model would be more appropriate for elucidating its specific contributions in DIC. Last but not least, the effects of AIG1 were not assessed in a tumor-bearing mouse model of DIC and AIG1 expression levels were unable to be validated in human heart samples of DIC due to apparent difficulties to access human samples. As we discussed before, more studies are warranted to further confirm the benefits of AIG1 in DIC among cancer patients and to uncover its broader roles in cancer contexts.

## Conclusions

In summary, our current findings demonstrate for the first time that AIG1 plays a critical cardioprotective role in DIC by inhibiting Pirh2/p53-dependent cardiomyocyte ferroptosis. This discovery proposes a novel perspective on the pathogenesis and potential therapeutic strategies for DIC, and also provides valuable insights for future research into the role of AIG1 in other cardiovascular diseases.

## Supplementary Material

Supplementary methods, figures and tables.

## Abbreviations

AAV9: adeno-associated virus serotype 9
ACSL4: acyl-CoA synthetase long-chain family member 4
AIG1: androgen-induced gene 1
AMCMs: adult mouse cardiomyocytes
ANP: atrial natriuretic peptide
Baf A1: bafilomycin A1
BNP: b-type natriuretic peptide
CFs: cardiac fibroblasts
CHX: cycloheximide
CK-MB: creatine kinase-myocardial band
CMs: cardiomyocytes
COL3A1: collagen type III alpha 1 chain
Co-IP: Co-immunoprecipitation
cTnT: cardiac troponin T
DFO: deferoxamine
DIC: DOX-induced cardiotoxicity
DOX: doxorubicin
DXZ: dexrazoxane
ECs: endothelial cells
ER: endoplasmic reticulum
FAHFA: fatty acid esters of hydroxy fatty acid
Fer-1: ferrostatin-1
Fn: fibronectin
GPX4: glutathione peroxidase 4
HO-1: heme oxygenase 1
HW/TL: heart weight-to-tibial length
KO: knockout
LDH: lactate dehydrogenase
LVEF: left ventricular ejection fraction
LVFS: left ventricular fractional shortening
LVSF: left ventricular systolic function
MDA: malondialdehyde
MMP: mitochondrial membrane potential
MG53: mitsugumin-53
MDM2: mouse double minute 2
NRF2: nuclear factor erythroid 2-related factor 2
OCR: oxygen consumption rate
PI: propidium iodide
PLA: proximity ligation assay
RCHY1: CHY zinc finger domain containing 1
ROS: reactive oxygen species
SLC7A11: solute carrier family 7 member 11
SOD2: superoxide dismutase 2
TFR: transferrin receptor
TGFβ1: transforming growth factor beta 1
WT: wild type
3-MA: 3-methyladenine
4-HNE: 4-hydroxynonenal
