# Supplementary material for: AIG1 protects against doxorubicin-induced cardiomyocyte ferroptosis and cardiotoxicity by promoting ubiquitination-mediated p53 degradation
Source: Theranostics. 2025 Mar 31;15(11):4931–54. doi: 10.7150/thno.108410 (PMC12036874; doi:10.7150/thno.108410)
Supplement: Supplementary file 1 — Supplementary methods, figures and tables. [file thnov15p4931s1.pdf]

SUPPLEMENTAL MATERIAL

AIG1 protects against doxorubicin-induced cardiomyocyte ferroptosis and cardiotoxicity by promoting ubiquitination-mediated p53 degradation

Runing Title: AIG1 prevents DOX-induced cardiomyocyte ferroptosis and cardiotoxicity

Yuekai Shi, etc.

#**Correspondence to:** Junbo Ge, MD, PhD, Jianying Ma, MD, PhD, and Rende Xu, MD, PhD. Department of Cardiology, Shanghai Institute of Cardiovascular Diseases, Zhongshan Hospital, Fudan University, 180 Fenglin Road, Xuhui District, Shanghai, 200032, China. E-mail: jbge@zs-hospital.sh.cn; ma.jianying@zs-hospital.sh.cn; xu.rende@zs-hospital.sh.cn

This file includes:

Supplementary Materials and Methods

Supplementary Figures: Figure S1-S14

Supplementary Tables: Table S1-S4

Supplementary Materials and Methods

Experimental animal models

Global AIG1 knockout mice were generated by GemPharmatech Co., Ltd (Jiangsu, China) using CRISPR-Cas9 strategy. Briefly, two gRNAs (guide RNAs, gRNA1 and gRNA2) and Cas9 expression plasmids were designed to delete exon 2-4 of AIG1, resulting in disruption of AIG1 protein function. Cas9 mRNA and gRNAs were generated by *in vitro* transcription. Two gRNAs were used to identify the target sequence that contains AIG1 in the present study, and were listed in **Supplementary Table S1**. Cas9 mRNA and gRNAs were microinjected into fertilized eggs of C57BL/6JGpt mice to obtain positive F0 mice which were confirmed by PCR and on-target amplicon sequencing. A stable F1-generation mouse strain was obtained by mating positive F0-generation mice with C57BL/6JGpt mice and confirmation of the desired mutant allele was carried out by PCR and on-target amplicon sequencing.

To overexpress AIG1 or Pirh2 in mouse hearts, an aliquot of 100 μL (diluted in sterile PBS) adeno-associated virus serotype 9 (AAV9) particles carrying cardiac troponin T (cTnT) promoter driven mouse AIG1 cDNA (AAV9-AIG1) or Pirh2 cDNA (AAV9-Pirh2) or empty AAV9-cTnT served as vector control (AAV9-Vector) (Hanbio Biotechnology Co., Ltd. Shanghai, China) at a density of 1 $\times 10^{12}$ v.g./mL were single injected into 5-week-old male mice (GemPharmatech Co., Ltd, Jiangsu, China) through tail veins. Three weeks after transfection, AIG1 overexpression efficiency was examined in mouse heart tissues and other organs.

To knockdown AIG1 or Pirh2 expression in mouse hearts, the AIG1 or Pirh2 inhibitory AAV9 construct (AAV9-cTnT-shAIG1 or AAV9-cTNT-shPirh2) and the control virus (AAV9-cTnT-shNC), all driven by cTnT promoter, were utilized (Hanbio Biotechnology Co., Ltd. Shanghai, China). An aliquot of 100 μL of AAV9 was injected (1 $\times 10^{12}$ v.g./mL) into C57BL/6J male mice or AIG1 overexpression mice via the tail vein as previously described. Cardiac tissues and other organs were collected. The knockdown efficiency of virus transfection was determined by measuring the expression levels of AIG1 or Pirh2 using quantitative PCR and Western blot analysis.

To establish the chronic DIC model, 8-week-old mice received a weekly intraperitoneal (i.p.) injection of DOX (5 mg/kg, D1515, Sigma-Aldrich, Germany) or saline for 4 weeks [1]. To inhibit ferroptosis, mice were given a daily i.p. injection of ferrostatin-1 (Fer-1, 1 mg/kg, HY-100579, MedChem Express, USA) 1 day before DOX injection [2]. To evaluate the effects of p53 inhibition or activation on the progression of DIC, Wild type (WT) and AIG1-KO mice received PFT-α (a selective inhibitor of p53; 10 mg/kg, HY-15484, MedChem Express, USA) i.p. injection once daily during the DOX treatment [3], while AAV9-Vector and AAV9-AIG1 mice received a daily i.p. injection of Nutlin-3a (a specific activator of p53; 20 mg/kg, HY-10029, MedChem Express, USA) throughout the DOX administration period [4, 5].

All mice were housed in a controlled environment with a 12/12 h light/dark cycle at a constant temperature of 22 ± 2 °C and 30 - 70% humidity with *ad libitum* availability to water and standard rodent chow. At the conclusion of each experiment, animals were humanely euthanized under general anesthesia using cervical dislocation under inhalation of 2 % isoflurane anesthesia. All animal studies adhered to the National

Institutes of Health Guidelines for the Care and Use of Laboratory Animals, and received approval from the Animal Care and Use Committee of Zhongshan Hospital, Fudan University.

Echocardiography

To assess the cardiac systolic function, transthoracic echocardiography was performed using a Vevo 2100 High Resolution Imaging System (Visual Sonics Inc, ON, Canada) while mice were anaesthetized with 2% isoflurane. The left ventricle was detected under the long-axis M-mode when heart rate was approximately 400-550 bpm. Left ventricular fractional shortening (LVFS) and left ventricular ejection fraction (LVEF) were recorded as previously described [6].

Histological assessment and immunohistochemistry staining

Mouse heart tissues were fixed in 4% paraformaldehyde and then embedded in paraffin and cut cross-sectionally into 5 μm-slides. After continuous deparaffinization and rehydration, paraffin slices were subjected to hematoxylin and eosin (HE) staining to assess general morphology, Alexa Fluor 488 labeled wheat germ agglutinin (WGA) staining to evaluate myocardial atrophy, Picrosirius Red (PSR) staining and Masson's trichrome staining to evaluate myocardial interstitial collagen content based on previous studies [1, 7-9]. Cardiac oxidative stress levels were determined by dihydroethidium (DHE) staining according to a previously described method [10]. For immunohistochemistry staining, following the antigen retrieval process, paraffin-embedded slices were incubated with primary antibodies against GPX4 (67763-1-Ig, Proteintech, Wuhan, China), 4-hydroxynonenal (4-HNE; ab46545, Abcam, USA) and P53 (10442-1-AP, Proteintech, Wuhan, China) respectively overnight at 4 °C and were then incubated with biotin-conjugated secondary antibodies. Positive antibody binding was visualized using a 3,3′-diaminobenzidine (DAB) peroxidase substrate kit, and nuclei were stained with hematoxylin. All Images were taken using a Leica microscope (Leica Microsystems, Germany) and further processed and analyzed using the Image J software (National Institutes of Health, Bethesda, MD, USA).

Biochemical detection

Mouse blood samples were collected and centrifuged for 15 min at 3000 rpm to obtain serum, which was stored at -80 °C. Cardiac troponin T (cTnT) and creatine kinase isoenzyme-myocardial band (CK-MB) levels were determined by a commercially available enzyme-linked immunosorbent assay kit (H149-4, H197-1, Nanjing Jiancheng Bioengineering Institute, Nanjing, China) according to the guideline from the manufacturer. GSH and GSSG levels were determined using a glutathione measurement package (S0053, Beyotime Biotechnology, Shanghai, China). Malondialdehyde (MDA) was determined by a lipid peroxidation MDA analysis kit (S0131, Beyotime Biotechnology, Shanghai, China). Lactate dehydrogenase (LDH) was assessed using a LDH kit (ab102526, Abcam, USA).

Isolation and culture of primary cells from adult mouse hearts

Adult mouse cardiomyocytes (AMCMs), cardiac fibroblasts (CFs) and endothelial cells (ECs) were isolated from the ventricles of WT, AIG1 KO, AAV9-Vector, and AAV9-AIG1 male mice using our previously described methods with some modifications [11, 12]. In brief, 2-month-old male mice were anaesthetized, and the chest was opened to expose

the heart. The descending aorta and inferior vena cava were cut, and the heart was immediately flushed by injecting 7 mL of EDTA buffer into the right ventricle. The heart was excised and transferred to a 60 mm dish containing fresh EDTA buffer. Digestion was achieved by the sequential injection of 10 mL of EDTA buffer, 3 mL of perfusion buffer, and 50 mL of collagenase buffer into the LV. The constituent chambers (atria, LV) were then separated and gently pulled into 1 mm^3 pieces using forceps. Cellular dissociation was completed by gentle trituration, and enzymatic activity was inhibited by the addition of 5 mL of stop buffer. The cell suspension was passed through a 100 μm filter, and pure cardiomyocytes were collected and subjected to 4 sequential rounds of gravity settling using 3 intermediate calcium reintroduction buffers to gradually restore the calcium concentration to physiological levels. Where needed, the cardiac myocytes were resuspended in prewarmed media, plated at an application-dependent density onto laminin (5 μg/mL)-precoated tissue culture plastic or glass coverslips, and cultured in a humidified tissue culture incubator (37 °C, 5% CO2). After 1 h, media were replaced with fresh, prewarmed culture media. In the supernatant containing non-myocyte cells, ECs were gathered using CD31-coupled microbeads and further cultured in endothelial culture medium. Cardiac fibroblasts were collected using MEFSK4-coupled microbeads and further cultured in DMEM complete medium. AMCMs were cultured in M199 medium (supplemented with 1% penicillin-streptomycin, 1% ITS, 1% CD lipid, 0.1% BSA, and 0.1% BDM) for further treatment. An overall yield of 70-80 % rod-shaped cardiomyocytes exhibiting clear sarcomere contour was considered a valid isolation. Those rod-shaped cardiomyocytes with distinct edges were chosen for following mechanical properties recording [9].

Cell shortening and relengthening

The contractile capacity of adult mouse cardiomyocyte was monitored by employing the assistance of a SoftEdge MyoCam system (IonOptix, Milton, MA, USA) with an Olympus IX-70 microscope [13]. Briefly, cells were resuspended with a contractile buffer and were stimulated to contract at a frequency of 0.5 Hz before the recording of mechanical properties including resting cell length, peak shortening (PS), maximal velocity of shortening (+dL/dt), and maximal velocity of relengthening (-dL/dt).

Cell culture, transfection and *in vitro* treatment

HL-1 cardiomyocytes and HEK293T cells were maintained in a Dulbecco's Modified Eagle Medium (DMEM, Gibco, USA), supplemented with fetal bovine serum albumin (FBS, 10%, Gibco, USA) in a humidified cell culture incubator (Thermo Fisher Scientific, USA) at 37 °C with 95% air and 5% CO2. The overexpression or knockdown of AIG1 or Pirh2 in HL-1 cardiomyocytes was generated using an adenovirus-mediated delivery system. All recombinant adenoviruses were purchased from Hanbio Biotechnology Co., Ltd. (Hanbio, Shanghai, China). Plasmids carrying target gene full length cDNA, or truncated mutated cDNA or the target gene shRNA were designed and synthesized by Hanbio Biotechnology Co., Ltd. (Hanbio, Shanghai, China), and sequences of shRNA are listed in **Supplementary Table S2**. The detailed adenoviral transfection procedure followed the manufacturer's instructions. In brief, cardiomyocytes were cultured in a proper density and then transfected with adenovirus at MOIs of 10 for 4 h and refreshed with DMEM for another 24 h and then for the subsequent experiments. The efficiency of

transfection with adenovirus was verified using Western blot. The transfection of plasmids encoding full-length and mutated AIG1 or Pirh2 in HEK293T cells was accomplished with the Lipofectamine 3000 reagent (L3000015, Invitrogen, USA) prior to cell lysis or staining and imaging 48 h later.

DOX (1 μM or 0.1μM, D1515, Sigma-Aldrich, Germany) treatment was used to establish a DIC model *in vitro* in HL-1 cardiomyocytes or AMCMs for 24 h. To evaluate the involvement of ferroptosis, the ferroptosis inducers erastin (5 μM, HY-15763, MedChem Express, USA) or RSL3 (2.5 μM, HY-100218A, MedChem Express, USA), ferroptosis inhibitor Fer-1 (2.5 μM, HY-100579, MedChem Express, USA) or deferoxamine (DFO, 5 μM, HY-B1625, MedChem Express, USA), the apoptosis inhibitor Z-VAD-FMK (Z-VAD, 10 μM, HY-16658B, MedChemExpress, USA), the autophagy inhibitor 3-Methyladenine (3-MA, 5 mM, HY-19312, MedChem Express, USA), the necroptosis inhibitor Necrostatin-1 (Nec-1, 2 μM, HY-15760, MedChem Express, USA) or the pyroptosis inhibitor MCC950 (10 μM, HY-12815, MedChem Express, USA) were supplemented to HL-1 cardiomyocytes or AMCMs for further experimentation. Cycloheximide (CHX, 10 μM, HY-12320, MedChem Express, USA) was used in a time-course treatment to determine protein half-life. 3-MA (5 mM, HY-19312, MedChem Express, USA), Bafilomycin A1(Baf A1, 50 nM, HY-100558, MedChem Express, USA), and MG132 (20 μM, HY-13259, MedChem Express, USA) were employed to determine protein degradation pathways. To evaluate p53 involvement in ferroptosis activation, Nutlin-3a (2.5 μM or 5 μM, HY-10029, MedChem Express, USA) or PFT-α (5 μM or 10 μM, HY-15484, MedChem Express, USA) was to induce p53 activation or inhibition in HL-1 cardiomyocytes.

Cell viability assays

Cell viability was evaluated using the Enhanced Cell Counting Kit-8 (CCK8) Assay Kit (C0042, Beyotime Institute of Biotechnology, Shanghai, China) according to the manufacturer’s instructions. Briefly, at the end of DOX treatment, the cell culture medium was replaced with fresh medium containing 10% CCK-8 solution. After incubation at 37 °C for 1.5 h, the absorbance was measured at 450 nm using Multi-Plate Reader (Biotek Synergy), and then cell viability was calculated.

ATP assay

Total cellular ATP content was assessed with a firefly luciferase-based enhanced ATP assay kit (S0027, Beyotime Institute of Biotechnology, Shanghai, China) according to the manufacturer's instructions. Briefly, cells were lysed in ATP lysis buffer and then centrifuged for 5 min at 4 °C and 12000 g to acquire the supernatant. Then, 20 μL of the supernatant from each sample and 100 μL of ATP detection working dilution were mixed quickly in a white, opaque 96-well plate, which was incubated at RT for 5 min. The total ATP content was measured by a microplate luminometer (BioTek, Synergy2) and normalized to the protein concentration.

Immunofluorescence staining and Imaging

Heart tissues were collected, embedded in OCT, frozen in liquid nitrogen and cut into 5-μm sections. Cultured cells were fixed in 4% polyformaldehyde in PBS for 15 min at RT. Then, tissue sections and cultured cells were permeabilized with 0.5% Triton X-100 in PBS for 10 min and incubated in 5% BSA for 1 h at RT. Then, the samples were incubated at 4 °C overnight with primary antibodies diluted in 1% BSA. After being

washed 3 times with PBS, the samples were stained with fluorescent secondary antibodies for 1 h at RT, followed by 20 min of staining with DAPI (62248, Invitrogen, USA) or/and F-actin (A12379, Invitrogen, USA) before being visualized by confocal microscopy (Leica Microsystems, Germany) or Operetta CLS High Content Imaging System (Perkin Elmer, USA). The following primary and fluorescent secondary antibodies were used: anti-AIG1 (ab140186, Abcam, USA), anti-alpha Actinin (ab18061, Abcam, USA), anti-Pirh2 (67856-1-Ig, Proteintech, Wuhan, China), anti-P53 (10442-1-AP, Proteintech, Wuhan, China), anti-rabbit IgG H&L Alexa Fluor 488 (ab150077, Abcam, USA), anti-rabbit IgG H&L Alexa Fluor 647 (ab150075, Abcam, USA), and goat anti-mouse IgG H&L Alexa Fluor 647 (ab150115, Abcam, USA).

Assessment of iron level and lipid peroxidation

Cellular or mitochondrial iron levels were determined by FerroOrange staining (1 μM, F374, DOJINDO, Japan) or Mito-FerroGreen staining (5 μM, M489, DOJINDO, Japan); cellular or mitochondrial lipid peroxides were determined by Liperfluo staining (5 μM, L248, DOJINDO, Japan) or MitoPeDPP staining (0.5 μM, M466, DOJINDO, Japan). Hoechst 33342 (H3570, Invitrogen) was used for nuclei staining. All living cell dyes were used per manufacturer's instructions. Fluorescence intensities were captured and quantified using an Operetta CLS High Content Imaging System (Perkin Elmer, USA) or BD FACS Celesta flow cytometry (BD Biosciences, USA). All results were presented as relative intensity using control group for normalization.

Measurement of the Mitochondrial Membrane Potential and Mitochondrial ROS

JC-1 (C2003S, Beyotime Biotechnology, Shanghai, China) was used to measure mitochondrial transmembrane potential (MMP). Briefly, cells were incubated with JC-1 solution for 20 minutes at 37˚C. After that cells were washed with dilution buffer for three times. MMP was measured by the aggregate fluorescence-to-monomer fluorescence intensity (red/green) ratio. MitoSOX (M36008, Thermo Fisher Scientific, USA) was used to detect the mitochondrial superoxide production. Briefly, cells were incubated with 5 μM MitoSOX for 10 minutes at 37˚C and then washed gently for three times. Hoechst 33342 was used for nuclei staining. The fluorescent product was measured by using Operetta CLS High Content Imaging System (Perkin Elmer, USA).

Determination of cell oxygen consumption

Oxygen consumption rate (OCR) of cardiomyocytes was measured using on a Seahorse XFe96 Extracellular Flux Analyzer (Agilent, USA) in conjunction with Seahorse Cell Mito Stress Test Kit (103015–100, Agilent, USA) per the manufacturer's instructions. Briefly, Cells were seeded into XFe96 plates at a proper density and treated as indicated. After specific treatment, the culture media were replaced with Seahorse XF medium, and the pH value of the medium was adjusted to 7.4. Then, the plate was incubated at 37 ℃ for 1 h in a CO_2-free incubator. OCR was measured sequentially in basal, oligomycin, trifluoromethoxy carbonylcyanide phenylhydrazone (FCCP), and Rotenone/Antimycin A environments. Values of OCR were normalized to total protein content determined by BCA method (P0009, Beyotime Biotechnology, Shanghai, China) which was quantified at the end of the Seahorse experiment.

Real-time quantitative PCR (real-time qPCR)

Total RNA was extracted from the mouse heart tissues and cells using TRIzol (Vazyme

Biotech, China) in accordance with the manufacturer's instructions. RNA concentration and purity were determined with a Nanodrop (Thermo Fisher Scientific, USA), and 1000 ng of purified RNA with an A260/A280 ratio of 1.8–2.0 was reverse transcribed into cDNA by Hifair® III 1st Strand cDNA Synthesis SuperMix for qPCR (11141ES60, Yeasen Biotechnology, Shanghai, China). Then, RT–PCR was performed using Hieff® qPCR SYBR Green Master Mix (11202ES08, Yeasen Biotechnology, Shanghai, China) on a QuantStudio™ 7 Pro Real-Time PCR detection system (Applied Biosystems, USA). The fold change in relative mRNA expression was calculated using the $2^{-\Delta\Delta Ct}$ method with β-actin as the internal control. The validated primer sequences produced by Sangon Biotech (Shanghai, China) used in this study were listed in **Supplementary Table S3**.

Protein extraction and Western blot analysis

Protein extracts were isolated from cardiac tissues and cells at 4 °C using RIPA lysis buffer (P0013; Beyotime Biotechnology, Shanghai, China) with protease and phosphatase inhibitor cocktail. Lysed tissues or cells were centrifugated at 12000 rpm for 15 min at 4 °C and protein lysates were transferred to a new Eppendorf tube. The protein concentration was determined using the BCA method (P0009, Beyotime Biotechnology, Shanghai, China) and then protein extractions were separated by SDS-PAGE and transferred onto PVDF membranes. After incubation with primary antibodies at 4 °C overnight, membranes were then incubated with respective HRP-conjugated secondary antibodies. Signals were imaged using a digital chemiluminescence system (Tanon, Shanghai, China) and quantification was performed with the Image J software (National Institutes of Health, Bethesda, MD, USA). The intensity of the β-actin band was used as a loading control for sample comparisons. The primary and secondary antibodies used in our study were listed in **Supplementary Table S4**.

Structure-based protein interaction interface analysis

The molecular structure of Pirh2（PDB ID：7YNX）was obtained from the RCSB PDB database (http://www.rcsb.org/). Protein structure of AIG1 (AF-Q9NVV5-F1), which was predicted using AlphaFold Protein Structure Database, a highly accurate artificial intelligence-based computational structure modeling method [14, 15], was retrieved from the UniProt database (http://www.uniprot.org/). Then the structures of these proteins were submitted to the HDOCK Server (http://hdock.phys.hust.edu.cn/) to predict their potential interaction interface [16]. Finally, the prediction results were visualized using the PyMol tool (http://pymol.org).

Co-immunoprecipitation (Co-IP)

Co-immunoprecipitation (Co-IP) was performed using a magnetic IP kit (88804, Thermo Fisher Scientific, USA). Briefly, after the samples were harvested, they were lysed at 4 °C in ice-cold IP lysis buffer for 30 minutes and then centrifuged (12000 × g, 10 minutes), following which equal volumes of lysates were incubated with various irrelevant IgG or specific antibodies and crosslinked onto the magnetic beads with rotation for 1 hour at room temperature. After removing the unbound sample, protein A/G magnetic beads were washed extensively with IP wash buffer, and the proteins were eluted by boiling in 1× SDS sample buffer before SDS-PAGE.

In situ proximity ligation assay (PLA) assay

Proximity ligation assays (PLA) were used to evaluate the protein interactions of AIG1-

Pirh2 (Red Starter kit) and Pirh2-p53 (Green Starter kit). The cells were washed, fixed, permeabilized, and blocked as described for the previous immunofluorescence staining process. The two primary antibodies raised in different species were then incubated with the samples overnight. The proximity ligation reaction and signal visualization were performed following the manufacturer's instructions (Duolink in situ PLA Detection Kit, Sigma-Aldrich). PLA signals were visualized and images were taken by Operetta CLS High Content Imaging System (Perkin Elmer, USA). The number of PLA signals per cell was quantified using the Duolink Image Tool software (Sigma Aldrich, Germany).

Statistical analysis

All data were shown as mean ± SEM. Statistical analysis was performed using the GraphPad Prism 9.4.1 software (GraphPad Software, La Jolla, CA, USA). Comparison between the two groups was conducted using unpaired student's t-test (normal distribution and equal variances). Multiple group comparisons were performed using one-way or two-way ANOVA (normal distribution) followed by Tukey's multiple comparison test. Differences between groups were classified as not statistically significant (ns), statistically significant (*$P < 0.05$), high significant (**$P < 0.01$), very significant (***$P < 0.001$) and the most significant (****$P < 0.0001$). In certain cases, raw values were normalized to the control group values. All experimental n numbers are provided in the figure legends.

Supplementary Figures

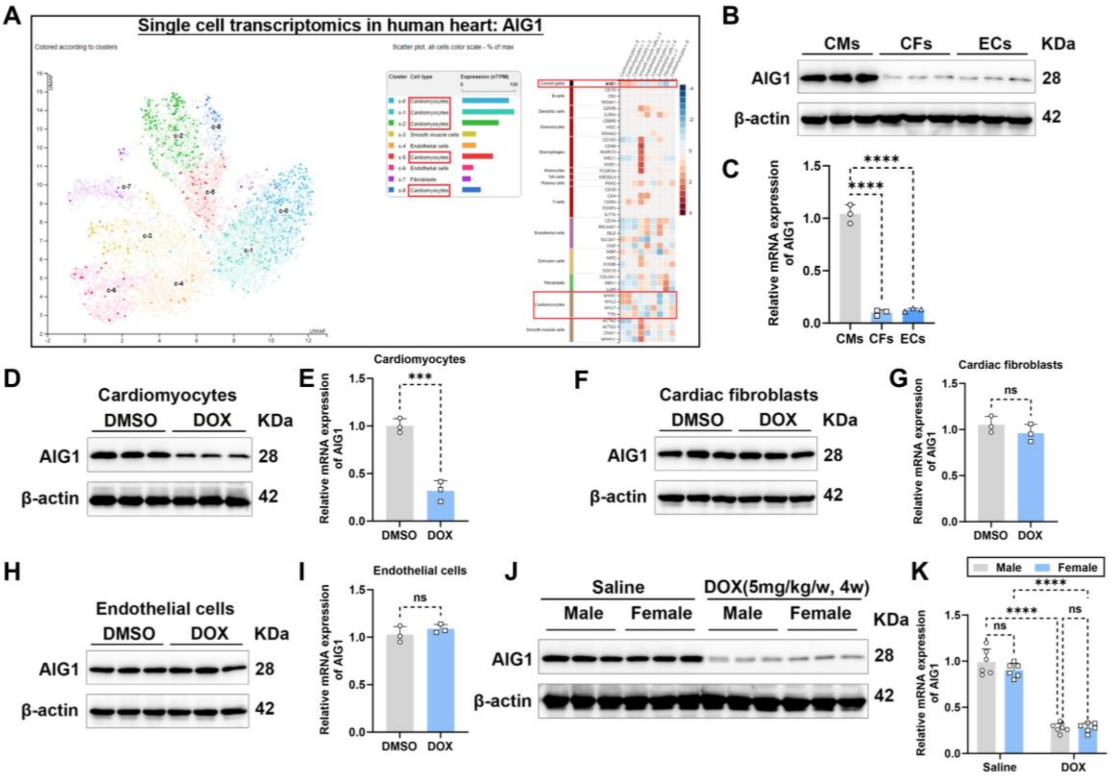

Figure S1: DOX decreases AIG1 expression in cardiomyocytes. (A) Cellular distribution of AIG1 mRNA in hearts, based on single cell-sequencing analysis from the Human Protein Atlas database. **(B)** Representative immunoblotting images of AIG1 in primary cardiomyocytes (CMs), cardiac fibroblasts (CFs), and endothelial cells (ECs) isolated from adult male C57BL/6 mice. (n = 3 per group). **(C)** Relative mRNA levels of AIG1 in CMs, CFs, and ECs, quantified using RT-qPCR. (n = 3 per group). **(D)** Representative immunoblotting images of AIG1 in primary CMs with or without DOX treatment (0.1 μM, 24 h). (n = 3 per group). **(E)** Relative mRNA levels of AIG1 in primary CMs with or without DOX (0.1 μM, 24 h) treatment. (n = 3 per group). **(F)** Representative immunoblotting images of AIG1 in primary CFs with or without DOX treatment (0.1 μM, 24 h). (n = 3 per group). **(G)** Relative mRNA levels of AIG1 in primary CFs with or without DOX (0.1 μM, 24 h) treatment. (n = 3 per group). **(H)** Representative immunoblotting images of AIG1 in primary ECs with or without DOX (0.1 μM, 24 h) treatment. (n = 3 per group). **(I)** Relative mRNA levels of AIG1 in primary ECs with or without DOX (0.1 μM, 24 h) treatment. (n = 3 per group). **(J)** Representative immunoblotting images of AIG1 in mouse heart lysates from male and female mice with or without DOX challenge. (n = 6 per group). **(K)** Relative mRNA levels of AIG1 in mouse heart lysates from male and female mice with or without DOX challenge. (n = 6 per group). Data are presented as Mean ± SEM. ***p < 0.001, ****p < 0.0001. ns, no significance. For statistical analysis, one-way ANOVA with Tukey's test for multiple comparisons was used for **C**; unpaired student's t-test was used for **E**, **G**, and **I**; two-way ANOVA with Tukey's test for multiple comparisons was used for **K**.

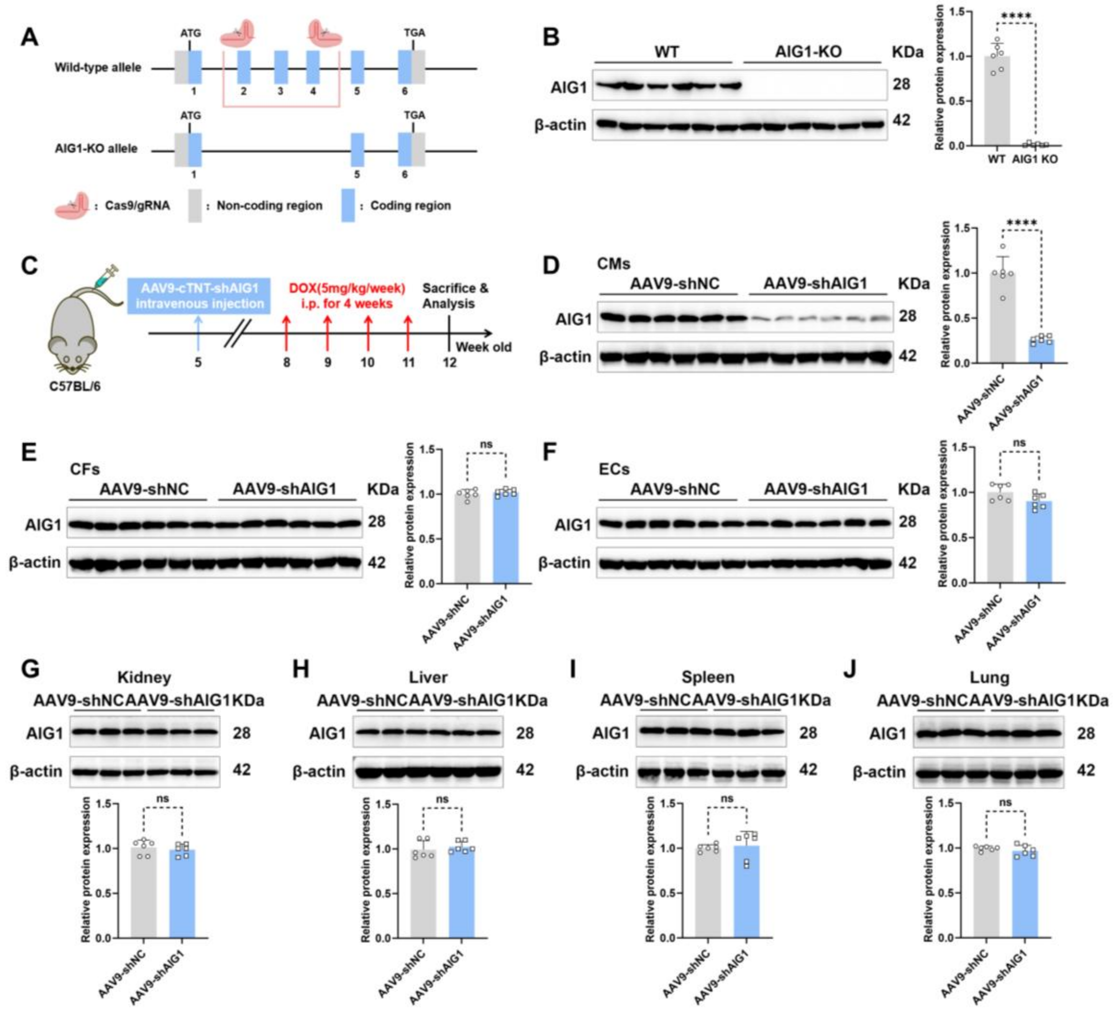

Figure S2: Efficiency of the global knockout and the cardiac-specific knockdown of AIG1 in mice. (A) Strategy for the generation of AIG1 global KO mice. **(B)** Representative immunoblots and statistical analysis of AIG1 in mouse heart lysates from WT and AIG1 KO mice. (n = 6 per group). **(C)** Flow chart depicting experimental strategy for cardiac-specific AIG1 knockdown using AAV9 delivery in the chronic DIC mouse model. **(D)** Representative immunoblots and statistical analysis showing AIG1 protein levels in cardiac CMs isolated from AAV9-shNC and AAV9-shAIG1 mice hearts to validate cardiomyocyte-specific AIG1 knockdown in AAV9-shAIG1 mice. (n = 6 per group). **(E)** and **(F)** Representative immunoblots and statistical analysis showing AIG1 protein levels in cardiac CFs and ECs isolated from AAV9-shNC and AAV9-shAIG1 mice hearts. (n = 6 per group). **(G)** through **(J)** Representative immunoblots and statistical analysis showing AIG1 protein levels in kidney, liver, spleen, and lung tissues of mice injected with AAV9-shAIG1 group or control virus group. (n = 6 per group). Data are presented as Mean ± SEM. ****$p < 0.0001$. ns, no significance. For statistical analysis, unpaired student's t-test was used for **B-J**. i.p., intraperitoneally.

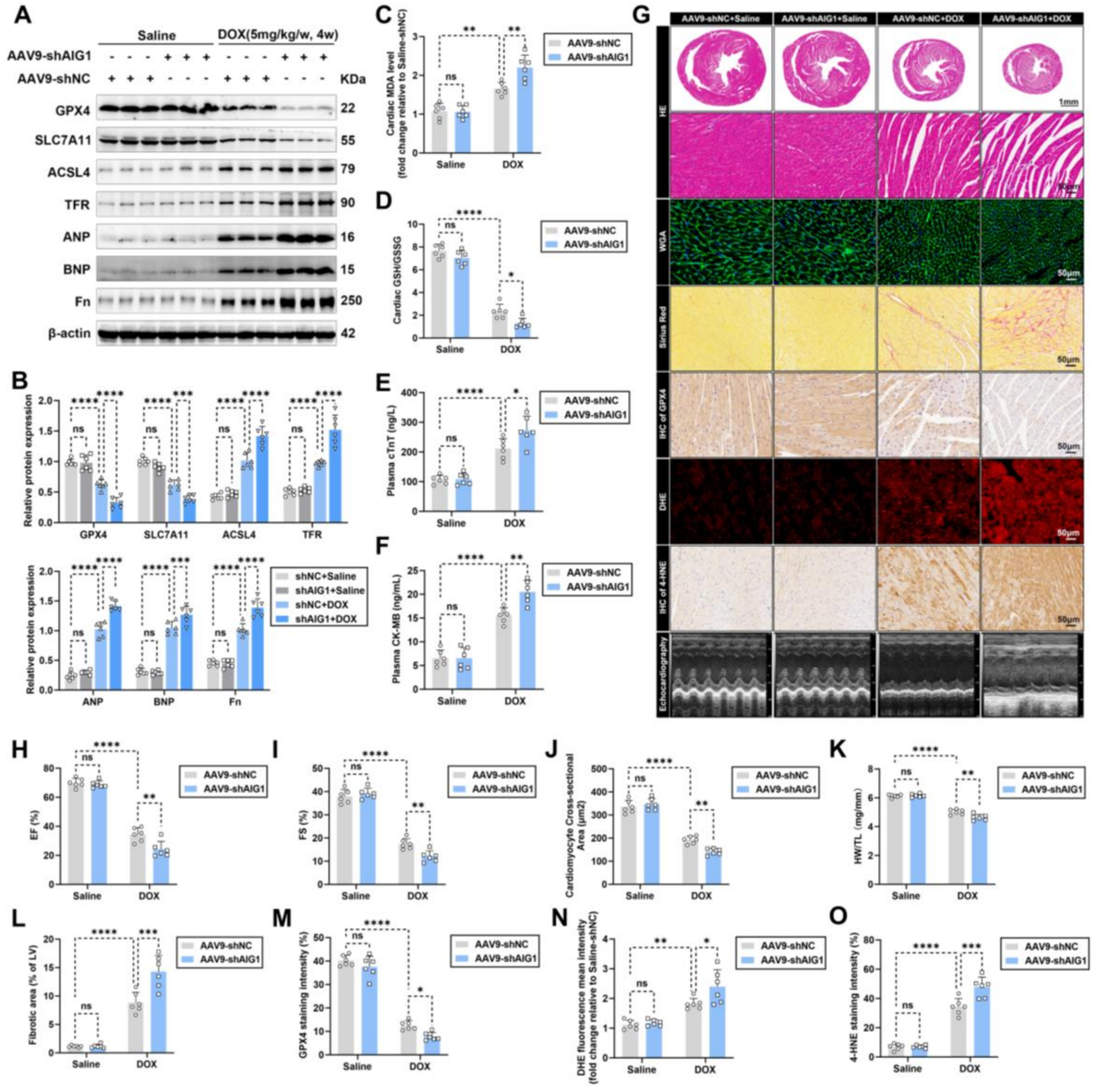

Figure S3: AAV9-mediated cardiac-specific knockdown of AIG1 exacerbates DOX-induced cardiomyocyte ferroptosis and cardiotoxicity in mice. **(A)** and **(B)** Representative immunoblot and statistical analysis of protein expression of ferroptosis markers, ANP, BNP, and fibronectin (Fn) in mouse heart lysates from AAV9-shNC and AAV9-shAIG1 mice with or without DOX challenge. (n = 6 per group). **(C)** MDA levels in heart tissues. (n = 6 per group). **(D)** GSH/GSSG ratio in heart tissues. (n = 6 per group). **(E)** and **(F)** The levels of plasma cTnT and CK-MB in mice. (n = 6 per group). **(G)** through **(I)** Representative M-mode echocardiographic image **(G)** and analysis of LVEF **(H)** and LVFS **(I)** in AAV9-shNC and AAV9-shAIG1 mice with or without DOX challenge. (n = 6 per group). **(G)** and **(J)** Representative images of HE staining and WGA staining in hearts, along with quantitative analysis of cardiomyocyte areas. (n = 6 per group). **(K)** Heart weight (HW)-to-tibial length (TL) ratio in mice. (n = 6 per group). **(G)** and **(L)** Representative images of Picrosirius Red staining in hearts, along with quantitative analysis of cardiac interstitial fibrosis. (n = 6 per group). **(G)** and **(M)** Representative immunohistochemical staining of GPX4 in hearts, along with quantitative analysis of GPX4 staining intensity. (n = 6 per group). **(G)** and **(N)** Representative fluorescence

images and quantification of DHE staining in hearts. (n = 6 per group). **(G)** and **(O)** Representative immunohistochemical staining of 4-hydroxynonenal (4-HNE) in hearts, along with quantitative analysis of 4-HNE staining intensity. (n = 6 per group). Data are presented as Mean ± SEM. *p < 0.05, **p < 0.01, ***p < 0.001, ****p < 0.0001. ns, no significance. For statistical analysis, two-way ANOVA with Tukey's test for multiple comparisons was used for **B-F** and **H-O**.

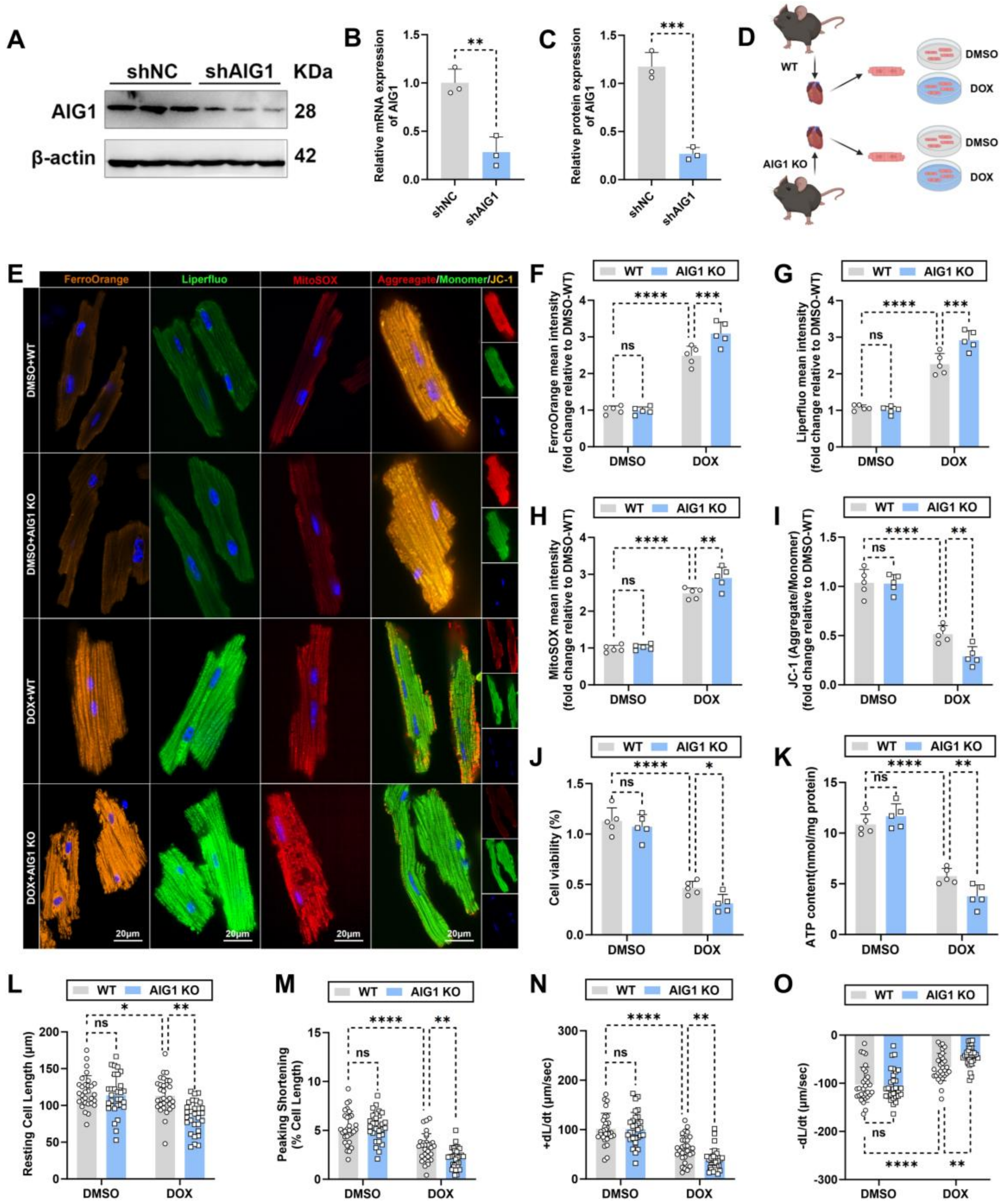

Figure S4: AIG1 deletion aggravates DOX-induced cardiomyocyte ferroptosis and mechanical anomalies. (A) and **(B)** Representative immunoblotting images and statistical analysis demonstrating AIG1 knockdown using adenoviruses carrying shRNA in HL-1 cardiomyocytes. (n = 3 per group). **(C)** Relative mRNA levels of AIG1 in HL-1 cardiomyocytes transfected with adenoviruses carrying shRNA quantified using RT-qPCR. (n = 3 per group). **(D)** Experimental design for DOX treatment (0.1 μM, 24 h) in adult mouse cardiomyocytes (AMCMs) isolated from WT and AIG1 KO mice. **(E)** Representative fluorescence images showing intracellular iron level (FerroOrange staining), lipid peroxide (Liperfluo staining), mitochondrial superoxide (MitoSOX staining), and mitochondrial membrane potential (JC-1 staining) in AMCMs isolated from WT and AIG1 KO mice with or without DOX treatment (0.1 μM, 24 h). Nuclei were counterstained

with Hoechst 33342 (blue). **(F)** Quantification of intracellular iron level (FerroOrange staining) in AMCMs with indicated treatment. (n = 5 per group). **(G)** Quantification of intracellular lipid peroxide (Liperfluo staining) in AMCMs with indicated treatment. (n = 5 per group). **(H)** Quantification of mitochondrial superoxide (MitoSOX staining) in AMCMs with indicated treatment. (n = 5 per group). **(I)** Quantification of mitochondrial membrane potential (JC-1 staining) in AMCMs with indicated treatment. (n = 5 per group). **(J)** Cell viability in AMCMs with indicated treatment. (n = 5 per group). **(K)** ATP content in AMCMs with indicated treatment. (n = 5 per group). **(L)** through **(O)** Cardiomyocyte contractile properties in AMCMs with indicated treatment. (n = 30 per group). **(L)** Resting cell length; **(M)** Peak shortening; **(N)** Maximal velocity of shortening (+dL/dt); **(O)** Maximal velocity of relengthening (−dL/dt). Data are presented as Mean ± SEM. *p < 0.05, **p < 0.01, ***p < 0.001, ****p < 0.0001. ns, no significance. For statistical analysis, unpaired student's t-test was used for **B-C**; two-way ANOVA with Tukey's test for multiple comparisons was used for **F-O**.

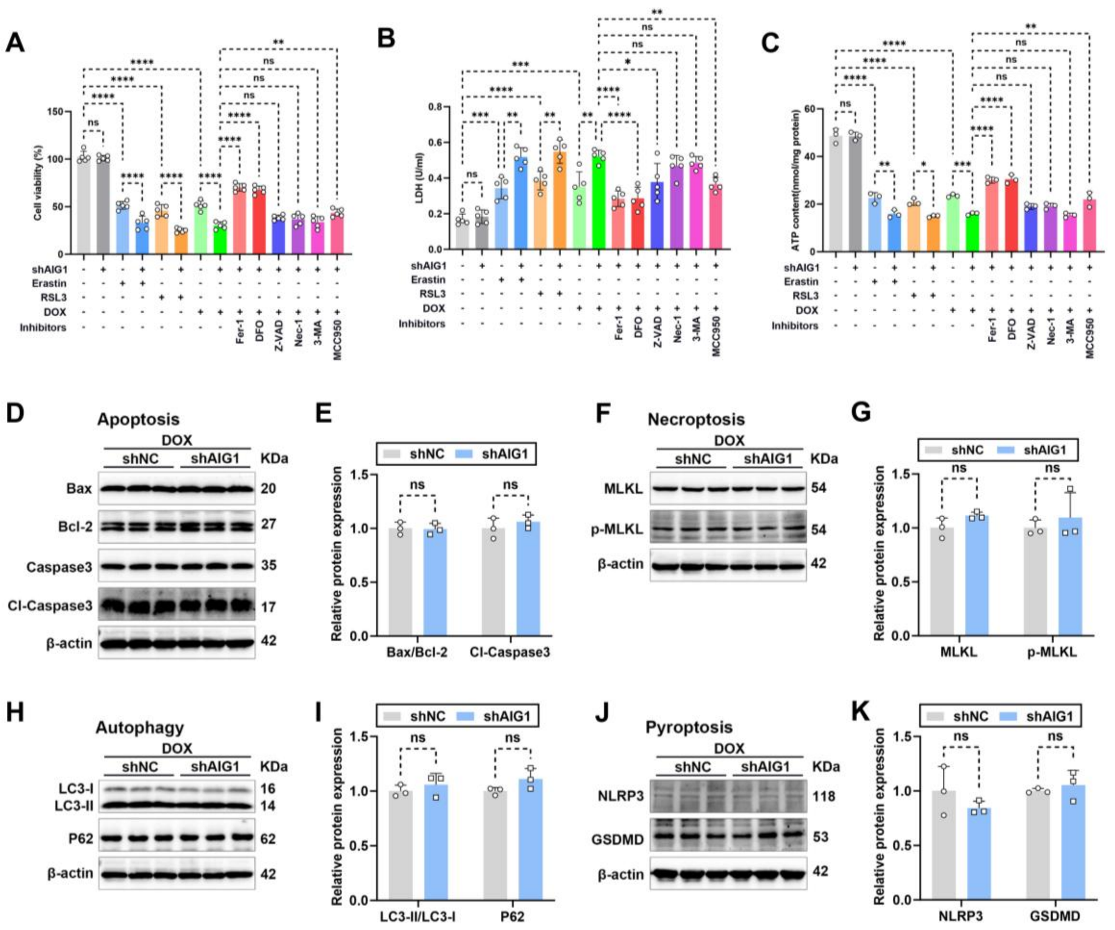

Figure S5: AIG1 knockdown had no effects on apoptosis, necroptosis, autophagy and pyroptosis in DOX-stressed cardiomyocytes. (A) through **(C)** Cell viability, LDH release, and ATP content for HL-1 cardiomyocytes treated with erastin (5 μM), RSL3 (2.5 μM), DOX (1 μM), Fer-1 (2.5 μM), DFO (5 μM), Z-VAD-FMK (10 μM), Nec-1 (2 μM), 3-MA (5 mM), or MCC950 (10 μM) for 24 h in the presence or absence of AIG1 knockdown. (n = 5 per group for cell viability and LDH release, n = 3 per group for ATP content). **(D)** through **(K)** Representative immunoblotting images and statistical analysis of protein expression levels of various cell death markers, such as apoptosis **(D-E)**, necroptosis **(F-G)**, autophagy **(H-I)**, and pyroptosis **(J-K)**, in DOX-treated HL-1 cardiomyocytes with or without AIG1 knockdown. (n = 3 per group). Data are presented as Mean ± SEM. *p < 0.05, **p < 0.01, ***p < 0.001, ****p < 0.0001. ns, no significance. For statistical analysis, one-way ANOVA with Tukey's test for multiple comparisons was used for **A-C**; unpaired student's t-test was used for **E**, **G**, **I**, and **K**.

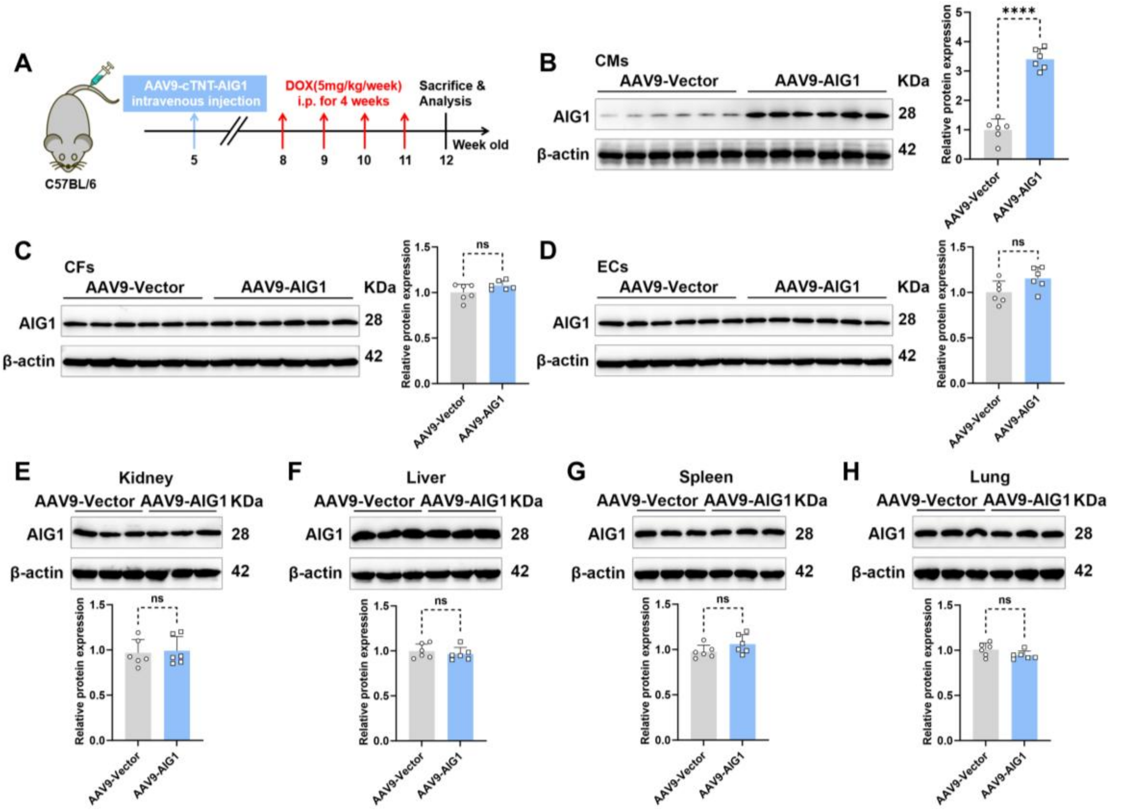

Figure S6: Efficiency of the cardiac-specific overexpression of AIG1 in mice. (A) Flow chart depicting experimental strategy for cardiac-specific AIG1 overexpression using AAV9 delivery in the chronic DIC mouse model. **(B)** Representative immunoblots and statistical analysis showing AIG1 protein levels in cardiac CMs isolated from AAV9-Vector and AAV9-AIG1 mice hearts to validate cardiomyocyte-specific AIG1 overexpression in AAV9-AIG1 mice. (n = 6 per group). **(C)** and **(D)** Representative immunoblots and statistical analysis showing AIG1 protein levels in cardiac CFs and ECs isolated from AAV9-Vector and AAV9-AIG1 mice hearts. (n = 6 per group). **(E)** through **(H)** Representative immunoblots and statistical analysis showing AIG1 protein levels in kidney, liver, spleen, and lung tissues of mice injected with AAV9-AIG1 group or control virus group. (n = 6 per group). Data are presented as Mean ± SEM. ****$p < 0.0001$. ns, no significance. For statistical analysis, unpaired student's t-test was used for **B-H**.

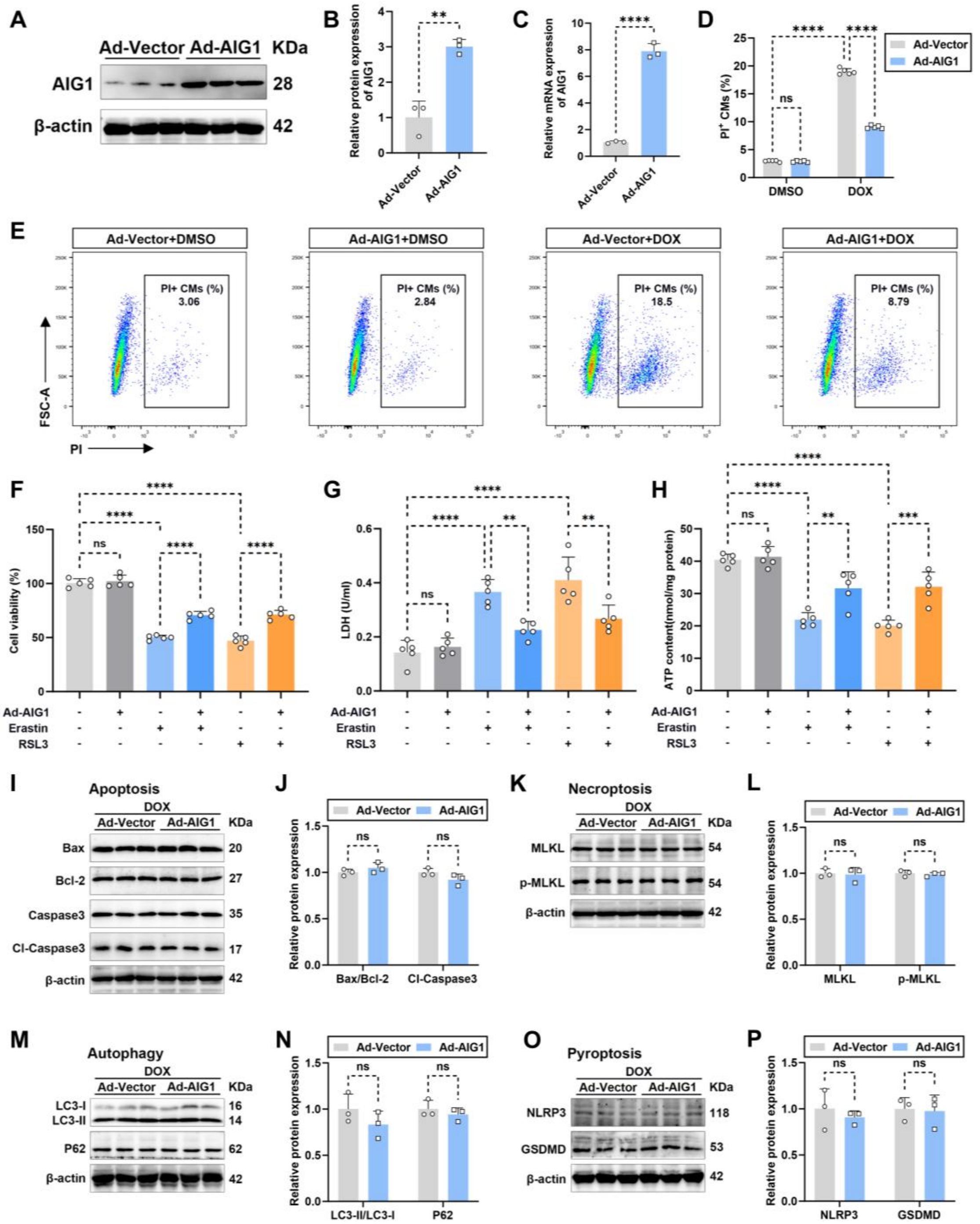

Figure S7: AIG1 overexpression specifically prevents ferroptosis, but not other cell death modalities, in cardiomyocytes under DOX stress. (A) and **(B)** Representative immunoblotting images and statistical analysis demonstrating AIG1 overexpression via adenoviruses delivery in HL-1 cardiomyocytes. (n = 3 per group). **(C)** Relative mRNA levels of AIG1 in HL-1 cardiomyocytes transfected with adenoviruses carrying mouse AIG1 cDNA quantified using RT-qPCR. (n = 3 per group). **(D)** and **(E)** The percentage of PI^+ HL-1 cardiomyocytes was calculated using flow cytometry. (n = 5 per group). **(F)** through **(H)** Cell viability, LDH release, and ATP content for HL-1 cardiomyocytes treated with erastin (5 μM), RSL3 (2.5 μM) for 24 h in the presence or absence of AIG1 overexpression. (n = 5 per group for cell viability and LDH release, n = 3 per group for

ATP content). **(I)** through **(P)** Representative immunoblotting images and statistical analysis of protein expression levels of various cell death markers, such as apoptosis **(I-J)**, necroptosis **(K-L)**, autophagy **(M-N)**, and pyroptosis **(O-P)**, in DOX-treated HL-1 cardiomyocytes with or without AIG1 overexpression. (n = 3 per group). Data are presented as Mean ± SEM. **$p < 0.01$, ***$p < 0.001$, ****$p < 0.0001$. ns, no significance. For statistical analysis, unpaired student's t-test was used for **B-C**, **J**, **L**, **N**, and **P**; two-way ANOVA with Tukey's test for multiple comparisons was used for **D**; one-way ANOVA with Tukey's test for multiple comparisons was used for **F-H**.

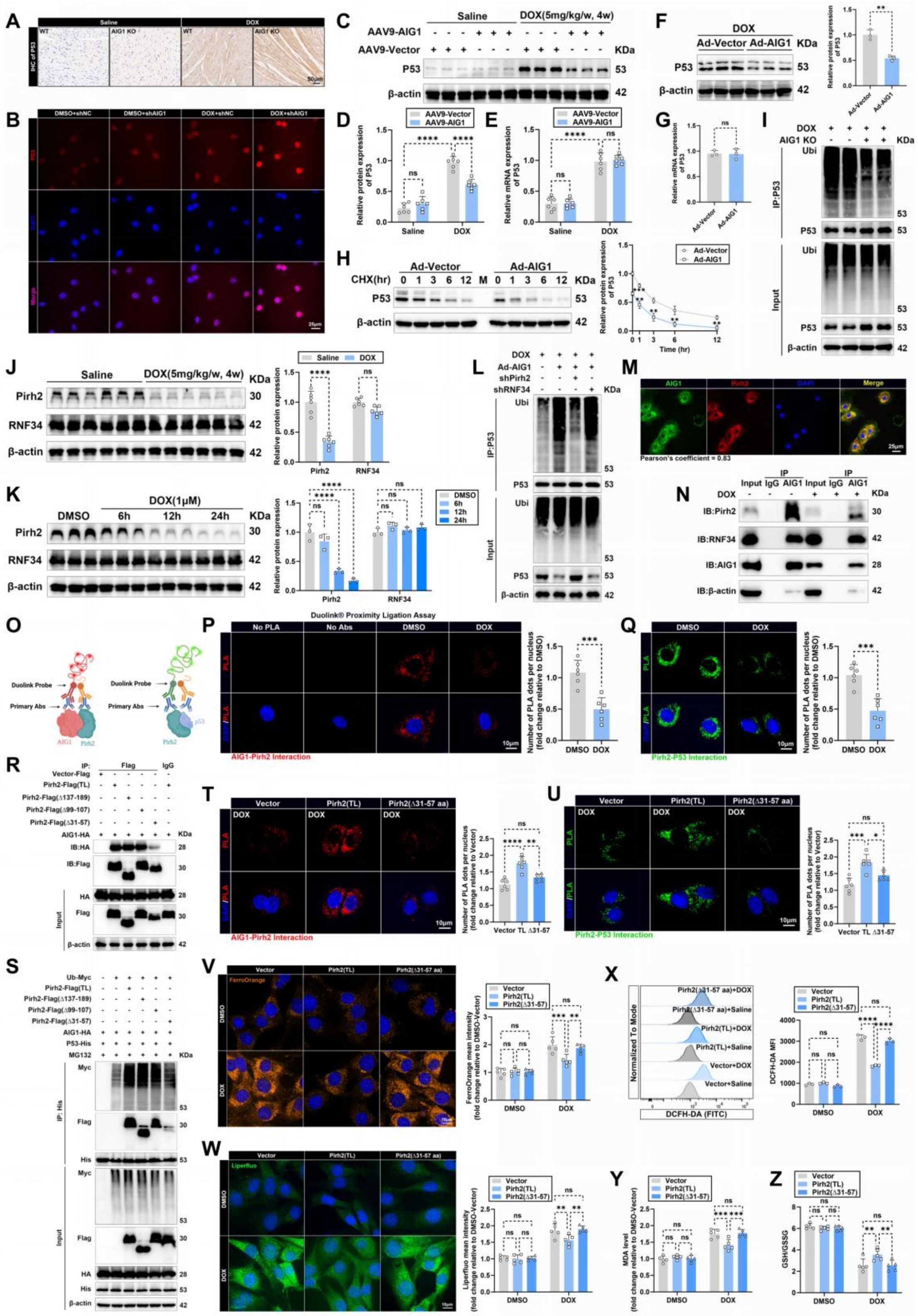

Figure S8: The AIG1-Pirh2 interaction facilitates ubiquitination-mediated p53 degradation, thereby mitigating DOX-induced cardiomyocyte ferroptosis. (A) Representative immunohistochemical staining of p53 in hearts from WT and AIG1 KO mice with or without DOX treatment. **(B)** Representative immunofluorescence staining of p53 in HL-1 cardiomyocytes with indicated treatment. **(C)** and **(D)** Representative

immunoblot and statistical analysis of p53 protein expression in mouse heart lysates from AAV9-Vector and AAV9-AIG1 mice with or without DOX challenge. (n = 6 per group). **(E)** Relative mRNA levels of p53 in mouse heart lysates from AAV9-Vector and AAV9-AIG1 mice with or without DOX challenge quantified using RT-qPCR. (n = 6 per group). **(F)** Representative immunoblotting images and statistical analysis of p53 protein expression in DOX-treated HL-1 cardiomyocytes with or without AIG1 overexpression. (n = 3 per group). **(G)** Relative mRNA levels of p53 in DOX-treated HL-1 cardiomyocytes with or without AIG1 overexpression quantified using RT-qPCR. (n = 3 per group). **(H)** Representative immunoblotting images and quantification of p53 protein level in cycloheximide (CHX) chase assay in HL-1 cardiomyocytes from Ad-Vector and Ad-AIG1 groups under DOX stress. (n = 3 per group). Cells were treated with DOX (1 μM) for 24 h before the addition of CHX for time-series analysis. **(I)** Ubiquitination assays assessing the ubiquitination of endogenous p53 in the hearts of WT and AIG1 KO mice following DOX stress. **(J)** Representative immunoblotting images and quantification of Pirh2 and RNF34 expression in mouse heart lysates with or without DOX challenge. (n = 6 per group). **(K)** Representative immunoblotting images and statistical analysis of Pirh2 and RNF34 expression in DOX-challenged HL-1 cardiomyocytes. (n = 3 per group). **(L)** Ubiquitination assays assessing the ubiquitination of endogenous p53 in DOX stressed HL-1 cardiomyocytes with indicated treatment. **(M)** Representative immunofluorescence images illustrating the colocalization of AIG1 and Pirh2 in HL-1 cardiomyocytes. **(N)** Representative immunoblotting images following Co-immunoprecipitation (Co-IP) using anti-AIG1 in HL-1 cardiomyocytes with or without DOX (1 μM, 24 h) challenge. **(O)** A Schematic representation of proximity ligation assay (PLA) detection and analysis of AIG1 interaction (protein-protein interaction) with Pirh2 and Pirh2 interaction with p53 in HL-1 cardiomyocytes with or without DOX (1 μM, 24 h) treatment. **(P)** Representative images and quantification of PLA analysis demonstrating the interaction between AIG1 and Pirh2 in HL-1 cardiomyocytes. PLA-positive spots are shown in red. Nuclei were counterstained with DAPI (blue). No antibodies: PLA was performed in the absence of primary antibodies. (n = 6 per group). **(Q)** Representative images and quantification of PLA detection showing Pirh2 and p53 interactions in HL-1 cardiomyocytes. Each green dot represents the detection of the protein-protein complex, and nuclei were counterstained with DAPI (blue). (n = 6 per group). **(R)** Representative immunoblotting images of Co-IP assays using an anti-Flag tag in HEK293T cells co-transfected with the indicated constructs. **(S)** Representative immunoblotting images demonstrating the effects of the indicated Pirh2 mutants (Δ31-57, Δ99-107, Δ137-189) on Pirh2-mediated exogenous p53 ubiquitination and its protein level in MG132-treated HEK293T cells co-transfected with the indicated constructs. **(T)** and **(U)** Representative images and quantification of PLA analysis showing the interaction of AIG1 with Pirh2 and Pirh2 with p53 in DOX-treated HL-1 cardiomyocytes transfected with Vector, Pirh2 (TL)-, or Pirh2 (Δ31-57)-overexpressed adenovirus. (n = 6 per group). **(V)** and **(W)** Representative fluorescence images and quantification of FerroOrange staining and Liperfluo staining in HL-1 cardiomyocytes transfected with Vector, Pirh2 (TL)-, or Pirh2 (Δ31-57)-overexpressed adenovirus, with or without DOX (1 μM, 24 h) challenge. Nuclei were counterstained with Hoechst 33342 (blue). (n = 5 per group). **(X)** Representative

histogram showing intracellular ROS level (DCFH-DA staining) in HL-1 cardiomyocytes transfected with Vector, Pirh2 (TL)-, or Pirh2 (Δ31-57)-overexpressed adenovirus, with or without DOX (1 μM, 24 h) challenge, along with quantitative analysis of the mean fluorescence intensities (MFI) using flow cytometry. (n = 3 per group). **(Y)** and **(Z)** MDA levels and GSH/GSSG ratio in HL-1 cardiomyocytes transfected with Vector, Pirh2 (TL)-, or Pirh2 (Δ31-57)-overexpressed adenovirus, with or without DOX (1 μM, 24 h) challenge. (n = 5 per group). Data are presented as Mean ± SEM. *p < 0.05, **p < 0.01, ***p < 0.001, ****p < 0.0001. ns, no significance. For statistical analysis, unpaired student's t-test was used for **F-H**, **J**, and **P-Q**; one-way ANOVA with Tukey's test for multiple comparisons was used for **K** and **T-U**; two-way ANOVA with Tukey's test for multiple comparisons was used for **D-E** and **V-Z**. Abs, antibodies.

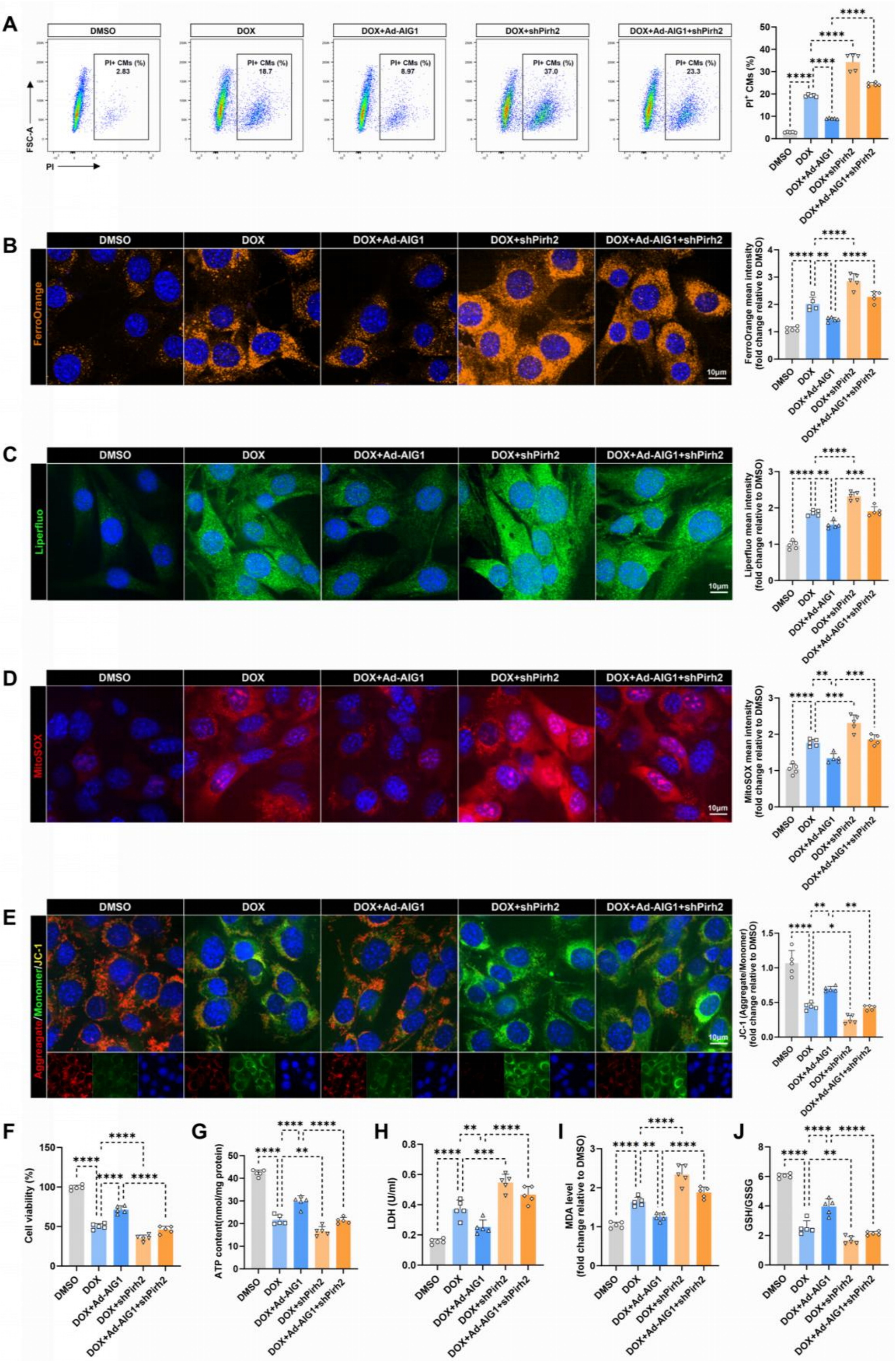

Figure S9: Knockdown of Pirh2 exacerbates DOX-induced cardiomyocyte ferroptosis *in vitro*. **(A)** through **(J)** HL-1 cardiomyocytes were exposed to DOX (1 μM, 24 h) in the absence or presence of AIG1 overexpression or Pirh2 knockdown. **(A)** The

percentage of PI^+ HL-1 cardiomyocytes was calculated using flow cytometry. (n = 5 per group). **(B)** through **(E)** Representative fluorescence images and quantification of FerroOrange staining, Liperfluo staining, MitoSOX staining, and JC-1 staining in HL-1 cardiomyocytes under various treatment settings. Nuclei were counterstained with Hoechst 33342 (blue). (n = 5 per group). **(F)** through **(J)** Cell viability, ATP content, LDH release, MDA levels, and GSH/GSSG ratio in HL-1 cardiomyocytes under various treatment settings. (n = 5 per group). Data are presented as Mean ± SEM. *$p < 0.05$, **$p < 0.01$, ***$p < 0.001$, ****$p < 0.0001$. For statistical analysis, one-way ANOVA with Tukey's test for multiple comparisons was used for **A-J**.

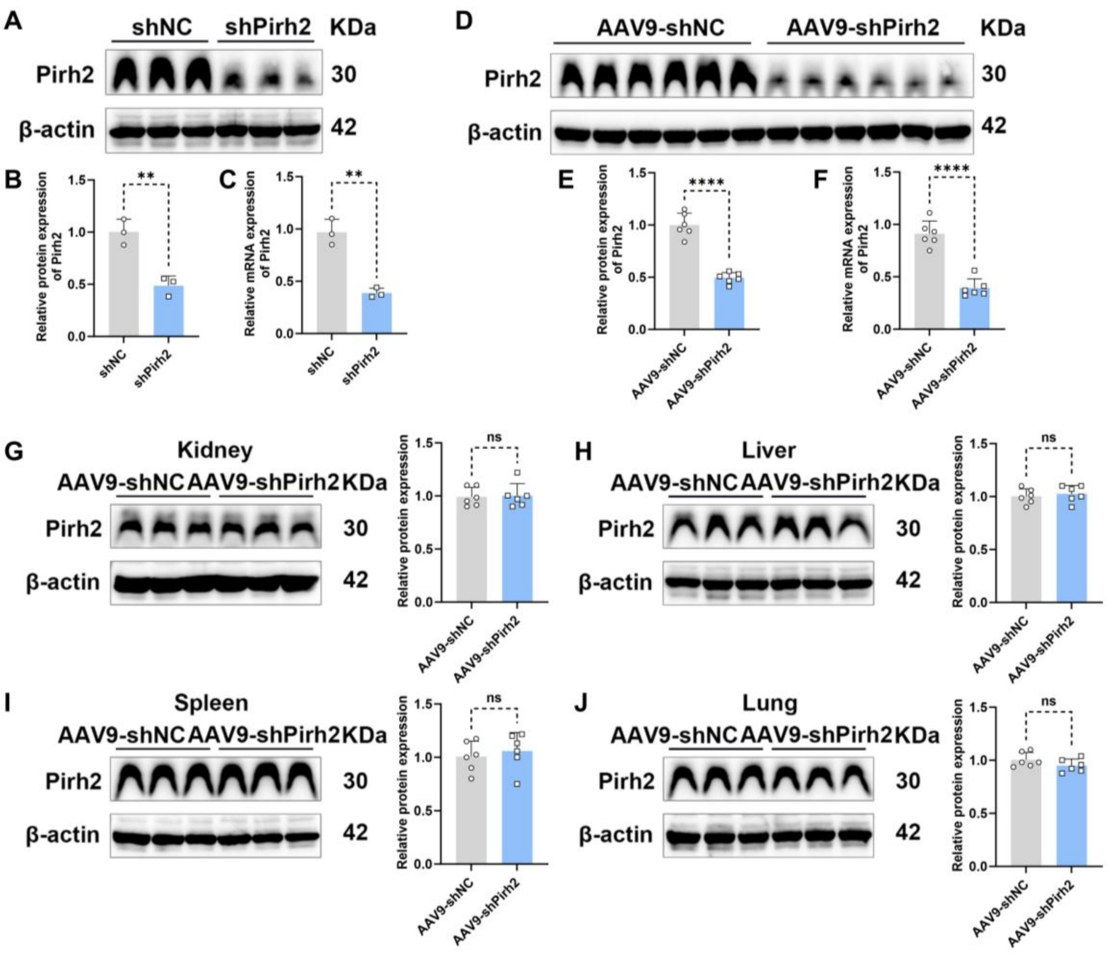

Figure S10: Efficiency of Pirh2 knockdown in HL-1 cardiomyocytes and the cardiac-specific knockdown of Pirh2 in mice. (A) and **(B)** Representative immunoblotting images and statistical analysis demonstrating Pirh2 knockdown using adenoviruses carrying shRNA in HL-1 cardiomyocytes. (n = 3 per group). **(C)** Relative mRNA levels of Pirh2 in HL-1 cardiomyocytes transfected with adenoviruses carrying shRNA quantified using RT-qPCR. (n = 3 per group). **(D)** and **(E)** Representative immunoblots and statistical analysis showing Pirh2 protein levels in cardiac tissues of mice injected with AAV9-cTnT-shPirh2 group or control AAV9-cTnT-shNC group. (n = 6 per group). **(F)** Relative mRNA levels of Pirh2 in cardiac tissues of mice injected with AAV9-cTnT-shPirh2 group or control AAV9-cTnT-shNC group quantified using RT-qPCR. (n = 6 per group). **(G)** through **(J)** Representative immunoblots and statistical analysis showing Pirh2 protein levels in kidney, liver, spleen, and lung tissues of mice injected with AAV9-shPirh2 group or control virus group. (n = 6 per group). Data are presented as Mean ± SEM. $^{**}p < 0.01$, $^{****}p < 0.0001$. ns, no significance. For statistical analysis, unpaired student's t-test was used for **B-C** and **E-J**.

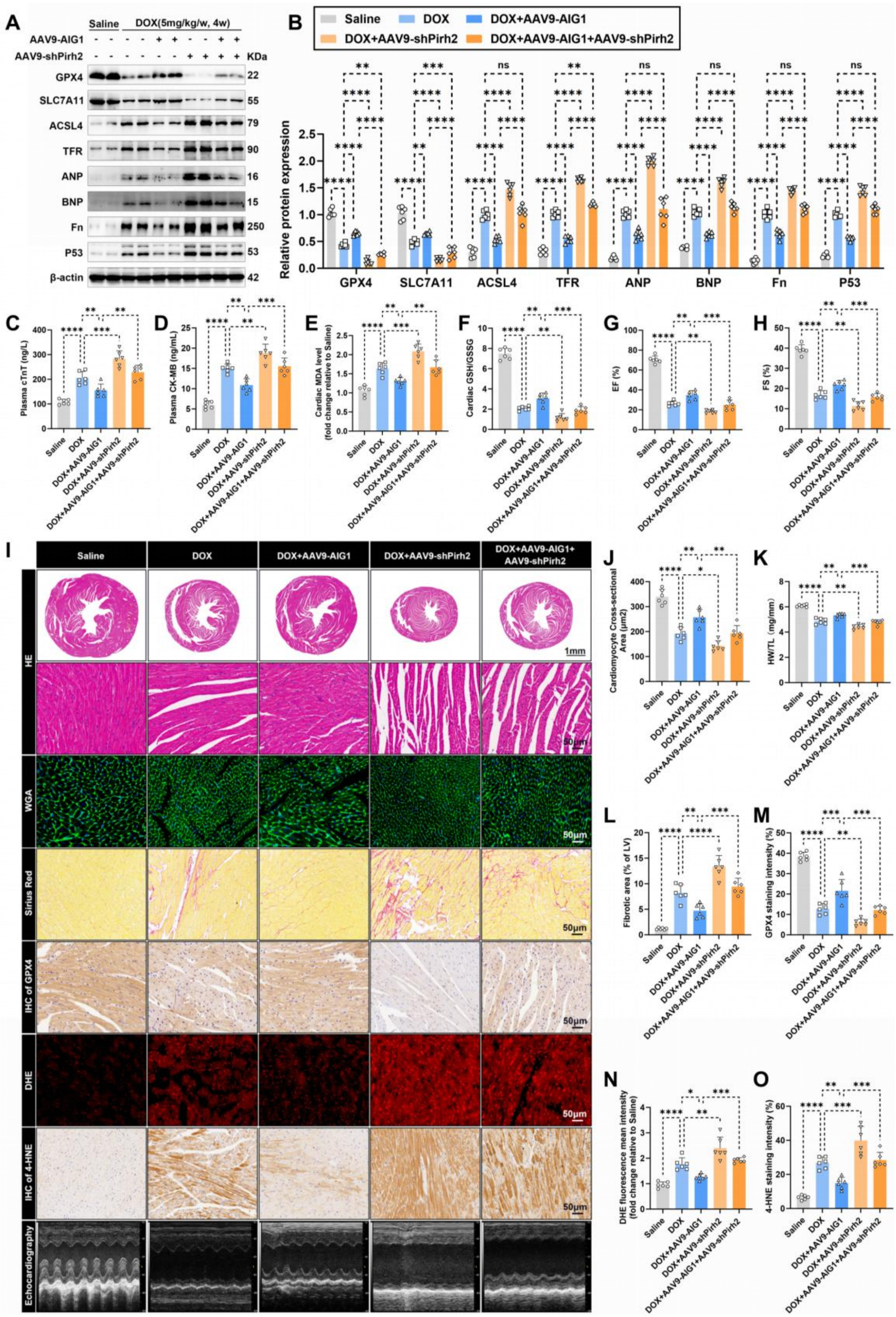

Figure S11: Cardiac-specific knockdown of Pirh2 abrogates the anti-ferroptosis and cardioprotective effects mediated by AIG1 overexpression in mice. (A) and **(B)** Representative immunoblot and statistical analysis of protein expressions of ferroptosis markers, ANP, BNP, and Fn in heart lysates of mice with indicated treatments. **(C)** and **(D)**

Plasma levels of cTnT and CK-MB in mice. **(E)** and **(F)** MDA levels and GSH/GSSG ratio in heart tissues. **(G)** through **(I)** Quantification of LVEF **(G)** and LVFS **(H)** shown with representative M-mode images **(I)** from transthoracic echocardiography in mice. **(I)** and **(J)** Representative HE staining and WGA staining of hearts and quantitative analysis of cardiomyocyte areas in mice. **(K)** Heart weight (HW)-to-tibial length (TL) ratio in mice. **(I)** and **(L)** Representative Picrosirius Red staining images of hearts and quantitative analysis of cardiac interstitial fibrosis in mice. **(I)** and **(M)** Representative immunohistochemical staining of GPX4 in mouse hearts and quantitative analysis of GPX4 staining intensity. **(I)** and **(N)** Representative fluorescence images and quantification of DHE staining in mouse hearts. **(I)** and **(O)** Representative immunohistochemical staining of 4-HNE in mouse hearts and quantitative analysis of 4-HNE staining intensity. Data are presented as Mean ± SEM, n = 6 per group. *p < 0.05, **p < 0.01, ***p < 0.001, ****p < 0.0001. ns, no significance. For statistical analysis, two-way ANOVA with Tukey's test for multiple comparisons was used for **B**; one-way ANOVA with Tukey's test for multiple comparisons was used for **C-H** and **J-O**.

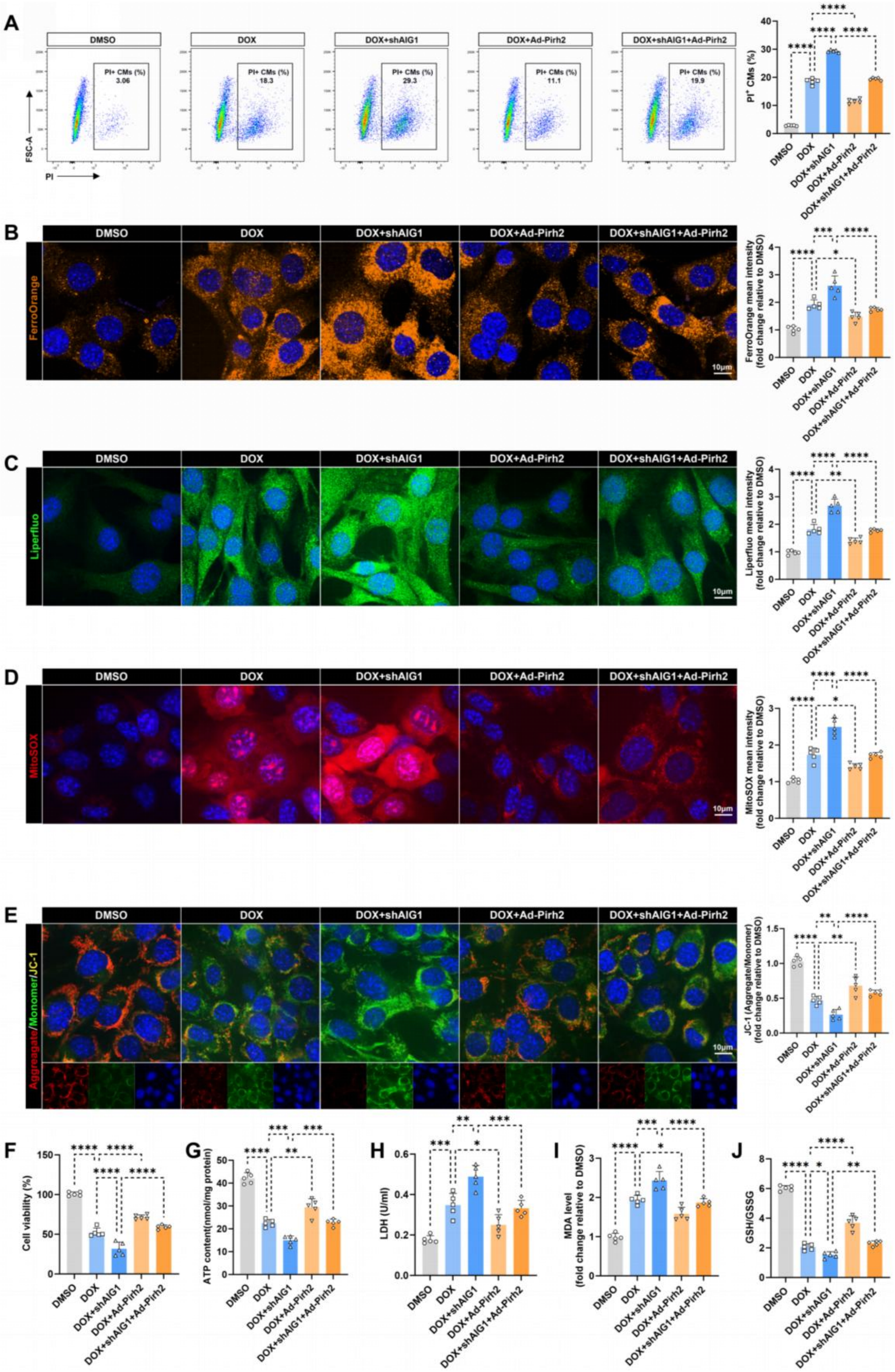

Figure S12: Overexpression of Pirh2 ameliorates DOX-induced cardiomyocyte ferroptosis *in vitro*. **(A)** through **(J)** HL-1 cardiomyocytes were exposed to DOX (1 μM, 24 h) in the absence or presence of AIG1 knockdown or Pirh2 overexpression. **(A)** The

percentage of PI^+ HL-1 cardiomyocytes was calculated using flow cytometry. (n = 5 per group). **(B)** through **(E)** Representative fluorescence images and quantification of FerroOrange staining, Liperfluo staining, MitoSOX staining, and JC-1 staining in HL-1 cardiomyocytes under various treatment settings. Nuclei were counterstained with Hoechst 33342 (blue). **(F)** through **(J)** Cell viability, ATP content, LDH release, MDA levels, and GSH/GSSG ratio in HL-1 cardiomyocytes under various treatment settings. (n = 5 per group). Data are presented as Mean ± SEM. *$p < 0.05$, **$p < 0.01$, ***$p < 0.001$, ****$p < 0.0001$. For statistical analysis, one-way ANOVA with Tukey's test for multiple comparisons was used for **A-J**.

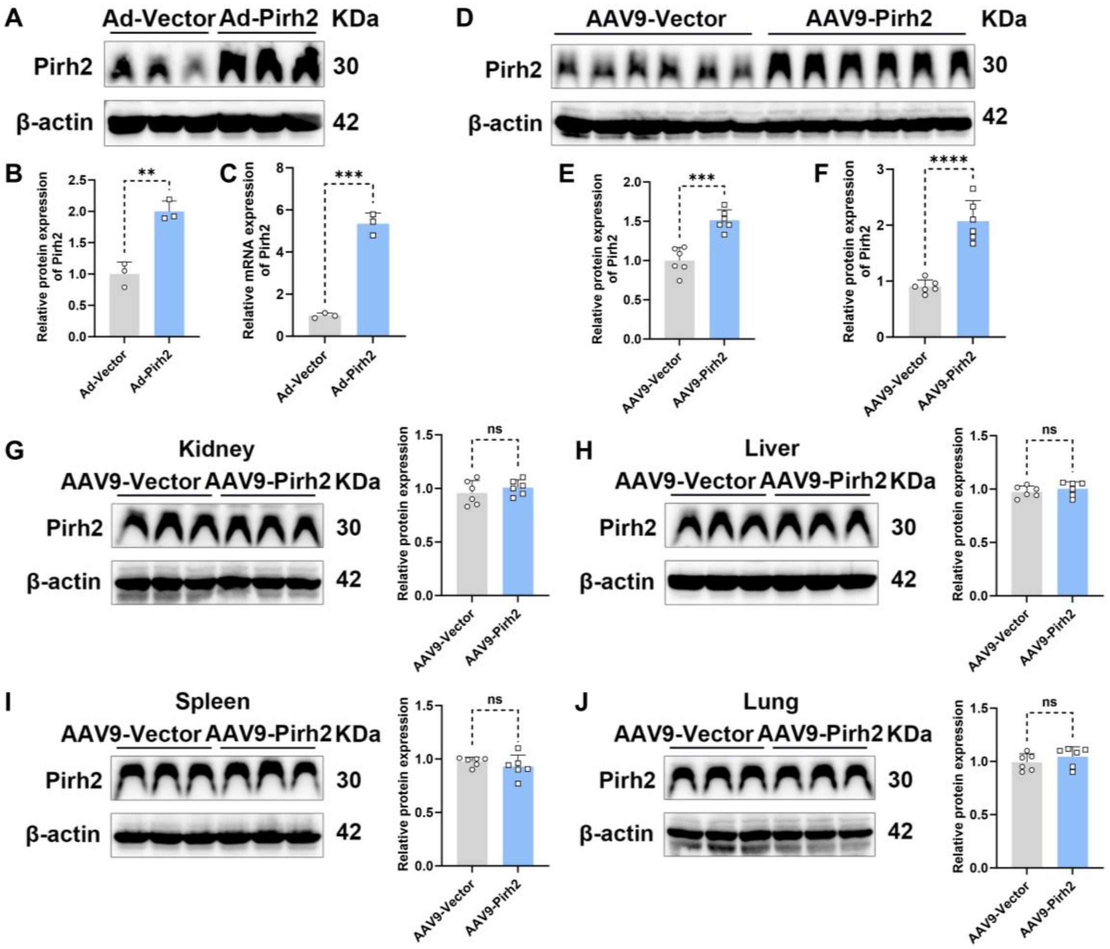

Figure S13: Efficiency of Pirh2 overexpression in HL-1 cardiomyocytes and the cardiac-specific overexpression of Pirh2 in mice. **(A)** and **(B)** Representative immunoblotting images and statistical analysis demonstrating Pirh2 overexpression via adenoviruses delivery in HL-1 cardiomyocytes. (n = 3 per group). **(C)** Relative mRNA levels of AIG1 in HL-1 cardiomyocytes transfected with adenoviruses carrying mouse Pirh2 cDNA quantified using RT-qPCR. (n = 3 per group). **(D)** and **(E)** Representative immunoblots and statistical analysis showing Pirh2 protein levels in cardiac tissues of mice injected with AAV9-cTnT-Pirh2 group or control AAV9-cTnT-Vector group. (n = 6 per group). **(F)** Relative mRNA levels of Pirh2 in cardiac tissues of mice injected with AAV9-cTnT-Pirh2 group or control AAV9-cTnT-shNC group quantified using RT-qPCR. (n = 6 per group). **(G)** through **(J)** Representative immunoblots and statistical analysis showing Pirh2 protein levels in kidney, liver, spleen, and lung tissues of mice injected with AAV9-Pirh2 group or control virus group. (n = 6 per group). Data are presented as Mean ± SEM. **p < 0.01, ***p < 0.001, ****p < 0.0001. ns, no significance. For statistical analysis, unpaired student's t-test was used for **B-C** and **E-J**.

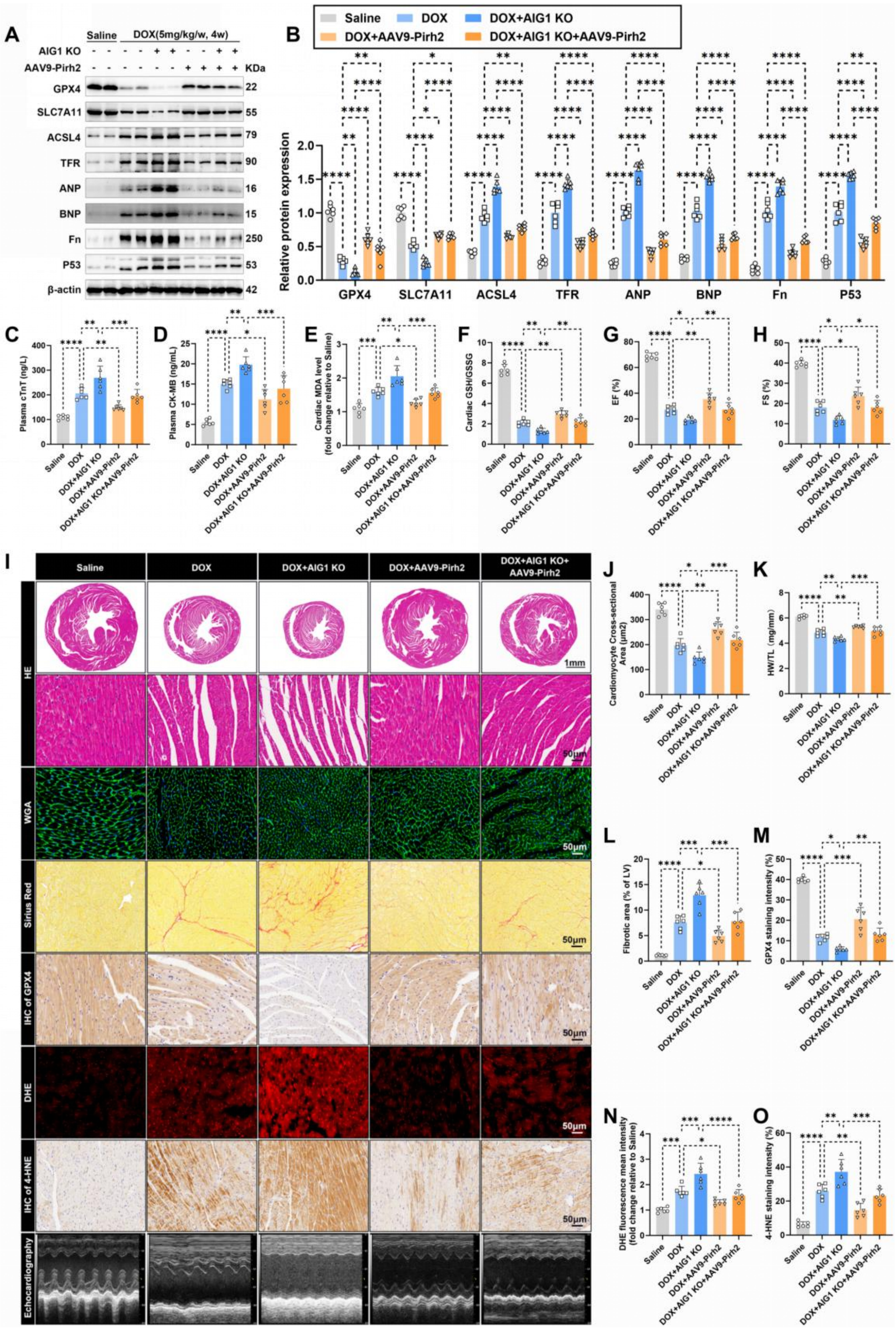

Figure S14: Cardiac-specific overexpression of Pirh2 alleviates DOX-induced cardiomyocyte ferroptosis and cardiotoxicity, reversing AIG1 knockout-mediated cardiac detrimental effects in mice. (A) and **(B)** Representative immunoblot and statistical analysis of protein expressions of ferroptosis markers, ANP, BNP, and Fn in

heart lysates of mice with indicated treatments. **(C)** and **(D)** Plasma levels of cTnT and CK-MB in mice. **(E)** and **(F)** MDA levels and GSH/GSSG ratio in heart tissues. **(G)** through **(I)** Quantification of LVEF **(G)** and LVFS **(H)** shown with representative M-mode images **(I)** from transthoracic echocardiography in mice. **(I)** and **(J)** Representative HE staining and WGA staining of hearts and quantitative analysis of cardiomyocyte areas in mice. **(K)** Heart weight (HW)-to-tibial length (TL) ratio in mice. **(I)** and **(L)** Representative Picrosirius Red staining images of hearts and quantitative analysis of cardiac interstitial fibrosis in mice. **(I)** and **(M)** Representative immunohistochemical staining of GPX4 in mouse hearts and quantitative analysis of GPX4 staining intensity. **(I)** and **(N)** Representative fluorescence images and quantification of DHE staining in mouse hearts. **(I)** and **(O)** Representative immunohistochemical staining of 4-HNE in mouse hearts and quantitative analysis of 4-HNE staining intensity. Data are presented as Mean ± SEM, n = 6 per group. *p < 0.05, **p < 0.01, ***p < 0.001, ****p < 0.0001. For statistical analysis, two-way ANOVA with Tukey's test for multiple comparisons was used for **B**; one-way ANOVA with Tukey's test for multiple comparisons was used for **C-H** and **J-O**.

Supplementary Tables

Table S1. gRNA target sequences of *Aig1* used in CRISPR-Cas9

gRNA No.	gRNA Sequence (5'-3')	PAM
5S1	ATCTTGTGAAGGTGTCTAGC	AGG
3S1	GGGCTTGCAGTGCGTTCATG	AGG

Table S2. shRNA sequences

Gene name	Target Sequences (5'-3')
Aig1	GCTCCATCCTGTGCAACTACA
Rchy1	GGGCATCTCCTACATAGAACG
Rnf34	GGCACAGGTACAAAGTGAAAT

Table S3. Sequences of Real-time qPCR primers

Gene Name	Sequences
Aig1 (mouse)	Forward: 5'-ACCCAATCTTCTCTAAGCTGTGC-3'<br>Reverse: 5'-TCCCACCTTCCAGCATGAATG-3'
p53 (mouse)	Forward: 5'-GACCGCCGTACAGAAGAAGA-3'<br>Reverse: 5'-GCGGATCTTGAGGGTGAAATA-3'
Rchy1 (mouse)	Forward: 5'-GCCTAACCACGAATCTTCGAGG-3'<br>Reverse: 5'-ACACGGCAAGACGTGAGCAACA-3'
Rnf34 (mouse)	Forward: 5'-CCTGGCACAGGTACAAAGTG-3'<br>Reverse: 5'-AAGGCTTGAGAGGTCAGACA-3'
Actb (mouse)	Forward: 5'-GTGACGTTGACATCCGTAAAGA-3'<br>Reverse: 5'-GCCGGACTCATCGTACTCC-3'

Table S4. Information of antibodies for Western blot analysis

Name	Source	Catalog number
anti-AIG1	Abcam, USA	ab140186
anti-GPX4	Proteintech, Wuhan, China	67763-1-Ig
anti-SLC7A11	Proteintech, Wuhan, China	26864-1-AP
anti-TFR	Proteintech, Wuhan, China	10084-2-AP
anti-ACSL4	Proteintech, Wuhan, China	22401-1-AP
anti-SOD2	Proteintech, Wuhan, China	24127-1-AP
anti-HO-1	Proteintech, Wuhan, China	10701-1-AP
anti-NRF2	Proteintech, Wuhan, China	16396-1-AP
anti-Fibronectin	Proteintech, Wuhan, China	15613-1-AP
anti-NPPA	ABclonal, Wuhan, China	A14755
anti-BNP	ABclonal, Wuhan, China	A2179
anti-TGFβ1	Abcam, USA	ab215715
anti-COL3A1	Proteintech, Wuhan, China	68320-1-Ig
anti-P53	Proteintech, Wuhan, China	10442-1-AP
anti-Pirh2	Proteintech, Wuhan, China	67856-1-Ig
anti-RNF34	Abcam, USA	ab169047
anti-HA tag	Proteintech, Wuhan, China	51064-2-AP
anti-DYKDDDDK tag	Proteintech, Wuhan, China	20543-1-AP
anti-Myc tag	Proteintech, Wuhan, China	16286-1-AP
anti-6*HIS tag	Proteintech, Wuhan, China	66005-1-Ig
anti-ubiquitin	Proteintech, Wuhan, China	80992-1-RR
anti-HRP-conjugated Beta Actin	Proteintech, Wuhan, China	HRP-60008
Goat Anti-rabbit IgG	Cell Signaling Technology	7074
Horse Anti-mouse IgG	Cell Signaling Technology	7076

References

1. Wu L, Du Y, Wang L, Zhang Y, Ren J. Inhibition of METTL3 ameliorates doxorubicin-induced cardiotoxicity through suppression of TFRC-mediated ferroptosis. Redox Biol. 2024; 72: 103157.
2. Wang B, Jin Y, Liu J, Liu Q, Shen Y, Zuo S, et al. EP1 activation inhibits doxorubicin-cardiomyocyte ferroptosis via Nrf2. Redox Biol. 2023; 65: 102825.
3. Liu X, Chua CC, Gao J, Chen Z, Landy CLC, Hamdy R, et al. Pifithrin-alpha protects against doxorubicin-induced apoptosis and acute cardiotoxicity in mice. Am J Physiol Heart Circ Physiol. 2004; 286: H933-H9.
4. Tovar C, Rosinski J, Filipovic Z, Higgins B, Kolinsky K, Hilton H, et al. Small-molecule MDM2 antagonists reveal aberrant p53 signaling in cancer: implications for therapy. Proc Natl Acad Sci U S A. 2006; 103: 1888-93.
5. Vassilev LT, Vu BT, Graves B, Carvajal D, Podlaski F, Filipovic Z, et al. In vivo activation of the p53 pathway by small-molecule antagonists of MDM2. Science. 2004; 303: 844-8.
6. Gao P, Cao M, Jiang X, Wang X, Zhang G, Tang X, et al. Cannabinoid Receptor 2-Centric Molecular Feedback Loop Drives Necroptosis in Diabetic Heart Injuries. Circulation. 2023; 147: 158-74.
7. Fang X, Wang H, Han D, Xie E, Yang X, Wei J, et al. Ferroptosis as a target for protection against cardiomyopathy. Proc Natl Acad Sci U S A. 2019; 116: 2672-80.
8. Ta N, Qu C, Wu H, Zhang D, Sun T, Li Y, et al. Mitochondrial outer membrane protein FUNDC2 promotes ferroptosis and contributes to doxorubicin-induced cardiomyopathy. Proc Natl Acad Sci U S A. 2022; 119: e2117396119.
9. Yang M, Abudureyimu M, Wang X, Zhou Y, Zhang Y, Ren J. PHB2 ameliorates Doxorubicin-induced cardiomyopathy through interaction with NDUFV2 and restoration of mitochondrial complex I function. Redox Biol. 2023; 65: 102812.
10. Michalski R, Michalowski B, Sikora A, Zielonka J, Kalyanaraman B. On the use of fluorescence lifetime imaging and dihydroethidium to detect superoxide in intact animals and ex vivo tissues: a reassessment. Free Radic Biol Med. 2014; 67: 278-84.
11. Li C, Zhao Y, Li F, Wang Z, Qiu Z, Yang Y, et al. Semaphorin3A Exacerbates Cardiac Microvascular Rarefaction in Pressure Overload-Induced Heart Disease. Adv Sci (Weinh). 2023; 10: e2206801.
12. Yuan S, Wang Z, Yao S, Wang Y, Xie Z, Wang J, et al. Knocking out USP7 attenuates cardiac fibrosis and endothelial-to-mesenchymal transition by destabilizing SMAD3 in mice with heart failure with preserved ejection fraction. Theranostics. 2024; 14: 5793-808.
13. Luo W, Zou X, Wang Y, Dong Z, Weng X, Pei Z, et al. Critical Role of the cGAS-STING Pathway in Doxorubicin-Induced Cardiotoxicity. Circ Res. 2023; 132: e223-e42.
14. Jumper J, Evans R, Pritzel A, Green T, Figurnov M, Ronneberger O, et al. Highly accurate protein structure prediction with AlphaFold. Nature. 2021; 596: 583-9.
15. Tunyasuvunakool K, Adler J, Wu Z, Green T, Zielinski M, Žídek A, et al. Highly accurate protein structure prediction for the human proteome. Nature. 2021; 596: 590-6.
16. Yan Y, Zhang D, Zhou P, Li B, Huang S-Y. HDOCK: a web server for protein-protein and protein-DNA/RNA docking based on a hybrid strategy. Nucleic Acids Res. 2017; 45: W365-W73.
